# Identifying and Assessing Putative Allosteric Sites and Modulators for CXCR4 Predicted through Network Modeling and Site Identification by Ligand Competitive Saturation

**DOI:** 10.1021/acs.jpcb.4c00925

**Published:** 2024-04-22

**Authors:** Tugce Inan, Robin Flinko, George K. Lewis, Alexander D. MacKerell, Ozge Kurkcuoglu

**Affiliations:** † Department of Chemical Engineering, Istanbul Technical University, Istanbul 34469, Turkey; ‡ Institute of Human Virology, University of Maryland School of Medicine, Baltimore, Maryland 21201, United States; § University of Maryland Computer-Aided Drug Design Center, Department of Pharmaceutical Sciences, School of Pharmacy, University of Maryland, Baltimore, Maryland 21201, United States

## Abstract

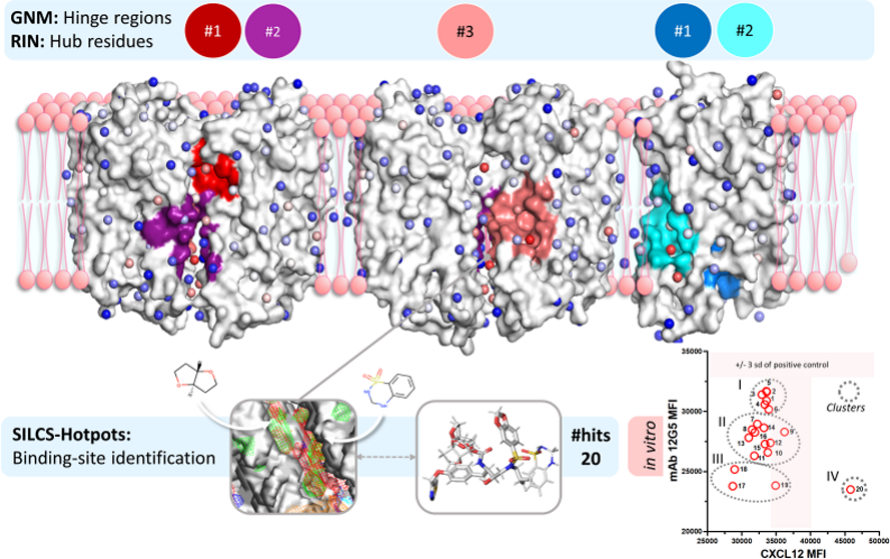

The chemokine receptor CXCR4 is a critical target for the treatment of several cancer types and HIV-1 infections. While orthosteric and allosteric modulators have been developed targeting its extracellular or transmembrane regions, the intramembrane region of CXCR4 may also include allosteric binding sites suitable for the development of allosteric drugs. To investigate this, we apply the Gaussian Network Model (GNM) to the monomeric and dimeric forms of CXCR4 to identify residues essential for its local and global motions located in the hinge regions of the protein. Residue interaction network (RIN) analysis suggests hub residues that participate in allosteric communication throughout the receptor. Mutual residues from the network models reside in regions with a high capacity to alter receptor dynamics upon ligand binding. We then investigate the druggability of these potential allosteric regions using the site identification by ligand competitive saturation (SILCS) approach, revealing two putative allosteric sites on the monomer and three on the homodimer. Two screening campaigns with Glide and SILCS-Monte Carlo docking using FDA-approved drugs suggest 20 putative hit compounds including antifungal drugs, anticancer agents, HIV protease inhibitors, and antimalarial drugs. *In vitro* assays considering mAB 12G5 and CXCL12 demonstrate both positive and negative allosteric activities of these compounds, supporting our computational approach. However, *in vivo* functional assays based on the recruitment of β-arrestin to CXCR4 do not show significant agonism and antagonism at a single compound concentration. The present computational pipeline brings a new perspective to computer-aided drug design by combining conformational dynamics based on network analysis and cosolvent analysis based on the SILCS technology to identify putative allosteric binding sites using CXCR4 as a showcase.

## Introduction

G-protein-coupled receptors (GPCRs) are transmembrane domain receptors that initiate internal signaling pathways via binding to their specific ligands. Chemokine or chemotactic cytokine receptors are members of the GPCR family, and their biological roles are related to cell migration and homeostasis.^1^ CXCR4 is closely associated with immunomodulation, organogenesis, and hematopoiesis,^2,3^ and is overexpressed in numerous human cancers such as kidney, lung, brain, prostate, breast, pancreas, ovarian, and melanoma.^4−6^ The signaling of CXCR4 and its ligand CXCL12 (also known as SDF-1) promotes tumor cell proliferation and guides the formation of distant metastasis.^7^ Recent studies also indicate that the CXCR4/CXCL12 signal axis induces tissue regeneration and wound healing.^8,9^ Moreover, CXCR4 is the second coreceptor of HIV-1 for cellular entry into CD4+ cells through binding of the viral envelope glycoprotein gp120.^10^ Being a part of numerous vital activities in the human body makes CXCR4 an outstanding target for therapeutic purposes.

Signal transduction is accomplished by both monomeric and dimeric GPCRs from families A and B.^11^ CXCR4 can reside in cell membranes as a mixture of monomers, dimers, and higher-order oligomers, with the ensemble of oligomeric states being quite complicated when receptor expression levels increase.^12^ CXCR4 is a monomer at a low expression level. However, increasing CXCR4 concentration in the cell membrane promotes receptor homodimerization.^13^ While CXCR4 is overexpressed in many tumor cells,^5,6^ it has been detected in the homodimer form in malignant cells.^14^ Consequently, both forms of CXCR4 should be investigated for drug design and targeting.

There exist several orthosteric and allosteric modulators available for CXCR4 for different purposes. AMD3100 is a competitive orthosteric antagonist of CXCR4 introduced as an anti-HIV-1 agent,^15^ which was later approved by the FDA for its use in autologous transplantation in patients with non-Hodgkin’s lymphoma or multiple myeloma.^16^ AMD070, also known as Mavorixafor, is a selective allosteric inhibitor of CXCR4 against HIV infection.^17^ In addition, the CXCR4 antagonists induce the mobilization of hematopoietic stem cells, of which transplantation is an effective treatment for many diseases.^18,19^ Pepducin ATI-2341 and peptide-based ligands are known allosteric agonists for CXCR4 to mobilize bone marrow polymorphonuclear neutrophils.^20,21^ Pepducins have been experimentally validated;^21−23^ they are allosteric agonists/antagonists that bind to the intracellular region to mobilize bone marrow hematopoietic cells.^24^ On the other hand, Na^+^ ions have been defined as negative allosteric modulators that stabilize CXCR4 in its inactive state.^25−27^ The benefits of allosteric drugs are well defined, notably their higher specificity accompanied by fewer side effects, and, aside from expanding the drug repertoire, they can improve the affinity and potency of available therapeutics.^28^

Given the importance of CXCR4 in different diseases, the structure–function relationship of CXCR4 has been studied using computational techniques to understand its functional dynamics after ligand binding^29−31^ and the effects of the mutations on its activation.^27,32^ Its tendency to oligomerize with itself and other proteins, which offers additional targets for the design of novel inhibitors, has been also studied using computational and experimental techniques.^33−35^ Allosteric behaviors of CXCR4 have been investigated^36^ and several modulators have been developed.^20,21,36−39^ Modulator binding sites are often located on the extracellular or transmembrane regions of the protein. However, CXCR4 may also accommodate intramembrane binding sites as observed with the free fatty acid receptor GPR40,^40−42^ purinergic P2Y1 receptor,^43^ protease-activated receptor 2 PAR2,^44^ and human melatonin receptors MT1 and MT2,^45,46^ which are all GPCRs with intramembrane binding sites. Recent biophysical and computational studies have elucidated the role of the lipid bilayer to orient and facilitate molecules to bind their lipid-exposed target sites.^47^ Therefore, the protein–lipid interface can be considered as a target site in view of new strategies for drug design.^48^

In the present study, potential allosteric druggable sites of both the monomer and homodimer crystal structures of CXCR4 are identified using computational approaches that reveal regions of the protein that may involve allosterism and specific putative sites on the protein that may be amenable to the binding of drug-like molecules. The combined approach is appropriate for both sequestered and lipid-exposed sites. We employ the Gaussian network model (GNM)^49,50^ and a residue interaction network (RIN)^51^ model to discover potential allosteric residues that may regulate protein function. GNM can reveal the global and local motions of proteins using the two ends of the vibrational frequency spectrum, the lowest and highest frequencies, respectively. Haliloglu et al. developed the idea and showed in numerous studies that residues with high-frequency fluctuations of GNM located at hinge regions coordinating the global dynamics of the proteins correspond to known binding sites of ligands,^52,53^ such as DNA,^54^ antibiotics,^55^ as well as hotspots with a role in allosteric signaling.^56,57^ RIN can indicate allosteric residues/regions of a protein complex based on the contact topology summarized as a bidirectional graph.^58,59^ Accordingly, residues suggested by both the GNM and RIN can be plausible allosteric hotspots. In addition, a ligand binding in these regions is prone to induce conformational changes in the protein structure, as previously suggested for different protein complexes.^51,55,60,61^

To identify binding sites not evident based on crystallographic or cryo-EM structures alone, we utilized the site identification by ligand competitive saturation (SILCS) method. SILCS is a cosolvent molecular simulation approach that combines grand canonical Monte Carlo (GCMC) and molecular dynamics (MD) simulations to map the functional group affinity pattern of a protein, termed FragMaps, including contributions from protein flexibility and desolvation effects along with protein-functional group interactions.^62−68^ The FragMaps in combination with SILCS-MC docking of chemical fragments is capable of identifying putative druggable sites on a protein, a method termed SILCS-Hotspots.^69^ Here, we identified potential allosteric drug-binding sites mainly located in protein–lipid and protein–protein interfaces using GNM, RIN, and SILCS. Following the identification of the putative sites, we performed a virtual screening of FDA-approved drugs with SILCS-MC docking as well as Glide of Maestro (Schrödinger, Inc.). From the screens, high-scoring compounds possibly targeting CXCR4, such as those in the treatment of HIV-1 infection, were noted. The selected compounds were subjected to 50 ns MD simulations from which the binding free energies of the hit compounds were calculated using the molecular mechanics/Generalized Born Solvent Accessibility (MM/GBSA) approach as well as subjected to binding site affinity predictions based on SILCS Ligand Grid Free Energies (LGFE). Twenty compounds having predicted affinities to different allosteric sites were then tested with *in vitro* assays, and their ability to inhibit or enhance the binding of mAB 12G5 and/or cytokine CXCL12 was demonstrated. Assessed by the *in vitro* findings, these results showed the utility of the combined and efficient computational approach for the identification of putative allosteric modulator binding sites and potential ligands that may bind those sites.

## Methods

Gaussian network model (GNM)^49^ and residue interaction networks (RIN)^51,58^ were applied to the CXCR4 monomer and homodimer to reveal hub residues with a high potential to participate in the allosteric communication. The druggability potential of the pockets accommodating hub residues was further assessed with the SILCS approach^62−64^ including SILCS-Hotspots.^69^ Virtual screening was then performed targeting the potential allosteric sites on the monomer and homodimer structures using Glide standard precision (SP) docking^70−72^ and a library of 9800 compounds from the ZINC15 database. Secondary ranking of selected compounds was then performed using all-atom MD simulations coupled with MM/GBSA methods and SILCS through the SILCS-MC docking method to estimate the binding affinities based on the LGFE scores. A second virtual screening campaign with SILCS-MC docking was performed with an FDA-approved drug library of ∼350 compounds. Mutual drugs and derivatives from both campaigns were considered for further experimental studies.

### Protein and Database Preparation

The crystal structure of CXCR4 homodimer at 2.5 Å resolution was retrieved from Protein Data Bank with PDB ID: 3ODU.^73^ First, ligands, water molecules, and lysozyme subunits were removed from the structure. Subunit A was selected to study the monomer form. In both monomer and homodimer, N-termini (residues 27–33) and C-termini (residues 304–328) were truncated before the GNM calculations to prevent the “tip effect” that is likely to dominate the slow motions. In addition to the crystal structure, we also considered 20 conformers of the monomer and homodimer for RIN calculations. Protein conformers were randomly selected from the SILCS MD simulations. Root-mean-squared deviation (RMSD) values of the conformers were compared to the crystal structure showing conformational changes of up to 2.36 and 2.48 Å for monomer and homodimer, respectively, based on the protein nonhydrogen atoms.

Two virtual libraries of ligands were used for docking. A library of 9800 compounds consisting of FDA-approved drugs and investigational compounds from the ZINC15^74^ database were used for docking with Glide. In addition, a second virtual library of ∼350 FDA-approved drugs selected from the full set of FDA-approved compounds based on chemical diversity was used for the SILCS docking calculations.

### Gaussian Network Model

GNM describes the protein structure as a three-dimensional network of nodes connected by elastic springs. Nodes are located at the C_α_ atoms of the amino acids, and springs are the uniform forces among the neighboring amino acids.^49^ The potential energy of the elastic network (*V*_GNM_) is defined as the summation of pairwise harmonic interactions among the node pairs as,
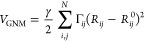
1where γ is the uniform spring constant, *R^0^*_*ij*_ is the equilibrium distance between *i*^th^ and *j*^th^ nodes (1 ≤ *i*,*j* ≤ *N*), and Γ_*ij*_ is the *ij*^th^ element of the Kirchhoff matrix Γ (*N* × *N*), which includes connectivity information on the nodes. The cutoff distance to determine the neighboring C_α_ – C_α_ pairs was taken as 10 Å.

*N*-1 vibrational modes were calculated by the eigenvalue decomposition of the Kirchhoff matrix as UΛU^T^, where the orthogonal matrix U contains the eigenvectors and the diagonal matrix Λ the eigenvalues. T is the transpose. One mode has a zero eigenvalue or vibrational frequency, which indicates rigid body motion. The lowest frequency modes correspond to globular motions of the protein, i.e., slow motions, such as hinge motions often related to biological functions. The highest-frequency motions, i.e., fast motions, are especially localized on residues that can alter the conformational energy landscape upon binding of a ligand, such as a small molecule, a protein, or DNA.^54,56,75^

The dynamic domains of a protein structure moving around hinge regions at the low-frequency motions can be revealed using the cross-correlation between fluctuations of residues *i* and *j*, Δ*R_i_* and Δ*R_j_*, calculated as
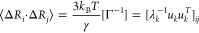
2where *T* is the temperature, *k*_B_ is the Boltzmann constant, *u*_*k*_ is the *k*^th^ eigenvector of U, and λ_*k*_ is the *k*^th^ eigenvalue of Λ. The cross-correlations can be calculated for each slow mode, while they can be averaged over a specific number of normal modes to determine the contribution of a group of modes to the collective dynamics.

High-fluctuating residues contributing to fast modes can be determined with the calculation of the relative fluctuations of *i* – *j* residue pairs as
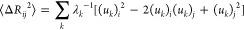
3

We considered the five slowest modes of the CXCR4 monomer and homodimer structures to detect their dynamic domains and hinge regions contributing to their global motions. These normal modes usually explain a considerable portion of the functional dynamics of a protein. In addition, the high-frequency fluctuating residues were determined using the 10 fastest modes. Similar to our previous studies on the ribosome^55^ and SARS-CoV-2 main protease,^60^ the high-fluctuating residues at the hinge regions were suggested as potential drug-binding sites for CXCR4 structures.

### Residue Interaction Network Model

RIN considers the protein structure as a network consisting of nodes linked by edges. Similar to the GNM, nodes are placed at C_α_ atoms of the residues. The lengths of the edges are determined based on the local interaction strength *a*_*ij*_ between neighboring residue pairs (*i*, *j*) that can be calculated as follows:
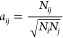
4where *N*_*ij*_ is the total number of the nonhydrogen atom–atom contacts of the *i*^th^ and *j*^th^ residues within a cutoff distance of 4.5 Å. To eliminate the effect of the amino acid size on the local interaction strength, *N*_*ij*_ is weighted by *N*_*i*_ and *N*_*j*_, being the number of nonhydrogen atoms of *i*^th^ and *j*^th^ residues, respectively. In this model, the close neighboring residue (node) pairs are considered to have strong interactions and can share information such as in the form of a fluctuation. The edge length between nodes *i* and *j* is calculated by 1/*a*_*ij*_, where the strong bias toward the covalent interactions is reduced.^51^

RIN strongly relies on protein topology. In this line, centrality measurements are highly beneficial in revealing the topological features of the protein network. Here, we used betweenness centrality (*C*_B_) for determining hub residues with a high potential to receive and send information through tertiary interactions so as to form allosteric communication paths between distant sites. *C*_B_ centrality is calculated as follows:
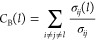
5where σ_*ij*_ is the shortest number of routes between nodes *i* and *j*, and σ_*ij*_(*l*) is the shortest number of routes between nodes *i* and *j* passing through node *l*. The regions containing hub residues with high betweenness values (top 5% of *C*_B_) were proposed as allosteric sites that can be evaluated as drug target sites.^51^

### Allosteric Site Prediction and Virtual Screening Protocols with SILCS

SILCS oscillating chemical potential, μ_ex_ GCMC/MD simulations were performed for the monomer and homodimer CXCR4 separately using the SILCS software suite (SilcsBio LLC). SILCS simulation protocols have been explained in detail previously,^68,76,77^ with the present study using the SILCS-Membrane protocol.^77^ All proteins were initially prepared using the CHARMM-GUI.^78^ The protein structures were then inserted in a membrane composed of POPC and cholesterol at a 90/10 ratio followed by a 6-step pre-equilibration protocol and a 10 ns MD simulation to relax the protein in the lipid bilayer.^78^ Using the protein and membranes from the equilibrated systems, the χ1 dihedral of the side chains of residues exposed to solvent by more than 0.5 Å^2^ were rotated by 36° increments yielding 10 initial starting structures for the monomer and homodimer systems for the SILCS simulations. The systems were then overlaid with solutes representative of common chemical functional groups (benzene, propane, methanol, formamide, dimethyl ether, imidazole, acetate, and methylammonium) corresponding to ∼0.25 M concentration along with water at ∼55 M using GROMACS.^79^ This is followed by equilibration of the solutes and water in the vicinity of the GPCRs using 25 cycles of GCMC as previously described. These systems were then subjected to 100 cycles of 200,000 GCMC steps of the water and solutes, a 5000-step steepest descent minimization and a 100 ps MD equilibration, and then a 1 ns production MD simulation of the entire systems. Harmonic restraints with a force constant of 0.12 kcal/mol/Å^2^ were applied on the C_α_ atoms throughout the MD simulations to prevent extreme conformational changes. Each CXCR4 structure was subjected to a total of 1 μs of production MD simulations (100 ns ×10) using GROMACS. The CHARMM36m^80^ force field parameters were used for the proteins, CHARMM36 used for lipids,^81^ along with the CHARMM General Force Field (CGenFF)^82^ for the solute parametrization and the CHARMM TIP3P water model.^83^

Snapshots from MD trajectories taken every 10 ps were used to calculate the SILCS FragMaps, which are based on probability distributions of selected solute atom coordinates divided into 1 Å^3^ grid elements. The normalized probability distributions were calculated using the solute concentration depending on the relative number of solute molecules to water molecules, assuming a concentration of 55 M water. Grid free energies (GFE) were calculated by converting the normalized probability distributions with the Boltzmann transformation. GFE FragMaps were associated with “generic” maps concerning apolar (C atoms of benzene and propane), hydrogen-bond donor (N atoms of formamide and imidazole protonated), hydrogen-bond acceptor (O atoms of formamide and dimethyl ether, N atoms of imidazole unprotonated), heterocycles (C atoms of imidazole), alcohols (O atom in methanol), positive donor (N atom in methylammonium), and negative acceptor (acetate carbonyl C atom). Using the GFE FragMaps allows for estimates of ligand binding affinities based on the overlap of selected ligand atoms with the maps from which GFE energies are assigned to the respective atoms with those GFE energies summed to give the ligand grid free energy (LGFE).

SILCS-Hotspots was used for identifying and characterizing putative allosteric binding sites.^65−68^ The approach is based on the SILCS-MC docking protocol in which translation, rotational, and dihedral degrees of freedom of ligands are subjected to MC sampling using the LGFE plus the CGenFF intramolecular energy with a 4r distance-dependent dielectric constant for the electrostatic interactions as the Metropolis criteria, as previously described.^69^ In SILCS-Hotspots SILCS-MC docking of chemical fragment molecules is conducted on the entire simulation box partition into 10 Å^3^ subvolumes with each fragment docked 1000 times in each subspace. Fragments used for hotspot identification included mono- and bicyclic compounds found in drug molecules.^84^ The 1000 docked orientations of each fragment were then subjected to root-mean-square distance (RMSD) clustering followed by a second round of clustering over all of the fragment types from which Hotspots were identified that contain one or more fragment types. Ranking of the SILCS-Hotspots used the average LGFE scores of all of the fragments in each site.

In addition, SILCS-MC docking was employed to screen a library of ∼350 FDA-approved drugs against multiple target sites on the CXCR4 monomer and homodimer. For each case, the docking region with a 5 Å simulation radius was centered on the hotspot containing the largest number of ring fragments. Top 20 best scoring ligands according to LGFE were collected for the investigated regions, while checking the ligand efficiency (LE, LGFE divided by the number of heavy atoms of the ligand) and the relative solvent accessibility (solvent-accessible surface area in the presence or absence of the protein, rSASA, 100% indicates that the ligand is fully excluded from the solvent in the ligand–protein complex) of the docked ligands.

### Structure-Based Virtual Screening and Molecular Dynamics Simulations with Maestro

Different protein conformers with RMSD values in a range of 2.0–2.7 Å were selected from the SILCS simulations for docking studies in Glide-Maestro. All structures were prepared using the OPLS4^85^ force field at pH 7.4 with the ProteinPrep tool in Schrödinger.^86,87^ Pharmacophore modeling was performed for the potential allosteric sites and the orthosteric site using the Pharmacophore Hypothesis module of Schrödinger,^88,89^ which takes the chemical and geometrical features of the residues into account. Finally, grid boxes with the approximate sizes of 23 Å × 23 Å × 23 Å were prepared with the Receptor Grid Generation module of Glide.^70−72^

For the virtual screening studies, a library of 9800 compounds downloaded from the ZINC15 database^74^ was employed, which comprised FDA-approved and investigational drugs. The compounds were screened against the pharmacophore model while seeking at least 3 matches out of 7 pharmacophore groups. LigPrep module of Schrödinger^87^ was used to assign the ionization states of the compounds under the physiological condition at pH 7.4. The compounds were docked to the potential allosteric binding sites using the Glide standard precision (SP)^70−72,87^ docking algorithm with default settings.

We considered the top 150 compounds having the best Glide binding scores, corresponding to the most favorable ∼30% binding scores. A total of 750 ligand–protein complexes for the monomer and homodimer structures were obtained in this step. We then calculated the Prime MM/GBSA single-point energies of the top 150 compounds docked to the site by using the Prime module of Schrödinger.

The Prime implicit membrane model of Schrödinger-Maestro and full-atom energy minimization were applied to calculate the Prime MM/GBSA energies of the ligand, protein, and ligand–protein complex as
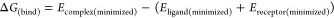
6where each *E* term consists of *E*_electrostatics_*=* (*H*_bond_ + *E*_coulomb_ + *E*_GB_solvation_), *E*_vdW_*=* (*E*_vdW_ + *E*_π–π_, + *E*_self-contact_), and *E*_lipophilic_. Solute conformational entropy was excluded from MM/GBSA calculations, resulting in relatively high binding free energy values. Compounds were selected with Prime MM/GBSA binding energies within ∼30 kcal/mol of the most favorable binding energy and with clinical relevance to the functional roles of CXCR4.

Selected compounds were subjected to 50 ns all-atom molecular dynamics (MD) simulations using OPLS4 force field^85^ with Desmond.^90,91^ Docked compound–protein complexes were placed in a POPC lipid bilayer. Explicit TIP3P water^83^ was used with a minimum 10 Å thickness from the protein surface. All systems were neutralized, and MD simulations were run under 0.15 M NaCl and pH 7.4. Nose–Hoover^92^ thermostat and Martyna–Tobias–Klein^93^ barostat kept the temperature (310 K) and pressure (1.013 bar) constant, respectively. Particle mesh Ewald^94^ was used with a 9.0 Å cutoff for long-range electrostatic interactions. Two fs time-step was set for the MD simulations with the SHAKE algorithm.^95^ Energy minimization and default membrane relaxation protocols in Desmond^90^ were applied to all systems as in the following:

(i) Brownian NVT dynamics at 10 K for 50 ps, while restraining solute heavy atoms with a force constant 50.0 kcal mol^–1^ Å^–1^.
(ii) Brownian NPT dynamics at 100 K for 20 ps using H_2_O barrier, while the membrane was restrained in the *z*-direction only with a force constant of 5.0 kcal mol^–1^ Å^–1^, and the protein complex restrained with a force constant of 20.0 kcal mol^–1^ Å^–1^.
(iii) NPT at 100 K for 100 ps using H_2_O barrier, while the membrane was restrained in the *z*-direction only with a force constant of 2.0 kcal mol^–1^ Å^–1^, and the protein complex was restrained with a force constant of 10.0 kcal mol^–1^ Å^–1^.
(iv) NPT heating from 100 to 300 K, for 150 ps using H_2_O barrier with weaker restraints (force constants on the membrane and the protein were 2.0 and 10.0 kcal mol^–1^ Å^–1^, respectively).
(v) NVT production at 300 K for 50 ps, while relaxing the membrane restraints and H_2_O barrier and a force constant on the protein is 5.0 kcal mol^–1^ Å^–1^.
(vi) NVT production at 310 K for 50 ps while removing all restraints and barriers.

NPT production was carried out at 310 K for 50 ns for each compound–protein complex to monitor the extent of interactions, especially with the hub residues, and to confirm the predicted binding sites. The stability of the compound–protein complexes was monitored by RMSD calculations using
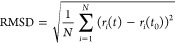
7Here, *N* is the number of atoms, *t*_0_ is the initial time when the first frame is recorded, and *r* is the position of the selected atoms. RMSD values of the ligands were calculated based on the ligand alignment on the protein backbone atoms of the first frame and the displacement of the heavy atoms belonging to the ligand. When the ligand RMSD was notably higher than the protein RMSD, the ligand possibly diffused away from its initial binding site.

Postsimulation MM/GBSA calculations based on 50 ns MD simulations were done with “thermal_mmgbsa.py”^96,97^ python script provided by Schrödinger. For each simulation, 1000 frames were produced, and MM/GBSA values were calculated based on production frames saved every 250 ps. Postsimulation MM/GBSA energies consist of Coulomb, covalent binding, van der Waals, lipophilic, generalized Born electrostatic solvation, and hydrogen-bonding terms.

### Final Hit Compound Selection

Top-scoring ligands suggested by SILCS-MC docking and Maestro-Glide for all potential allosteric and orthosteric sites were collected and ranked according to their LGFE and LE values based on the SILCS-MC docking calculations. The compounds were grouped according to their therapeutic purpose, such as antiviral, anticancer, antifungal, antimalarial, *etc*. The structures of compounds in each group from both approaches were visually checked to determine mutual compounds, derivatives or clinical usages, while the compounds with favorable LGFE and LE values for the orthosteric site were omitted. The final compounds were suggested as promising to potentially target CXCR4.

### Experimental Assay of Hit Compound Binding to CXCR4

Using a flow-based assay, the hit compounds (Selleckchem.com) were tested for their ability to inhibit the binding of either CXCL12 (SDF-a) or anti-CXCR4 (Clone 12G5). CXCR4+Cf2Th cells (NIH AIDS Reagent Program) were maintained in Complete Medium and detached prior to running the assay. Approximately, 2.5 × 10^5^ to 5 × 10^5^ cells in 200 μL were added to each well of a 96-well, v-bottom tray. The tray was centrifuged for 3 min at 2000 rpm. The supernatant was removed, and 100 μL of the compounds were added in triplicate to the tray at a concentration of 10 μM/test (well). The tray was incubated at 4 °C for 30–45 min. After the incubation, 100 μL of either biotinylated CXCL12 (SDF-1a) at a concentration of 3 μg/mL or 100 μL of anti-CXCR4 (6.7 μL/test) were added to the tray. The tray was incubated again at 4 °C for 30–45 min. Following the incubation, the trays were centrifuged for 3 min at 2000 rpm and the supernatants were removed. To the CXCL12 tray, 100 μL (6.7 μL/test) of Streptavidin PE was added, and the tray was incubated at 4 °C for 30–45 min. To the anti-CXCR4 tray, 200 μL of wash/stain buffer was added, and the tray was centrifuged for 3 min at 2000 rpm. The supernatant was removed, and the wells were resuspended in 1–2% paraformaldehyde. After incubation with Streptavidin PE, the CXCL12 tray was similarly centrifuged and washed prior to the addition of 1–2% paraformaldehyde. The assay was run on an LSR Fortessa II instrument and further analyzed in FlowJo (FlowJo) and Prism (GraphPad). The readout was MFI (mean fluorescence intensity).

### Functional Screening of Hit Compounds

We screened the hit compounds for agonist and antagonist activities using a commercially available system (Tango CXCR4-*bla* U2OS Cells, ThermoFisher Scientific) that is based on the observation that β-arrestins are critical in agonist-induced internalization of GPCR receptors.^98,99^ The Tango CXCR4-*bla* assay uses the U2OS transformed epithelial cell line expressing CXCR4 fused to a Ga14-VP16 transcription factor via a TEV protease sequence from the Tobacco Etch Virus. The CXCR4-*bla* U2OS cells also express β-arrestin fused with the TEV protease and a β-lactamase (bla) reporter construct under the control of a UAS element that responds transcriptionally to free Ga14-VP16 when it is cleaved from CXCR4. When CXCL12 or another agonist is added, the β-arrestin-TEV protease chimeric protein is recruited to the cytoplasmic tail of CXCR4 where it cleaves Ga14-VP16 that subsequently activates bla-expression via the UAS response element. Activity is quantified using a bla substrate that has two fluors, coumarin and fluorescein, in a configuration that enables fluorescence resonance energy transfer from coumarin to fluorescein, resulting in a green signal when excited at 409 nm. This FRET signal is lost when the substrate is cleaved by bla resulting in a blue signal at 447 nm. The coumarin:fluorescence ratio is used as a normalized response to CXCL12. This assay format is frequently used in high-throughput studies to probe GPCR agonism and antagonism by libraries such as our hit compounds.^100−106^

## Results and Discussion

CXCR4 is frequently overexpressed in cancer cells^107^ and at oncogenic expression levels, CXCR4 is predominantly in dimer form.^13^ The dimerization interface was previously reported to take part in allosteric communications between two protomers.^108^ Accordingly, we investigated both the monomeric and dimeric forms of CXCR4 and followed a systematic computational approach to reveal potential allosteric pockets and suggest hit compounds for CXCR4. First, potential allosteric sites on the monomer and homodimer structures were determined using the Gaussian network model (GNM) and residue interaction networks (RIN) approaches. The selected sites were then evaluated with SILCS-Hotspots for their druggability. Multiple distinct druggable pockets were proposed for the monomer and the homodimer. We employed two structure-based virtual screening and docking softwares, Glide-Maestro and SILCS-MC, using different protocols to determine potential compounds to target putative allosteric pockets of CXCR4 in its monomer and dimer forms. The selection of the hit compounds from all screens was mainly based on their therapeutic purpose, i.e., a potential activity through CXCR4 that is related to different diseases. We discuss our findings in this order to propose potential allosteric binding sites and associated compounds for the modulation of CXCR4 activity. Finally, the activities of 20 hit compounds were tested with *in vitro* binding and functional assays.

### Prediction of Allosteric Residues by GNM and RIN

GNM and RIN were used together to determine plausible allosteric drug-binding sites on the CXCR4 monomer and homodimer structures. The high-frequency fluctuating residues located at the hinge regions that coordinate the globular motions of the protein are attractive regions for drug targeting.^55^ If these residues also have a high ability to transmit a perturbation in the form of conformational changes, which can be revealed by RIN,^58,59^ they can be proposed as allosteric drug target regions.

**Figure 1:**
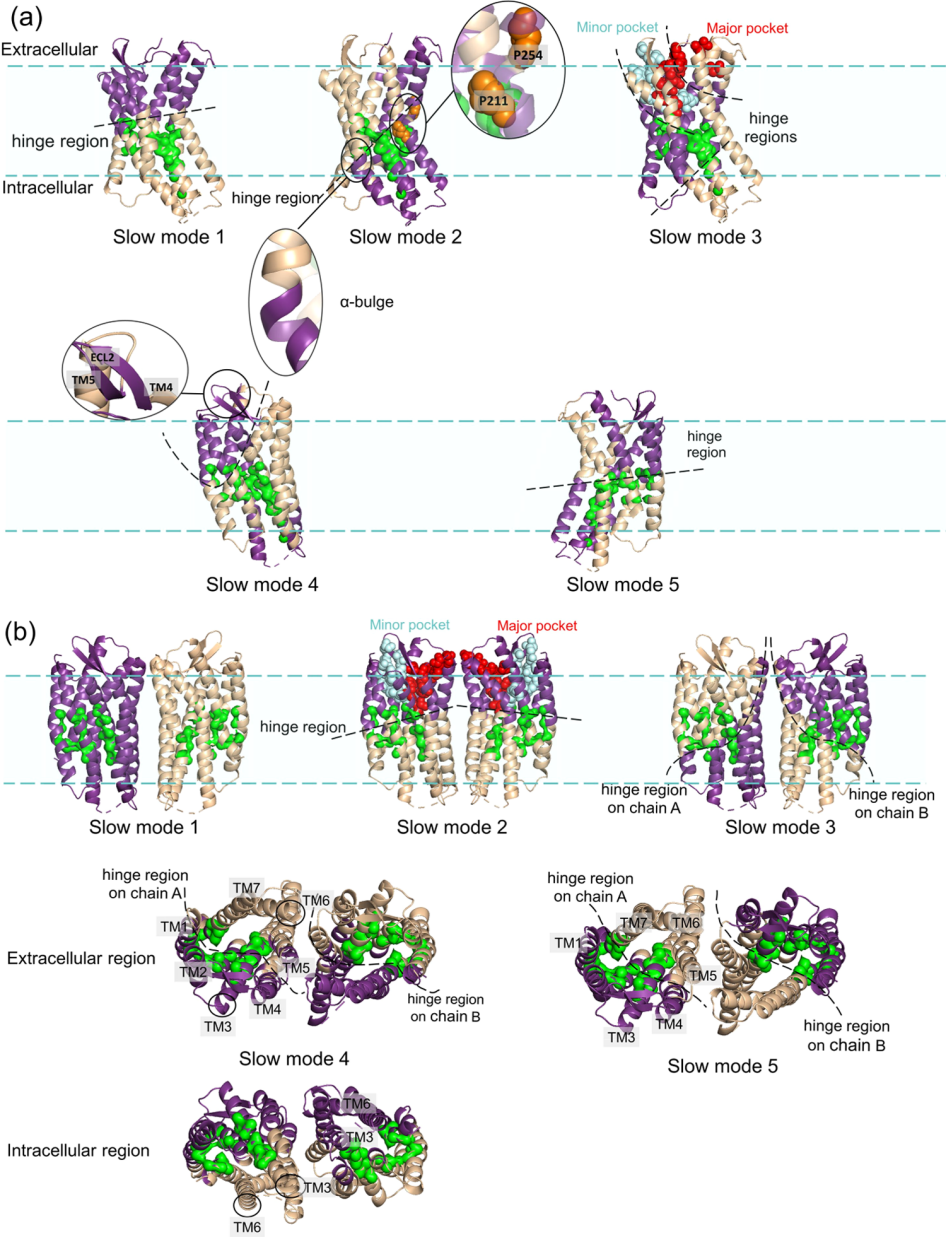
Low-frequency global motions (dynamic domains colored in wheat and purple) and the high-frequency local motions in the 10 fastest modes (shown as green surface) of CXCR4 (a) monomer and (b) homodimer predicted by GNM. The orthosteric site consists of major and minor binding pockets, shown in red and cyan, respectively.

Figure 1 displays the hinge regions coordinating the low-frequency motions of the dynamic domains on the monomer and homodimer structures, determined by GNM. We considered the first five slowest modes in the analysis, which correspond to 19 and 26% of the overall motion of the monomer and homodimer, respectively. Different colors on the protein structures indicate distinct dynamic domains moving in anticorrelated directions in a specific slow mode. The residues on the hinge regions of the five slowest modes are given in Table S1. In the first slow mode of the monomer (7% of the overall dynamics), the hinge region residues divide the structure symmetrically parallel to the transmembrane domain (Figure 1a). In its second slowest mode, the hinge region highlights the α-bulge that can be effective in the dimerization of GPCR.^109^ The global domains become distinct and introduce the kink due to P211 on TM5 and P254 on TM6 residues because of hydrogen-bond (H-bond) disruption.^110^ Furthermore, orthosteric site residues (H113, T117, D171, R188, and E288), which participate in major or minor binding pockets of CXCR4,^73,111^ are noted as the hinge region residues of the dynamic domains of the third slow mode of CXCR4 monomer. The fourth slowest mode comprises the anticorrelated motions of extracellular loop 2 (ECL2), which has a critical role in ligand binding.^112,113^

For the homodimer, the first slow mode (17% of the overall dynamics) indicates that chains A and B are anticorrelated to move as distinct dynamic domains (Figure 1b). Its second slow mode is similar to the first slow mode of the monomer. In the third mode, the hinge regions divide the transmembrane helices into two dynamic domains in each chain. The motion of TM6 is anticorrelated against TM1 and TM2 in the fourth slowest mode of the homodimer. This mode seems to correspond to a transition from active to inactive state in GPCR structures indicated by the outward motion of TM6.^114,115^ Furthermore, in the fourth and fifth slowest modes, the anticorrelated motion of helix 3 against helix 6 on the transmembrane is noted, but they move in a correlated fashion in the intracellular region. This plausibly corresponds to an active-like state of CXCR4, where TM6 becomes distant from TM3, as was previously indicated with MD simulations.^25^

We observed that residues with high-frequency fluctuations accumulate at the hinge regions determined from low-frequency motions. The list of these residues is given in Table S1. Among these, residues with critical roles in CXCR4 function were noted, such as W94, which is highly conserved among chemokine receptors and has a crucial role in ligand binding.^116^ Y116 and E288 are known to be responsible for signal initiation throughout CXCR4 upon ligand binding.^116^ In addition, L244, I245, F248, W252, and A291 at the hinge regions take part in CXCR4 signal propagation for G-protein activation in the intracellular region.^116^ F248, reported as a critical residue among point mutations of CXCR4, shows strong reactivity against conformationally sensitive monoclonal antibodies.^116^ The microswitch residue, S131, which controls G-protein coupling, has high-frequency distance fluctuations as well. Fast modes also point to other residues such as G55, V88, and F292, of which mutations are critical for ligand binding and receptor signaling.^116−118^

**Figure 2:**
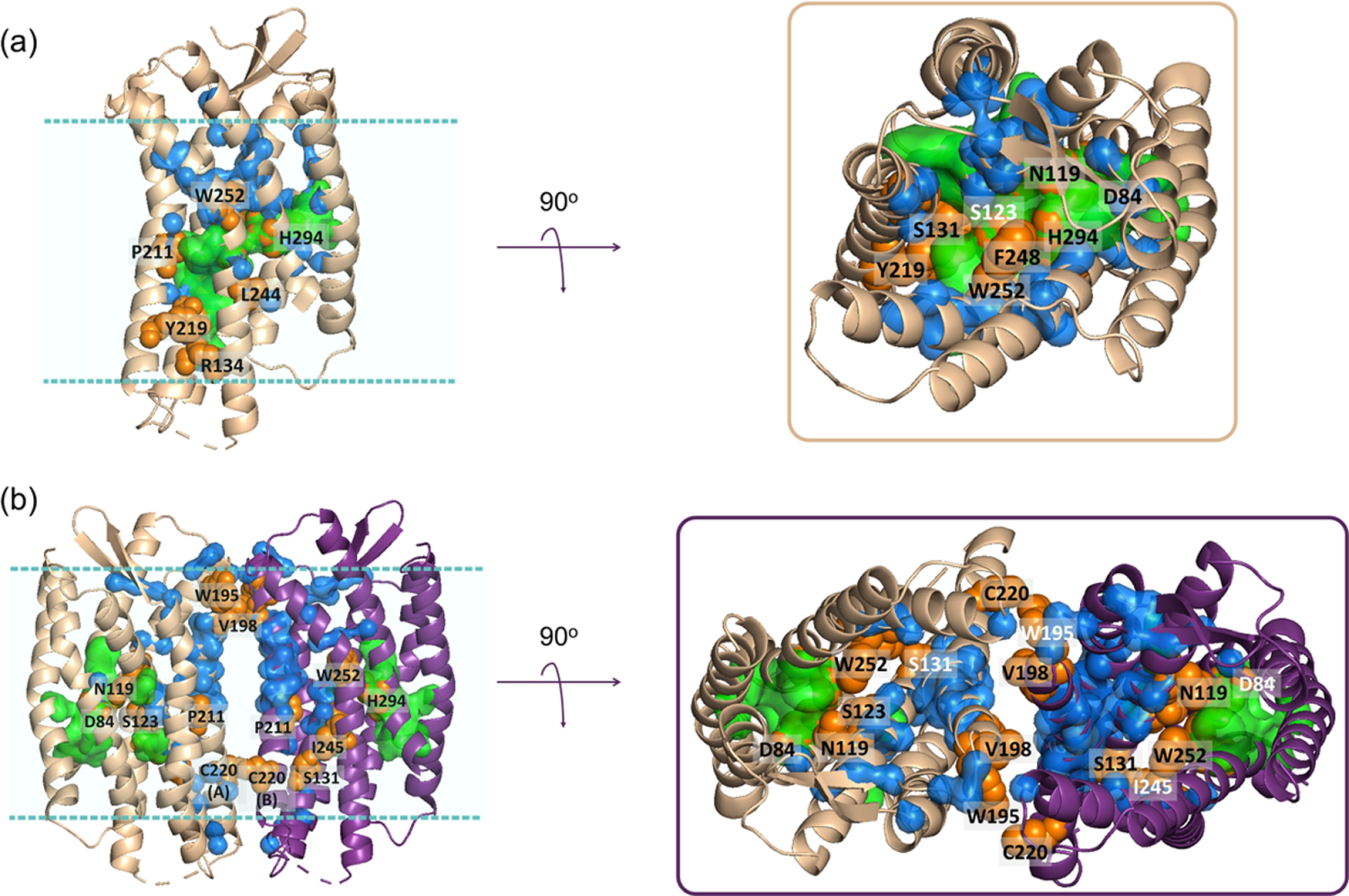
Hub residues with high betweenness predicted by RIN for monomer (a) and homodimer (b) are shown in blue. High-frequency fluctuating residues at the hinge regions determined with GNM are in green, and residues with known critical functions are colored in orange.

Next, the CXCR4 monomer and homodimer structures, as well as conformers obtained from the SILCS simulations, were investigated with RIN, and the hub residues with a high potential to participate in allosteric communications among distant sites were determined. Here, the residues with high betweenness scores in the top 0.05 quantile were considered (listed in Table S1), as in the previous studies.^51,60,61^ The topology of the protein may change during its functional dynamics; new hub residues with high betweenness may be determined based on different conformers. Accordingly, we identified the residues with high betweenness scores from all investigated structures for both monomer and homodimer CXCR4. Figure 2a displays the hub residues with high betweenness values together with the residues having high-frequency distance fluctuations on the CXCR4 monomer. GNM and RIN are in good agreement on the residues with a high potential to participate in allosteric modulation. On the monomer CXCR4, TM3 accommodates numerous hub residues when compared to other transmembrane helices. Class A GPCR activation was reported to take place upon the displacement of TM6 outward from TM3.^25^ In some cases, GPCRs undergo significant structural changes at TM3 upon their activation with G-protein for downstream signaling.^119−121^ Moreover, the microswitch residue S131, is determined as a hub residue in both the monomer (Figure 2a) and homodimer CXCR4 (Figure 2b). Besides, another microswitch, Y219, which is assumed to be effective on the G-protein interface,^116^ resides in the hinge region. RIN also highlights P211 as a hub residue in both monomer and homodimer, which comes into play with the kink characterization of transmembrane proteins.^122^ The residues D84, N119, S123, and H294 have high betweenness values; these constitute the binding pocket of the allosteric modulator Na^+^ ion^25^ (Figure 2a). Hub residues L244, I245, and F248 on TM6 that are also hinge residues attend the “hydrophobic bridge” and regulate GPCR signal propagation.^116^

The critical residues determined by GNM and RIN also constitute highly conserved signaling motifs among the GPCRs. For example, R134 participates in the DRY motif, Y219 and C220 construct Y(x)_5_KL, and W252 on TM6 is a member of the CWxP rotamer motif.^116^ GNM and RIN both suggest that the dimer interface of CXCR4 has a considerable number of hub residues. Hub residues W195 and V198 contribute to dimer stabilization on the protomer interface.^73^ It is worth noting that this site was evaluated as an allosteric drug-binding site, particularly for cancer cell targeting.^4^

### Identification of Druggable Sites in the Allosteric Predicted Regions with SILCS-Hotspots

To identify putative druggable sites in the potential allosteric regions determined with the GNM and RIN, we applied SILCS simulations in conjunction with SILCS-Hotspots. The SILCS approach provides a comprehensive mapping of possible fragment binding sites on the target protein, including in the protein interior. SILCS-Hotspots integrate extensive fragment screening in the field of FragMaps using SILCS-MC docking followed by fragment clustering. It thus identifies putative fragment binding sites, or Hotspots, and ranks them according to average LGFE scores or other user-selected metrics.^69^ However, as previously discussed, for the identification of binding sites suitable for drug-like molecules, sites in which adjacent Hotspots are present are preferred oversimply the most favorable Hotspots based on LGFE scores. This approach is highly beneficial for determining sites on the suggested allosteric regions that are druggable as well as for revealing pharmacophore features in these sites, facilitating the design of novel drugs.

**Figure 3:**
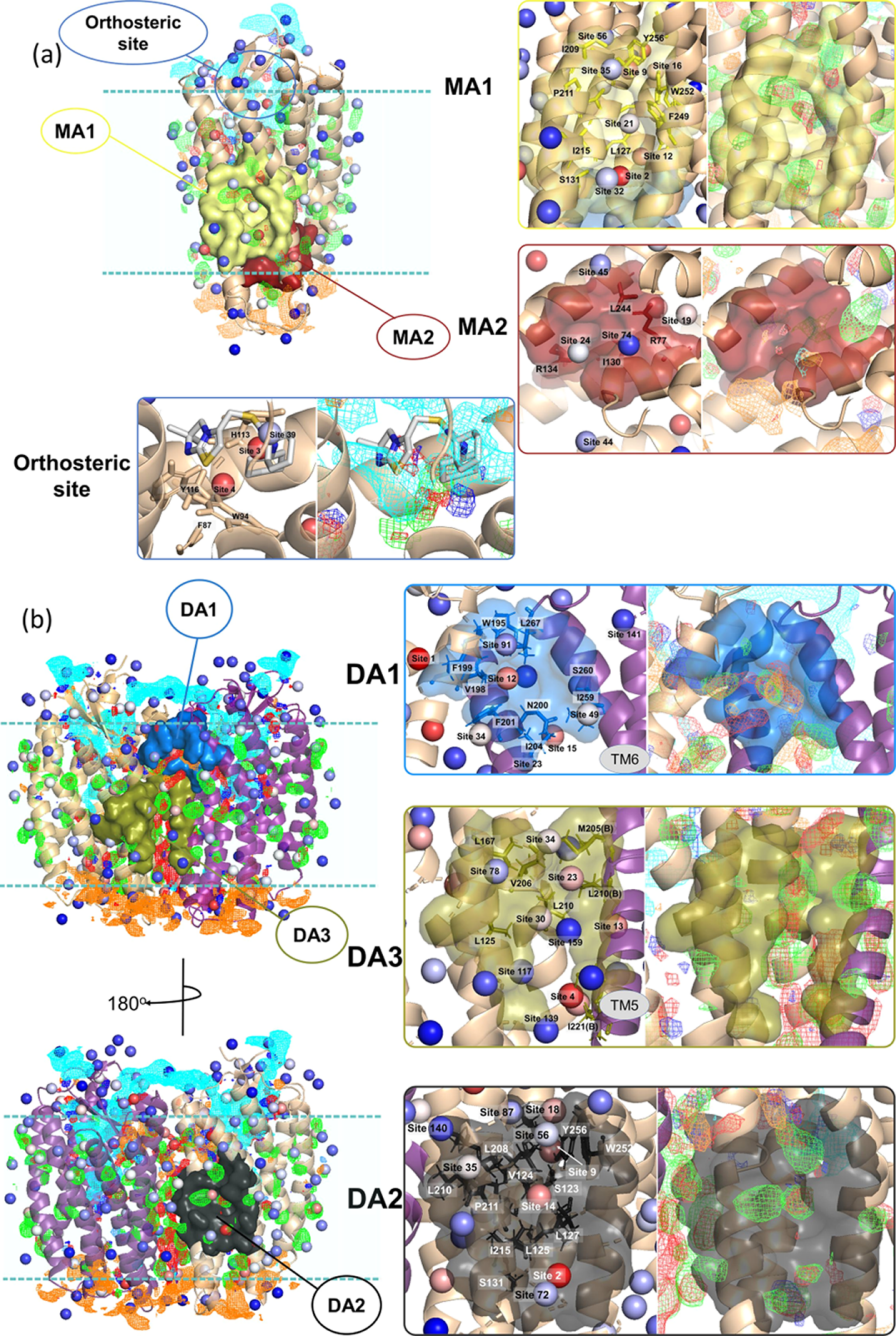
(a) Orthosteric site of CXCR4 and its inhibitor *1Tit*, potential allosteric sites for monomer, MA1, and MA2 and (b) potential allosteric sites for the homodimer CXCR4, DA1, DA2, and DA3. Both in (a) and (b), the Hotspots are shown in vdW spheres colored based on mean LGFE score (from red—most favorable to blue—least favorable); SILCS FragMaps are visualized as, positive (cyan, −1.2 kcal/mol), negative (orange, −1.2 kcal/mol), apolar (green, −1.2 kcal/mol), H-bond donor (blue, −0.9 kcal/mol), and H-bond acceptor (red, −0.9 kcal/mol).

Figure 3a illustrates the FragMaps and Hotspots of monomer CXCR4. Each Hotspot in SILCS represents the center of one cluster of fragments; binding sites for drug-like molecules are composed of two or more adjacent Hotspots. For all of the Hotspots, the mean LGFEs over all of the fragments in each site are ranked between −3.93 and −2.06 kcal/mol (Table S2). Figure 3a displays the orthosteric site (residues of minor subpocket W94, D97, W102, V112, H113, Y116, R183-R188, and E288), which is occupied by multiple Hotspots. These include two Hotspots with the third and fourth lowest mean LGFE values (−3.65 and −3.59 kcal/mol, respectively) that are surrounded by hub residues F87, T90, W94, H113, and Y116 predicted by GNM and RIN. CXCR4 antagonist IT1t^73^ overlaps with Hotspot 3 and is located 3.4 and 2.6 Å away from sites 4 and 39, respectively. Analysis of the FragMaps shows that positively charged functional groups will have favorable interactions where Hotspots 3 and 39 are located, while apolar, H-bond donor, and H-bond acceptor FragMaps are present around Hotspot 4.

Beyond the orthosteric site, the two regions on the monomer CXCR4 that include residues identified as having a potential allosteric role were analyzed to determine if potential allosteric sites are present based on the SILCS-Hotspots analysis (Figure 3a). In allosteric region #1 on the monomer, MA1, there are several adjacent Hotspots between TM helices 5 and 6 indicating a potential drug-like binding site. This site, which includes Hotspots 2, 9, 21, and 35 (mean LGFE ranging between −3.67 and −2.84 kcal/mol), is occupied by apolar and H-bond acceptor fragments. In the second allosteric region, MA2, there is a putative binding site occupied by Hotspots 24, 74, and 19 (mean LGFE ranging between −3.10 and −2.36 kcal/mol) along with negative FragMaps with an apolar FragMap around site 19. Small H-bond acceptors and positive FragMaps are also present. When deciding on the druggability of the site, we also considered the number of fragments engaged in the Hotspots at each site. The number of fragments of the MA1 Hotspots varies from 14 to 86, whereas the maximum number of fragments is 65 for MA2. Occupancy by a large number of fragments indicates that the site is suitable for binding different types of groups that would facilitate future drug design efforts.

We subsequently examined homodimer CXCR4 to identify potentially druggable pockets in the allosteric regions with SILCS-Hotspots. All findings are given in Table S3. Results from GNM, RIN, and SILCS calculations for the homodimer suggest three distinct sites (Figure 3b). For the potential allosteric region DA1, Hotspots 12 and 91 (mean LGFEs of −3.67 and −2.70 kcal/mol, respectively) are located in a pocket between TM5 and TM6. The site is occupied by up to 83 fragments that include negatively charged, H-bond acceptor, and apolar fragments. In the case of the potential allosteric site DA2 located on chain A, Hotspots 56, 14, and 2 are in a putative binding pocket adjacent to TM5 and TM6. The mean LGFE values are between −4.15 and −3.02 kcal/mol with the number of fragments up to 66. The site is occupied by FragMaps with apolar and H-bond acceptor chemical properties, and there are positive and negative FragMaps posing around the site. In the potential allosteric region DA3, which is located at the interface of chains A and B, there are a number of Hotspots including 34, 23, 30, 139, and 4 with the site occupied by apolar and H-bond acceptor FragMaps. Here, the maximum number of fragments is 66 belonging to Hotspot 30.

In summary, we revealed two potential allosteric regions that can be druggable on the CXCR4 monomer: MA1 occupied by central site 21 around residues L208, I215, and F249 in the transmembrane area; MA2 of which central Hotspot is 24, and composed of allosteric residues R77, I130, and R134, in the intracellular region. For the homodimer, three potential allosteric sites in the transmembrane area were determined; DA1, DA2, and DA3, of which most central hotspots are 12 (within 6.0 Å distance of F199, F201, and L267), 14 (around L208, I209, G212, and Y256) and 30 (around L125, V206, L210, and P211), respectively. The list of the residues is given in Table S4. These sites are plausibly critical for the function of the CXCR4 and, thus, further investigated with molecular docking and binding free energy calculations.

### Structure-Based Virtual Screening

Virtual screening studies were conducted, targeting the sites identified through the combination of findings from GNM, RIN, and SILCS-Hotspots. A library of 9800 FDA-approved drugs and investigational drugs retrieved from the ZINC15 database were screened using Maestro-Glide. Screening was performed against the crystal structure, and different conformers that were selected from the SILCS GCMC/MD simulations, using standard precision of Glide. The filtering procedure focused on high docking score and Prime MM/GBSA yielded a total of 41 compounds, including 12 for the monomer and 29 for the homodimer, as listed in Tables S5 and S6, respectively. These were subjected to full atomistic 50 ns long MD simulations to monitor the extent of interactions between the compounds and potential allosteric residues. MD simulations also gave insights into binding free energies of the compounds–protein complexes using the MM/GBSA approach. In addition, SILCS-MC docking was used to get additional estimates of the binding free energies for the 41 compounds found with Maestro-Glide.

The potential allosteric pocket MA1 of monomer is located in the region of the GPCR that defines the interface involved in homodimerization, which has been previously investigated using FTMap and docking calculations.^38^ 9 promising compounds for MA1 and 3 for MA2 were identified based on the docking scores and Prime MM/GBSA energies of Glide (Table S5). Binding free energy values based on MD simulations are in coherence with the docking scores, Prime MM/GBSA values, and SILCS-MC ligand grid free energy (LGFE) values, except with Fulvestrant, Relugolix, and investigational ZINC29238439. As also identified via the SILCS FragMaps, π–π stacking, π-cation, and H-bond interactions dominate the ligand–protein complexes in MA1. Focusing on the cavity, ligands often interact with the potential allosteric residues (Figures S1–S8). For MA2, the interactions between the docked ligands and the pocket mostly include H-bonds, polar contacts, and water bridges, also involving potential allosteric residues R134 and A237 (Figures S9–S12). For all cases, except for Fulvestrant (Figure S7) and Pimavanserin (Figure S9), the RMSD values of both the ligand and the protein are low, showing that the ligand–protein interactions are quite stable for the docking pose throughout the simulation. Fused rings of Fulvestrant are noted to remain in the binding cavity while the long tail is highly mobile while Pimavanserin loses the majority of its interactions that were present in the initial docking pose.

We propose three potential allosteric pockets (DA1, DA2, and DA3) to target homodimer CXCR4. After molecular docking and Prime MM/GBSA calculations in Maestro-Glide, 29 compounds were determined as promising allosteric ligands (7 for DA1, 7 for DA2, and 15 for DA3 given in Table S6). For DA1, the potential allosteric residues W195 on chain A and F201, S260, and L267 on chain B make favorable interactions with the compounds. Some compounds, such as Amelubant, change their conformation in the cavity as noted from fluctuating RMSD values (Figures S16–S18) and Ly377604 does not keep its initial docking pose in the cavity (Figure S19). The second potential allosteric pocket, DA2 is located on chain A. Seven compounds, including HIV protease inhibitors, anticancer, and antipsychotic agents, have relatively favorable docking scores, LGFE, and ligand efficiency (LE) values. The potential allosteric residues P211, I215, Y219, I245, F249, W252, and Y256 were noted to stabilize the compounds in the binding cavity (Figures S20–S26). The third potential allosteric pocket, DA3, is located at the interface of chains A and B. The promising compounds according to Maestro-Glide calculations are used to treat fungal infections, seasonal allergic rhinitis, asthma, malaria, heart failures, ovarian and breast cancer, HIV infection, and chronic psychoses. The docking scores as well as MM/GBSA values agreed with the LGFE and LE values. The potential allosteric residues were involved in favorable contacts with the docked ligands, which were maintained in the pocket during the MD simulations (Figures S27–S41). For all investigated cases, MD simulations confirmed the binding sites identified through GNM, RIN, and SILCS calculations.

The SILCS-Hotspots approach enables drug design by identifying multiple fragments associated with adjacent hotspots in targeted pockets that may be linked to create drug-like molecules. From this perspective, we observed a high similarity between the identified fragments and the compounds suggested by Maestro-Glide in both monomer and dimer calculations. For instance, high-scored Itraconazole (Figure S42) docked on DA3 contains a dioxane ring identical to fragment 70 at site 12. Also, fragment 56 at site 30 was consistent with the triazole derivative found on Itraconazole.

As the putative allosteric sites were further validated in terms of ligand-residue interactions during MD simulations, we performed a second screen against a more focused library of ∼350 FDA-approved compounds. In this second virtual screening campaign, SILCS-MC docking was carried out with the small drug library using the monomeric and homodimeric forms of CXCR4. Twenty compounds were determined for each investigated site based on favorable LGFE and LE, and relative solvent-accessible surface area (rSASA%) (Tables S7 and S8). Several high-scoring compounds were mutual to multiple allosteric sites, such as Landiolol hydrochloride (β1-superselective intravenous adrenergic antagonist), Maraviroc (antiretroviral targeting CXCR5), Darunavir (antiretroviral to treat HIV-1 infection), Dehydroandrographolide Succinate (antiviral), Delamanid (to treat tuberculosis), Empagliflozin (antidiabetic), Travoprost (to treat high ophthalmic pressure), and Terconazole (antifungal).

The quality of the predicted allosteric sites was further evaluated relative to the orthosteric site of CXCR4 using SILCS-MC docking (Table S9). As the orthosteric site is a true binding site, the LGFE and LE values for the ∼350 FDA-approved drug library can indicate whether the proposed allosteric sites were plausible binding sites. Calculated LGFE and LE values for the putative allosteric sites indicated more favorable ligand–receptor binding affinities when compared with the orthosteric site, suggesting that the proposed sites are plausible binding regions.

The high-scoring compounds from both screening campaigns for all putative allosteric sites and orthosteric sites were collected and ranked according to their LGFE and LE values. Compounds with favorable LGFE and LE for the orthosteric site or without any known therapeutic purpose were eliminated. The remaining compounds were then grouped according to their clinical usage, keeping in mind the critical role of CXCR4 in various diseases. High-scoring compounds or their derivatives with possible relation to CXCR4 were selected as hits to target either monomeric or dimeric CXCR4 as listed in Table 1. Here, some compounds from SILCS-MC and Glide had mutual structure/clinical usage, such as antifungals; Terconazole, Itraconazole, and Isavuconazonium, and antiretrovirals; Darunavir, Brecanavir, Tipranavir, and Ritonavir.

**Table 1:** 20 Hit Compounds Selected for *In Vitro* Assays

allosteric pocket	drug number	drug name	clinical usage
MA1, DA1, DA2, DA3	1	Darunavir	HIV protease inhibitor
MA2, DA2, DA3	2	Sofalcone	gastric mucosa protective agent
MA2, DA2, DA3	3	Delamanid	used in combination with other tuberculosis drugs for active multidrug-resistant tuberculosis
MA1, DA3	4	Terconazole	antifungal
MA1, DA1	5	Loganin	Iridoid glycoside, used as a neuroprotective and anti-inflammatory agent
MA1, DA3	6	Retapamulin	antibacterial agent against superficial skin infections caused by *Staphylococcus aureus*
DA2, DA3	7	Testosterone enathalate	androgen and anabolic steroids
MA1, MA2, DA1, DA2, DA3	8	Travoprost	used in the treatment of intraocular pressure related to open-angle glaucoma or ocular hypertension
MA1, DA1, DA2, DA3	9	Empagliflozin	selective inhibitor of sodium-glucose cotransporter 2
DA1, DA2, DA3	10	Dehydroandrographolide succinate	antiviral agent for the treatment of pneumonia and viral upper respiratory tract infections
MA1	11	Tribenzagan hydrochloride	antiemetic agent for the treatment of nausea and vomiting
MA1, DA1, DA2, DA3	12	Landiolol hydrochloride	β-blocker to treat cardiac arrhythmias
MA1, DA1, DA3	13	Maraviroc	CCR5 antagonist to treat HIV infection
MA1, DA1	14	Rosuvastatin calcium	statin drug to reduce the level of blood lipids
MA2, DA2, DA3	15	Pranlukast	leukotriene receptor antagonist against allergic rhinitis and asthma
DA1	16	Dipyridamole	to inhibit blood clotting
MA1	17	Topiramate	for the control of epilepsy attacks and in the prophylaxis and treatment of migraines
MA1, DA1, DA2, DA3	18	Mycophenolate mofetil	inosine monophosphate dehydrogenase inhibitor to prevent organ transplantation rejection
DA1	19	Capecitabine	chemotherapy agent for breast cancer, gastric cancer and colorectal cancer
MA1	20	Cilostazol	vasodilator

### Screening of the Hit Compounds by *In Vitro* Binding

We developed a flow-cytometry-based assay to screen the hit compounds for their ability to inhibit or enhance the binding of standard probe ligands to cell surface CXCR4. The compounds were CXCL12 (SDF-1a),^123,124^ the canonical CXCR4 agonist, or the well-characterized 12G5 monoclonal antibody (mAb)^125^ that binds to a conformational epitope in ECL2 of CXCR4.^125−128^ CXCL12 and 12G5 can compete for binding to CXCR4, but their binding surfaces on CXCR4 are not congruent. While both ligands bind ECL2 of CXCR4, 12G5 recognizes an epitope on CXCR4 via common residues distinct from those contacted by CXCL12.^126−129^ For example, CXCL12 binding to CXCR4 is enhanced by *O*-sulfation of tyrosine residues in the N-terminus of CXCR4.^129−131^ By contrast, this post-translational modification does not strongly affect the binding of 12G5.^126^ There is no atomic structure of CXCL12 binding to CXCR4, but a model combining an NMR structure of CXCR4 binding to residues 1–38 of CXCL12 and a structure of CXCR4 bound to a small molecule inhibitor, IT1t^73^ was reported for an intact CXCL12:CXCR4 complex.^126^ Mutations in CXCR4 outside CXCL12 contact residues abrogate 12G5 binding.^126^ Given the wide use of CXCL12 and 12G5 as functional and conformational probes, we developed a flow-cytometry-based assay for a preliminary screen of the ligand hit compounds to block or enhance the binding of CXCL12 and 12G5 to CXCR4 on the widely used canine cell line CXCR4+Cf2Th that expresses high levels of human CXCR4.^130^ The assay was performed as described in the Methods section using concentrations of CXCL12 or 12G5 determined in preliminary titration studies corresponding to approximately half-maximum binding of each ligand. This approach provides the most sensitive window to determine whether a ligand hit compound affects the binding of either ligand to CXCR4. The compounds were screened in triplicate at 10 μM diluted in culture media. No precipitation of the compounds was observed throughout the study.

**Figure 4:**
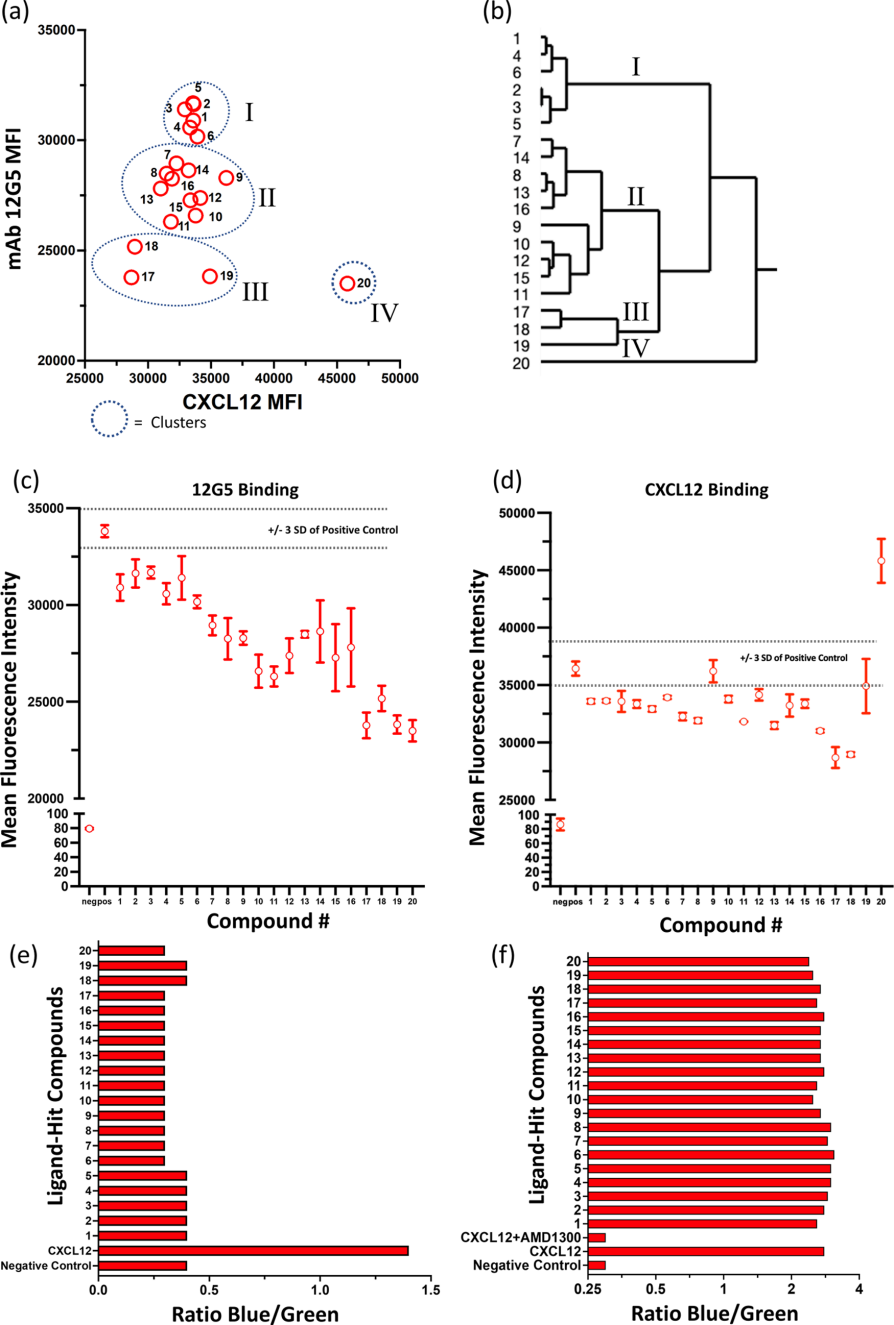
Identification of hit compound clusters by hierarchical clustering analysis. (a) Bivariate plot of the clustering data shown in the dendrogram in (b). The Roman numerals correspond to the clusters in panels (a) and (b). Effects of hit compounds on binding of (c) 12G5 and (d) CXCL12 to CXCR4 on the CXCR4+Cf2Th cell line. (e) Tango CXCR4-*bla* agonism assay using CXCL12 (10 nM) as the positive control. (f) Tango CXCR4-*bla* antagonism assay using CXCL12 (10 nM) as the positive control and CXCL12 (10 nM) plus AMD1300. The compound indices (1–20) are given in Table 1.

The binding data were analyzed for statistical significance using one-way analysis of variance (ANOVA) and pairwise multiple comparisons of each hit compound versus the positive control for 12G5 and CXCL12 (Table 10). There were statistically significant effects of the hit compounds for 12G5 and CXCL12 by ANOVA. Most pairwise comparisons for the hit compounds versus 12G5 were statistically significant, whereas the comparable pairwise comparisons for CXCL12 had fewer statistically significant differences. Based on this observation, hierarchical clustering further analyzed the data, where four unique clusters were apparent (Figure 4a,b).

The bivariate plot shown in Figure 4a shows four hit compound clusters that affect the binding of CXCL12 and 12G5 that were identified by hierarchical clustering in the dendrogram shown in Figure 4b. Figure 4c,d shows the effects of the hit compounds individually on binding CXCL12 and 12G5 to CXCR4 on CXCR4+Cf2Th cells, respectively. The hit compound clusters I, II, and III revealed successively decreasing CXCL12 and 12G5 binding levels, showing a concordant inhibition among the compounds in each cluster. In general, the variation in binding among the hit compounds was larger and for the top compounds stronger with respect to 12G5 versus CXCL12. Strikingly, the hit compound 20—Cilostazol in cluster IV ranked with cluster III for inhibition of 12G5 binding but was unique in that it enhanced CXCL12 binding.

To evaluate if the hit compounds are potentially interacting directly with the binding sites of the ligand CXCL12 and monoclonal antibody 12G5,^116,132^ we applied SILCS-MC docking to obtain the affinity of 20 hit compounds^107,123^ based on the LGFE scores. In the majority of cases, the predicted binding affinities were less favorable as compared to the investigated allosteric sites (Table S11) indicating the activity of the compounds to not be due to direct interactions with the CXCL12 and 12G5 binding sites. Collectively, these data are consistent with the predicted activity of the selected FDA hit compounds that impact the binding of CXCL12 and 12G5 to CXCR4 through binding to the putative allosteric binding sites. In addition, the present results indicate the potential for differential modulation of CXCL12 and 12G5 binding to CXCR4.

We then conducted a preliminary functional assay to determine the agonist and antagonist activities of the hit compounds. The hit compounds were screened using the Tango CXCR4-*bla* assay at a concentration of 10 μM, as used in the binding assay, to determine ligand-dependent β-arrestin2 recruitment. Recombinant CXCL12 (10 nM) was used as the positive control for agonism and CXCL12 (10 nM) + AMD3100 (33 nM) was the positive control for antagonism. The concentrations of CXCL12 and AMD3100 used in the agonism-antagonism screens were determined by prior titrations in the Tango CXCR4-*bla* assay. As shown in Figure 4e,f, neither agonism nor antagonism was observed for any of the hit compounds at the single concentration of 10 μM. The positive and negative controls in both assay formats were in acceptable ranges. It should be noted that while no biological activities were observed for any of the hit compounds, the Tango CXCR4-*bla* assay was used in a high-throughput screening format, and it is possible that the hit compounds might affect CXCR4 dynamics without a direct effect on β-arrestin recruitment. The findings suggest further dose–response studies of individual ligand hit compounds that might reveal biological activity.

## Conclusions

Describing the protein topology as a network of nodes is a computationally efficient approach to revealing its conformational dynamics and the residues with a high capacity to transmit a perturbation in a site to distant functional sites, indicating sites that may have allosteric roles. Our computational approach involving GNM, RIN, and SILCS was applied to the CXCR4 monomer and homodimer structures that are crucial targets in cancer and HIV infection. The combination of SILCS-Hotspots in conjunction with the network analysis identified two potential allosteric sites for the monomer and three for the homodimer on protein–phospholipid or protein–protein interfaces, where critical residues in signal propagation for G-protein activation (L244, I245, F248, and W252) and microswitch residues (S131 and Y219) also reside. Two screening campaigns were conducted, first with a larger library of FDA-approved drugs and compounds in clinical trials using Maestro-Glide, where selected high-scored ligand–protein complexes were further evaluated using MD simulations. The potential allosteric sites on the protein–lipid interface predominantly made hydrophobic interactions with the ligands, including π-cation and π–π stacking. The allosteric site in the intracellular region, MA2, involved strong H-bonds and water contacts. Hit compound interactions were consistent with those in SILCS FragMaps. A second screening campaign was conducted using SILCS-MC with a smaller library of FDA-approved compounds with compounds selected for each site based on the LGFE, LE and rSASA scores. Twenty hit compounds were selected by combining all findings from both screens while focusing on their clinical usage and possible relations with CXCR4. For instance, the antifungal drugs Itraconazole and Terconazole had high binding affinities among the hit compounds. Furthermore, Isavuconazonium interacted with a good binding affinity, as well. Triazole-containing compounds have been developed and are under investigation as cancer treatments.^133−135^ Interestingly, Itraconazole was investigated against breast cancer and found to be promising.^136,137^ HIV protease inhibitors such as Darunavir, Tipranavir, and Brecanavir (under investigation) were found among the hit compounds, an interesting finding given that CXCR4 is the second coreceptor of HIV-1 for cellular entry. *In vitro* flow-cytometry-based assays with two strategies, i.e., with mAb 12G5 and ligand CXCL12, demonstrated the inhibition or activation effect of all investigated compounds, highlighting the success of our combined computational approach to revealing putative allosteric binding sites of CXCR4. Finally, a preliminary functional assay was conducted to check for allosteric activity of the hit compounds based on CXCL12 activation of CXCR4 through β-arrestin using a single concentration of the hit compounds. The results showed a lack of an effect, which may be related to the compound concentration used, consistent with the limited extent of inhibition in the *in vitro* assay against CXCL12, as well as the nature of the functional assay itself. Significant additional experiments would be required to determine dose responses of the compounds.

## Data Availability

The crystallographic structure is available from https://www.rcsb.org. The 3D structures of the compounds are available from the ZINC15 database (https://zinc15.docking.org/). The software Glide (https://newsite.schrodinger.com/platform/products/glide/) is under license. The SILCS software suite is available at no charge to academic users from SilcsBio LLC (http://www.silcsbio.com). Molecular dynamics simulations and binding free energy calculations are realized using Desmond-Maestro under the academic license (Schrödinger Release 2020-1, D. E. Shaw Research). Molecular graphics are generated with free software PyMOL (DeLano Scientific LLC, 2002). The data underlying this study are available in the published article and its Supporting Information.

## Supporting Information Available

List of residues predicted by gaussian network model (GNM) and residue interaction network (RIN) (Table S1); summary of SILCS-Hotspots sites identified in monomer CXCR4 and adjacent critical residues (Table S2); summary of SILCS-Hotspots sites identified in homodimer CXCR4 and adjacent critical residues (Table S3); binding site residues of each pocket (Table S4); docking scores, MM/GSA energies, and medical uses of the hit compounds for monomer CXCR4 (Table S5); docking scores, MM/GSA energies, and medical uses of the hit compounds for homodimer CXCR4 (Table S6); RMSD, ligand interaction diagrams, and interaction fractions of the ligands docked in MA1 (Figures S1–S8); RMSD, ligand interaction diagrams, and interaction fractions of the ligands docked in MA2 (Figures S9–S12); RMSD, ligand interaction diagrams, and interaction fractions of the ligands docked in DA1 (Figures S11–S17); RMSD, ligand interaction diagrams, and interaction fractions of the ligands docked in DA2 (Figures S20–S26); RMSD, ligand interaction diagrams, and interaction fractions of the ligands docked in DA3 (Figures S27–S41); potential allosteric binding site MA1 of monomer CXCR4 with the hit compounds, hotspots occupied pockets of interest, FragMaps, and the selected fragments (Figure S42); SILCS docking results and medical uses of the top 20 compounds on each allosteric site for monomer CXCR4 (Table S7); SILCS docking results and medical uses of the top 20 compounds on each allosteric site for homodimer CXCR4 (Table S8); and SILCS docking results and medical uses of the top 20 compounds on orthosteric sites CXCR4 (Table S9) (PDF)

## Abbreviations

CXCR4: CXC chemokine receptor 4
CXCL12: CXC chemokine ligand 12
GNM: Gaussian network model
RIN: residue interaction network
SILCS: site identification by ligand competitive saturation
RMSD: root-mean-square deviation
MM/GBSA: molecular mechanics generalized Born surface area
MA1: allosteric site # 1 on the monomer
MA2: allosteric site # 2 on the monomer
DA1: allosteric site # 1 on the homodimer
DA2: allosteric site # 2 on the homodimer
DA3: allosteric site # 3 on the homodimer
