# Supplementary material for: Identifying and Assessing Putative Allosteric Sites and Modulators for CXCR4 Predicted through Network Modeling and Site Identification by Ligand Competitive Saturation
Source: J Phys Chem B. 2024 Apr 22;128(21):5157–74. doi: 10.1021/acs.jpcb.4c00925 (PMC11139592; doi:10.1021/acs.jpcb.4c00925)
Supplement: Supplementary file 1 — jp4c00925_si_001.pdf [file jp4c00925_si_001.pdf]

Supporting Information

Identifying and Assessing Putative Allosteric Sites and Modulators for CXCR4 Predicted Through Network Modeling and Site Identification by Ligand Competetitive Saturation

Tugce Inan[a], Robin Flinko[b], George K. Lewis[b], Alexander D. MacKerell Jr.[c] & Ozge Kurkcuoglu[a*]*

[a] Department of Chemical Engineering, Istanbul Technical University, Istanbul 34469, Turkey

[b] Institute of Human Virology, University of Maryland School of Medicine, Baltimore, MD 21201, USA

[c] University of Maryland Computer-Aided Drug Design Center, Department of Pharmaceutical Sciences, School of Pharmacy, University of Maryland, Baltimore, MD 21201, USA

Corresponding Authors: Ozge Kurkcuoglu olevitas@itu.edu.tr, and Alexander D. MacKerell Jr. alex@outerbanks.umaryland.edu

Table S1: List of residues predicted by Gaussian Network Model (GNM) and Residue Interaction Network (RIN)

	Residues at hinge regions by GNM	Residues with high distance fluctuations by GNM	Hub residues predicted by RIN
**Monomer**	Slow mode 1: G52, I53, L85, L86, T117, V118, L167, T168, I204, M205, F248-C251, C295, C296.	I48, F49, G52, I53, N56, L80, S81, A83, D84, F87, V88, V118, N119, S122-D133, A137, F248, F249, C251, F292, H294, C295, N298.	Y45, I48, N56, R77, S81, D84, F87, V88, T90, W94, H113, I115-R134, R148, V156, D171, R188, Q200, H203, L208, P211, I215, L244, A247, F248, W252, Y255-I257, I259, S260, S285-E288, A291, F292, H294-C296.
	Slow mode 2: N37, K38, R70-D74, I126, L127, V155, V156, G207-L210, C295-P299.		
	Slow mode 3: A100, N101, V114, V118, L120-S122, V124-S131, L151-V156, W161, I162, K239, T240, I259, S260, H281, K282.		
	Slow mode 4: T51, G52, F87, V88, H113, V114, D133, R134, Q145, R146, D171-A175, R188, F189, C218, Y219, F248-C251, L253-I257, C274-T279, H294, C295.		
	Slow mode 5: L50, T51, S71-T73, K75-L80, V82-L86, L166, L167, N176, V177, C186, D187, I209-P211, S229, K230, Q272, G273, E288-F292.		
**Homodimer**	Slow mode 2: I53, V54, L85, L86, T117, V118, L165, L166, M205, V206, F249, A250, C296, L297.	I48, F49, G52, G55, N56, L78, L80, S81, D84, F87, V88, I115, Y116, V118, N119, S122-L127, F129, I130, F292-C295, N298, P299	Chain A: F87, N119-I126, F129, S131, D133, H140, P147, R148, L150, L151, V156, L167, F174, A175, D186-R188, Y190-N192, W195, V198-G212, I215, W252, Y255, Y256, I259, L267, T287.<br><br>Chain B: D84, F87, N119-L132, Y135, V139, H140, F174, D186-R188, Y190-N192, W195, V198-P211, I215, C218, C220, I221, W252, Y255, Y256, I259, S260, L267.
	Slow mode 3: L58, V59, L80, S81, L120, Y121, A164, L165, Y190, P191, W195, V196, F201, Q202, I245-A247, P299-I300.		
	Slow mode 4: N37, K38, I44, R70-K75, I126, L127, L150-A152, V196-V206, H294-N298.		
	Slow mode 5: S71-D74, S123-L127, L151-K154, F189-P191, A291-C295.		

Table S2: Summary of SILCS-Hotspots sites identified in monomer CXCR4 and adjacent critical residues.

Site	Residues	Site rank	Distance (Å) btw. hot spots -closest heavy atom critical residues	Mean LGFE*	Most favorable LGFE*	Most favorable LE*	Fragment with lowest LGFE	# Fragments	The top 5 fragments
**MA1**	S131, I215	2	5.6, 3.4	-3.67	-6.9	-1.26	76	58	76>17>88>29>70
**Orthosteric site**	W94, R188	3	5.9, 6.9	-3.65	-6.63	-1.15	34	81	
**Orthosteric site**	F87, T90, W94, F292	4	5.0, 5.9, 1.7, 6.0	-3.59	-5.57	-0.93	71	13	
**MA1**	L208, F249, F248, Y256	9	3.5, 4.8, 5.2, 3.0	-3.49	-7.11	-1.05	37	86	37>50>76>50>83
**MA2**	N56, R77	19	6.9, 7.2	-3.1	-6.34	-0.84	83	65	
**MA1**	L208, I215, F249	21	6.4, 4.4, 3.7	-3.04	-4.34	-0.75	23, 28	14	23>28>14>17>17b
**MA2**	R77, D133	24	7.0, 7.4	-2.96	-5.05	-0.72	63b	59	
**MA1**	L208	35	5.0	-2.84	-5.56	-0.79	50	28	50>87>6>83>50
**MA2**	R77	74	8.0	-2.36	-2.72	-0.44	63b	5	

*in kcal/mol

Table S3: Summary of SILCS-Hotspots sites identified in homodimer CXCR4 and adjacent critical residues.

Site	Chain A	Chain B	Site rank	Distance (Å) btw. hot spots - closest heavy atom critical residues	Mean LGFE*	Most favorable LGFE*	Most favorable LE*	Fragment with lowest LGFE	# Fragments	Top 5 fragments
**DA2**	I215		2	4.9	-4.15	-7.29	-1.34	60	57	60>80>37>80>76
**DA3**		I221	4	4.1	-3.99	-6.82	-1.21	76	64	76>37>17>29>88
**DA2**	V124, L208, W252, Y256		9	5.1, 5.9, 3.9, 2.8	-3.74	-7.19	-1.438	17	59	17>26b>98>80>7
**DA1**	F199		12	6.0	-3.67	-6.52	-1.16	37	83	37>76>17>52b>80
**DA1**		F201, L267	12	4.3, 3.6	-3.67	-6.52	-1.16	37	83	37>76>17>52b>80
**DA2**	L208, I209, G212, Y256		14	3.9, 5.7, 5.3, 5.9	-3.63	-5.72	-1.01	76, 50	18	76>50>83>70>87
**DA3**		I209	23	5.2	-3.47	-5.95	-0.74	50	16	
**DA3**	L125, V206, L210, P211		30	4.0, 4.9, 2.6, 5.5	-3.3	-6.46	-1.08	37	66	37>83>76>50>83
**DA3**		L210	30	5.6	-3.3	-6.46	-1.08	37	66	37>83>76>50>83

Site	Chain A	Chain B	Site rank	Distance (Å) btw. hot spots - closest heavy atom critical residues	Mean LGFE*	Most favorable LGFE*	Most favorable LE*	Fragment with lowest LGFE	# Fragments	Top 5 fragments
**DA3**	L167, Q202, V206		34	4.6, 2.8, 4.0	-3.24	-4.55	-0.7	88	11	88>60>85>17>17b
**DA1**		F201, M205	34	4.9, 2.9	-3.24	-4.55	-0.7	88	11	88>60>85>17>17b
**DA2**	M205, I209		35	5.5, 3.3	-3.22	-6.36	-0.86	37	52	37>50>88>83>56
**DA1**		Y256, S260	49	4.8, 3	-3.07	-5.24	-0.79	83	59	83>50>76>60>74
**DA2**	L208, Y256		56	5.4	-3.02	-5.29	-0.77	50, 76	34	50>76>38>87>6
**DA1**	F174, F199		91	3.4, 4.1	-2.7	-3.43	-0.52	50	11	50>76>83>59>6
**DA3**	L125, A128, F129, L132		117	4.2, 3.5, 3.5, 4.7	-2.53	-2.94	-0.49	48	3	48>73>100
**DA3**		C220, I221	139	5.1	-2.41	-3.15	-0.35	79	5	
**DA2**		M205, V206, L210	140	3.1, 5.2, 5.0	-2.41	-2.41	-0.48	17b	1	

*in kcal/mol

Table S4. Binding site residues of each pocket.

Structure	Proposed allosteric site	Chain	Binding site residues
Monomer	MA1	A	L120, S123, V124, L127, A128, S131, G207, L208, I209, P211, G212, I213, I215, L216, S217, Y219, C220, V242, I245, L246, F248, F249, W252, Y256, H294
	MA2	A	S71, T73, D74, R77, I130, R134, A237, T240, T241, L244
Homodimer	DA1	A	W195, V198, F199
		B	V197, Q200, F201, I204, I259, S260, S263, F264, L266, L267
	DA2	A	L80, L120, S123, V124, L125, L127, A128, S131, V206, G207, L208, I209, L210, P211, G212, I213, V214, I215, L216, S217, Y219, T241, V242, I245, L246, F248, F249, W252, L253, Y256
	DA3	A	L125, V155, V158, G159, V160, W161, I162, P163, A164, L165, L166, L167, M205, L206, L210
		B	M205, L210, I213, S217, C220, I221

Underscored residues are predicted by both GNM and RIN

Table S5: Docking scores, MM/GBSA energies and medical uses of the hit compounds for monomer CXCR4.

	Proposed allosteric site	Drug name and ZINC ID	Glide Docking Score	Prime MM/GBSA *(docking pose)	MM/GBSA* (MD)	LGFE*	LE*	Status	Medical Uses
**Monomer**	*MA1*	Relugolix-ZINC43206033	-11.61	-89.458	-94.147± 6.755	-7.273	-0.165	FDA approved	GnRH receptor antagonist for androgen deprivation therapy in the treatment of advanced prostate cancer[1].
		ZINC29238439	-11.31	-102.579	-116.266± 8.742	-5.967	-0.133	Investigational only	-
		Bemcentinib-ZINC51951669	-9.03	-103.869	-102.847± 5.886	-8.059	-0.212	Investigational only	Experimental drug as AXL kinase inhibitor[2].
		Halofantrine-ZINC1542393	-8.99	-97.111	-76.590± 4.505	-6.918	-0.21	World-not-FDA	Antimalarial drug.
		Desbutyl-Lumefantrine-ZINC100054860	-8.89	-95.320	-102.524± 5.710	-6.746	-0.218	World-not-FDA	Metabolite of lumefantrine.
		Fulvestrant-ZINC3995809	-8.78	-80.330	-80.973± 6.430	-14.922	-0.364	FDA approved	To treat postmenopausal women diagnosed with metastatic hormone-receptor-positive breast cancer[3].
		Lumefantrine-ZINC100004345	-8.68	-104.432	-72.245± 6.561	-5.149	-0.147	FDA approved	Antimalarial drug.

	Sertaconazole-ZINC2016037	-8.66	-75.281	-80.174± 4.017	-7.619	-0.282	FDA approved	Antifungal drug.
	Pimavanserin-ZINC16159083	-8.64	-81.152	-58.487± 9.132	-7.534	-0.243	FDA approved	Atypical antipsychotics to treat Parkinson's disease psychosis and studied for the treatment of Alzheimer's disease, psychosis, schizophrenia , agitation, and major depressive disorder[4].
MA2	Cangrelor-ZINC85537017	-8.30	-72.965	-73.7612± 6.676	-11.538	-0.262	FDA approved	P2Y12 platelet receptor antagonist used as effective inhibitors of adenosine diphosphate-mediated platelet activation and aggregation[5,6].
	Pemetrexed-ZINC1540998	-7.58	-53.601	-49.032± 10.522	-7.589	-0.245	FDA approved	Chemotherapy drug to treat pleural mesothelio ma and non-small cell lung cancer[7].
	ZINC3830427	-6.91	-86.112	-60.002± 6.549	-8.373	-0.239	World-not-FDA	-

*in kcal/mol

Table S6: Docking scores, MM/GBSA energies and medical uses of the hit compounds for homodimer CXCR4.

	Proposed allosteric site	Drug name and ZINC ID	Glide Docking Score	Prime MM/GBSA * (docking pose)	MM/GBSA* (MD)	LGFE*	LE*	Status	Medical Uses
**Homodimer**	*DA1*	Neladenoson bialanate-ZINC68246043	-9.04	-77.040	-70.940± 5.753	-14.106	-0.288	Investigational only	Partial adenosine A1 receptor agonist, has been investigated for the treatment of heart failure[8].
		Lapatinib-ZINC1550477	-8.42	-70.694	-75.333± 4.429	-13.084	-0.327	FDA approved	Used in combination therapy for HER2-positive breast cancer[9]. Has been found that Src and CXCR4 contribute to invasiveness of breast cancer cells resistant to lapatinib[10].
		Ly377604-ZINC2005848	-8.01	-95.144	-56.027± 10.378	-11.034	-0.283	Investigational only	Has been used in trials studying the treatment of obesity[11].
		Amelubant-ZINC4392964	-7.98	-93.656	-69.966± 8.736	-11.634	-0.291	Investigational only	Long-acting oral Leukotriene B4 (LTB4) receptor antagonist[12], under investigation against asthma, chronic obstructive pulmonary disease (COPD), cystic fibrosis, and rheumatoid arthritis[13].
		Axitinib-ZINC3816287	-7.68	-70.269	-64.097± 6.914	-7.818	-0.279	FDA approved	Tyrosine kinase inhibitor of the vascular endothelial growth factor receptor[14]. It is used in

								the treatment of advanced renal cell carcinoma[15].
	Raloxifene-ZINC538275	-7.23	-73.487	-72.372± 7.513	-10.608	-0.312	FDA approved	A second-generation selective estrogen receptor modulator (SERM) is used to prevent osteoporosis in postmenopausal women. Reducing breast cancer risk in women[16].
	ZINC100015129	-7.16	-101.524	-68.565± 5.122	-10.524	-0.234	Investigational only	-
	Fulvestrant-ZINC3926298	-10.77	-92.803	-72.536± 7.274	-15.02	-0.366	FDA approved	To treat postmenopausal women diagnosed with metastatic hormone-receptor-positive breast cancer[17].
	Pipotiazine Palmitate-ZINC8214658	-10.34	-75.017	-91.008± 10.651	-14.801	-0.302	FDA approved	An antipsychotic drug is used to treat schizophrenia and other similar conditions[18].
DA2	Tipranavir-ZINC100022637	-9.63	-78.755	-65.592± 4.891	-9.72	-0.231	FDA approved	HIV protease inhibitor.
	Valrubicin-ZINC11616852	-9.51	-75.919	-90.620± 6.010	-9.044	-0.177	FDA approved	Chemotherapy for bladd er cancer treatment.
	Valategrast-ZINC72190226	-8.70	-91.704	-79.364± 5.716	-9.21	-0.23	Investigational only	Dual-acting α4/β1 - α4/β7 integrin antagonist under investigation for multiple sclerosis (MS) and asthma treatment.

	Lopinavir-ZINC3951740	-8.69	-69.538	-68.158± 4.372	-9.24	-0.201	FDA approved	HIV protease inhibitor.
	Praziquantel-ZINC403566	-8.45	-72.212	-61.595± 4.298	-7.365	-0.32	FDA approved	An anthelmintic drug used to treat a number of parasitic worm infections such as schistosomiasis.
DA3	Brecanavir-ZINC3994828	-12.21	-93.159	-95.748± 5.493	-20.251	-0.434	Investigational only	Active aspartic protease inhibitor (PI), under investigation for HIV treatment[19].
	Tariquidar-ZINC4214704	-11.51	-95.101	-76.934± 9.770	-14.661	-0.327	Investigational only	Third generation P-glycoprotein (P-gp) inhibitor, under investigation for treatment in ovarian cancer, lung cancer, and breast cancer[20].
	ZINC150339328	-11.46	-90.538	-102.450± 6.120	-19.231	-0.379	World-not-FDA	Itraconazole derivative.
	Itraconazole-ZINC4097344	-11.11	-88.317	-112.810± 8.139	-19.261	-0.382	FDA approved	An antifungal drug used in the treatment of systemic and superficial fungal infections.
	Montelukast-ZINC3831151	-10.80	-83.009	-72.912± 5.932	-12.355	-0.301	FDA approved	A leukotriene receptor antagonist used as part of an asthma therapy regimen to prevent exercise-induced bronchoconstriction and to treat seasonal allergic rhinitis[21].
	Tipranavir-ZINC100016058	-10.57	-64.126	-76.201± 10.663	-14.482	-0.332	FDA approved	HIV protease inhibitor.

Ritonavir-ZINC3944422	-10.49	-83.494	-89.916± 8.185	-12.64	-0.249	FDA approved	HIV protease inhibitor
Zafirlukast-ZINC896717	-10.23	-68.051	-89.793± 7.253	-10.52	-0.248	FDA approved	Leukotriene receptor antagonist used for prophylaxis and chronic treatment of asthma[21].
Isavuconazonium-ZINC29571072	-9.85	-79.129	-96.868± 8.841	-16.863	-0.358	FDA approved	An antifungal drug used in the treatment of invasive aspergillosis and mucormycosis[22].
Lumefantrine-ZINC100004343	-9.62	-94.317	-95.515± 7.599	-9.359	-0.274	FDA approved	Antimalarial drug.
Pimozide-ZINC4175630	-9.58	-80.844	-83.940± 5.936	-11.732	-0.331	FDA approved	An antipsychotic agent used for debilitating motor and phonic tics in Tourette's Disorder[23].
Apixaban-ZINC11677837	-9.52	-71.534	-82.588± 5.553	-10.062	-0.284	FDA approved	Factor Xa (fxa) inhibitor an anticoagulant medication used for the treatment and prevention of blood clots and stroke in people with nonvalvular atrial fibrillation[24].
Lapatinib-ZINC1550477	-9.20	-73.394	-74.188± 8.724	-12.804	-0.331	FDA approved	Used in combination therapy for HER2-positive breast cancer[9]. Has been found that Src and CXCR4 contribute to invasiveness of breast cancer cells resistant to lapatinib[10]

	Olaparib-ZINC40430143	-8.94	-79.806	-69.967± 4.278	-11.181	-0.356	FDA approved	A chemotherapeutic drug used for treatment of recurrent or advanced ovarian cancer and metastatic breast cancer in patients with specific mutations.
	Fluphenazine-ZINC19203912	-8.33	-73.621	-71.596± 5.035	-10.581	-0.351	FDA approved	An antipsychotic drug, used for treatment of chronic psychoses such as schizophrenia.

*in kcal/mol

Table S7: SILCS Docking results and medical uses of the top 20 compounds on each allosteric site for monomer CXCR4.

	Proposed allosteric site	Drug name	LGFE (kcal/mol)	LE (kcal/mol)	rSASA (%)	Medical Uses
Monomer	MA1	Landiolol hydrochloride	-15.38	-0.427	67.08	β-blocker to treat cardiac arrhythmias [25].
		Travoprost	-13.99	-0.4	78.79	Used in treating intraocular pressure related to open-angle glaucoma or ocular hypertension[26].
		Mycophenolate Mofetil	-13.7	-0.442	78.98	Inosine monophosphate dehydrogenase inhibitor to prevent organ transplantation rejection[27].
		Ruscogenin	-13.21	-0.426	66.07	Anti-inflammatory steroid.
		Delamanid	-13.06	-0.344	62.17	Used in combination with other tuberculosis drugs for active multidrug-resistant tuberculosis[28].
		Empagliflozin BI10773	-13.04	-0.421	87.18	Selective inhibitor of sodium-glucose cotransporter 2[29].
		Tribenzagan Hydrochloride	-12.88	-0.46	91.71	Antiemetic agent for the treatment of nausea and vomiting[30].
		Topiramate	-12.84	-0.584	85.89	For the control of epilepsy attacks and in the prophylaxis and treatment of migraines[31]
		Terconazole	-12.75	-0.354	90.88	Antifungal.
		Cilostazol	-12.68	-0.47	88.89	Vasodilator.
		Retapamulin	-12.47	-0.346	70.09	Antibacterial agent against superficial skin infections caused by staphylococcus aureus[32].
		Darunavir	-12.37	-0.326	80.49	HIV protease inhibitor.
		Calcitriol	-12.24	-0.408	58.41	The active form of vitamin D.
		BAF312 Siponimod	-12.08	-0.326	70.17	For the treatment of multiple sclerosis[33].
		Fumagillin	-12.07	-0.366	65.27	An antimicrobial agent.
		Loganin	-12.05	-0.446	73.72	Iridoid glycoside, used as a neuroprotective and anti-inflammatory agent [34].
		Maraviroc	-11.89	-0.321	79.08	CCR5 antagonist to treat HIV infection[35].
		Pravastatin	-11.8	-0.393	76.85	Statin drug to reduce the level of blood lipids.
		Rosuvastatin	-11.77	-0.357	75.42	Statin drug to reduce the level of blood lipids.
		Dehydrocholic acid	-11.35	-0.391	79.94	
	MA2	Citicoline sodium	-9.52	-0.307	76.31	Under investigations against mania, stroke, hypomania, cocaine abuse, and bipolar disorder[13].
		Delamanid	-9.33	-0.245	82.28	Used in combination with other tuberculosis drugs for active multidrug-resistant tuberculosis[28].
		Sofalcone	-9.32	-0.282	73.48	Gastric mucosa protective agent[36].

	Raltitrexed	-9.3	-0.291	71.71	An antimetabolite for cancer treatment[37].
	Dibutyryl-cAMP Bucladesine	-8.8	-0.275	73.92	Cyclic nucleotide derivative used as phosphodiesterase inhibitor[38] .
	Cefonicid sodium	-8.76	-0.25	80.86	Second-generation cephalosporin against bacteria.
	Alprostadil	-8.27	-0.331	84.52	Used for erectile dysfunction treatment[39].
	Pranlukast	-8.19	-0.227	79.37	Leukotriene receptor antagonist against allergic rhinitis[40] and asthma[41].
	Travoprost	-8.19	-0.234	75.6	Used in treating intraocular pressure related to open-angle glaucoma or ocular hypertension[42].
	Valganciclovir HCl	-7.9	-0.316	68.14	Antiviral.
	VX-809 Lumacaftor	-7.84	-0.238	70.84	An experimental drug for the treatment of cystic fibrosis[43].
	Cidofovir	-7.75	-0.431	83.21	Antiviral.
	Aspartame	-7.69	-0.366	85.75	Artificial sweetener.
	Glutathione	-7.65	-0.383	70.4	Antioxidant.
	Calcitriol	-7.49	-0.25	86.99	The active form of vitamin D.
	Furaltadone HCl	-7.49	-0.326	67.69	
	Furosemide	-7.46	-0.355	65.45	Diuretic drug.
	Tivozanib AV-951	-7.37	-0.23	66.71	VEGFR inhibitor.
	Tedizolid Phosphate	-7.34	-0.237	61.57	Antibiotic against gram-positive bacterial infections.
	Famciclovir	-7.33	-0.319	71.00	Antiviral for the treatment of herpes.

Table S8: SILCS Docking results and medical uses of the top 20 compounds for homodimer CXCR4.

	Proposed allosteric pocket	Drug name	LGFE (kcal/mol)	LE (kcal/mol)	rSASA (%)	Medical Uses
		Landiolol hydrochloride	-16.67	77.66	-0.463	β-blocker to treat cardiac arrhythmias[25].
		Epirubicin HCl	-16.24	77.99	-0.417	Anthracycline topoisomerase inhibitor for cancer treatment[44].
		Darunavir	-16.21	80.35	-0.427	HIV protease inhibitor.
		Mycophenolate Mofetil	-15.76	79.83	-0.508	Inosine monophosphate dehydrogenase inhibitor to prevent organ transplantation rejection[27].
		Loganin	-15.5	82.86	-0.574	Iridoid glycoside used as a neuroprotective and anti-inflammatory agent[34].
		Empagliflozin BI10773	-15.08	80.61	-0.487	Selective inhibitor of sodium-glucose cotransporter 2[29].
		Maraviroc	-15.07	79.53	-0.407	CCR5 antagonist to treat HIV infection[35].
		Vilanterol Trifenate	-14.94	82.77	-0.467	Long-acting β-2 adrenoceptor agonist against chronic obstructive pulmonary disease[45].
		Ticagrelor	-14.82	66.4	-0.412	P2Y12 platelet inhibitor against myocardial infarction and stroke[46].
**Homodimer**	*DA1*	Dipyridamole	-14.7	79.1	-0.408	To inhibit blood clotting[47].
		Avanafil	-14.5	83.04	-0.427	PDE5 inhibitor against erectile dysfunction[48].
		Epicatechin gallate	-14.43	82.93	-0.451	Flavonoid derivative.
		Homoharringtonine	-14.42	78.62	-0.37	An alkaloid with antitumor activity[49].
		Boceprevir	-14.4	79.95	-0.389	Anti-HCV agent.
		Dehydroandrographolide Succinate	-14.37	73.73	-0.378	
		Travoprost	-14.34	84.42	-0.41	Used in treating intraocular pressure related to open-angle glaucoma or ocular hypertension[42].
		Capecitabine	-14.2	74.79	-0.568	Chemotherapy agent for breast cancer[50], gastric cancer[51] and colorectal cancer[52].
		Vorapaxar	-14.11	78.33	-0.392	Antiplatelet agent.
		GinkgolideC	-14.06	79.68	-0.453	Diterpenoid extracted from Ginkgo biloba.
		Rosuvastatin	-14.02	83.92	-0.425	Statin drug to reduce the level of blood lipids.
		Landiolol hydrochloride	-16.34	69.25	-0.454	β-blocker to treat cardiac arrhythmias[25].
		Mycophenolate Mofetil	-13.28	68.16	-0.428	Inosine monophosphate dehydrogenase inhibitor to prevent organ transplantation rejection[27].
	DA2	Delamanid	-13.08	71.57	-0.344	Used in combination with other tuberculosis drugs for active multidrug-resistant tuberculosis[28].
		Empagliflozin.BI10773	-12.15	73.4	-0.392	Selective inhibitor of sodium-glucose cotransporter 2[29].
		Calcitriol	-12.06	67.75	-0.402	The active form of vitamin D.

	Testosterone Enanthate	-11.91	65.82	-0.411	Androgen and anabolic steroids.
	Vilanterol Trifenate	-11.81	72.92	-0.369	Long-acting β-2 adrenoceptor agonist against chronic obstructive pulmonary disease[45].
	BAF312.Siponimod	-11.8	63.56	-0.319	Used for the treatment of multiple sclerosis.
	Fumagillin	-11.62	57.67	-0.352	An antimicrobial agent.
	Darunavir	-11.31	67.21	-0.298	HIV protease inhibitor
	Ulipristal	-11.24	60.48	-0.321	Selective progesterone receptor modulator
	Sofalcone	-11.22	57.47	-0.34	Gastric mucosa protective agent[36]
	Pravastatin	-11.18	61.19	-0.373	Statin drug to reduce the level of blood lipids.
	Travoprost	-11.17	68.48	-0.319	Used in the treatment of intraocular pressure related to open-angle glaucoma or ocular hypertension[42].
	Dehydroandrographolide Succinate	-11.04	52.5	-0.291	
	Cyclofenil	-10.92	71.82	-0.404	Selective estrogen receptor modulator.
	Dehydrocholic acid	-10.76	62.27	-0.371	
	Ruscogenin	-10.7	61.2	-0.345	Anti-inflammatory steroid.
	Pranlukast	-10.67	60.05	-0.296	Leukotriene receptor antagonist against allergic rhinitis and asthma[41].
	Cinepazide maleate	-10.39	62.22	-0.346	Vasodilator agent.
DA3	Landiolol hydrochloride	-18.88	74.69	-0.525	β-blocker to treat cardiac arrhythmias[25]
	Retapamulin	-16.65	76.18	-0.462	Antibacterial agent against superficial skin infections caused by Staphylococcus aureus[32].
	Darunavir	-16.09	79.86	-0.424	HIV protease inhibitor.
	Vilanterol Trifenate	-16	74.44	-0.5	Long-acting β-2 adrenoceptor agonist against chronic obstructive pulmonary disease[45].
	Delamanid	-15.58	71.83	-0.41	Used in combination with other tuberculosis drugs for active multidrug-resistant tuberculosis[28].
	Maraviroc	-15.54	79.63	-0.42	CCR5 antagonist to treat HIV infection[35].
	Pranlukast	-15.29	70.99	-0.425	Leukotriene receptor antagonist against allergic rhinitis and asthma[41].
	Dehydroandrographolide Succinate	-15.26	75.9	-0.402	
	Travoprost	-15	79.73	-0.429	Used in the treatment of intraocular pressure related to open-angle glaucoma or ocular hypertension[42].
	Terconazole	-14.75	75.73	-0.41	Antifungal.
	Fumagillin	-14.64	71.58	-0.444	An antimicrobial agent.
	Testosterone Enanthate	-14.62	78.14	-0.504	Androgen and anabolic steroids.
	Mycophenolate Mofetil	-14.55	73.86	-0.469	Inosine monophosphate dehydrogenase inhibitor to prevent organ transplantation rejection[27].
	Empagliflozin.BI10773	-14.4	77.35	-0.465	Selective inhibitor of sodium glucose cotransporter 2[29].
	Sofalcone	-14.28	78.09	-0.433	Gastric mucosa protective agent[36].

	Vorapaxar	-14.27	80.30	-0.396	Antiplatelet agent.
	Ruscogenin	-14.12	84.88	-0.456	Anti-inflammatory steroid.
	Epirubicin HCl	-13.92	81.73	-0.357	Anthracycline topoisomerase inhibitor for cancer treatment[44].
	Rocuronium Bromide	-13.9	77.71	-0.366	Neuromuscular blocking agent.
	Gallamine Triethiodide	-13.88	74.27	-0.386	Neuromuscular blocking agent.

Table S9: SILCS Docking results and medical uses of the top 20 compounds on orthosteric site of CXCR4.

		Drug name	LGFE (kcal/mol)	LE (kcal/mol)	rSASA (%)	Medical Uses
**Monomer**		Succinylcholine Chloride Dihydrate	-11	84.61	-0.577	Neuromuscular blocking agent.
		Entecavir	-10.94	84.87	-0.547	Antiviral against hepatitis B
		Emedastine	-10.85	82.3	-0.493	Antihistamine for allergic conjunctivitis treatment.
		Tebipenem Pivoxil	-10.75	81	-0.326	Broad spectrum antibiotic[53] .
		Oseltamivir Phosphate	-10.52	85.59	-0.478	Antiviral.
		Spectinomycin 2HCl	-10.43	80.86	-0.454	An antibiotic against gram-negative bacteria.
		Valganciclovir HCl	-10.17	86.31	-0.407	Antiviral.
		Topiramate	-10.05	86.99	-0.457	To control epilepsy attacks and in the prophylaxis and treatment of migraines[31].
		Vardenafil HCl Trihydrate	-9.98	78.46	-0.294	Phosphodiesterase type 5 (PDE5) inhibitors act as a vasodilator.
		Capecitabine	-9.83	85.93	-0.393	Chemotherapy agent for breast cancer[50], gastric cancer[51] and colorectal cancer[52].
		Vilanterol Trifenate	-9.8	80.76	-0.306	Long-acting β-2 adrenoceptor agonist against chronic obstructive pulmonary disease[45].
		Furaltadone HCl	-9.73	86.26	-0.423	Antibiotic.
		Sitagliptin	-9.61	87.17	-0.343	Antidiabetic drug.
		Fluvoxamine maleate	-9.45	83.09	-0.43	Antidepressant of the selective serotonin reuptake inhibitor (SSRI).
		Landiolol hydrochloride	-9.45	84.36	-0.263	β-blocker to treat cardiac arrhythmias[25].
		Silodosin	-9.45	90.18	-0.270	α1A-adrenoceptor antagonist, been marketed for benign prostatic hyperplasia[54].
		Xanthinol Nicotinate	-9.44	84.42	-0.429	Vasodilator drug.
		Acebutolol HCl	-9.37	85.27	-0.391	β-blocker against hypertension and arrhythmias.
		Arteether	-9.37	90.16	-0.426	Antimalarial.
		Almotriptan Malate	-9.29	83.15	-0.404	For the treatment of migraine symptoms[13].
**Homodimer**	*Chain A*	Citicoline sodium	-13.17	81.18	-0.425	Under investigations against mania, stroke, hypomania, cocaine abuse, and bipolar disorder[13].
		Entecavir	-12.68	81.26	-0.634	Antiviral against hepatitis B.
		Landiolol hydrochloride	-11.27	79.94	-0.313	β-blocker to treat cardiac arrhythmias[25].
		Vardenafil HCl Trihydrate	-11.21	81.1	-0.330	Phosphodiesterase type 5 (PDE5) inhibitors act as a vasodilator.
		Xanthinol Nicotinate	-10.81	77.44	-0.491	Vasodilator drug.
		Capecitabine	-10.79	85.8	-0.432	Chemotherapy agent for breast cancer[50], gastric cancer[51], and colorectal cancer[52]

	Raltegravir MK-0518	-10.73	78.42	-0.335	Antiviral for HIV.
	Nizatidine	-10.59	87.24	-0.504	Histamine H2 receptor antagonist for peptic ulcer treatment[55].
	Azathioprine	-10.46	79.14	-0.550	Immunosuppressive drug.
	Vilazodone	-10.42	82.26	-0.316	Drug for the treatment of major depression[56].
	Succinylcholine Chloride Dihydrate	-10	84.3	-0.511	Neuromuscular blocking agent.
	Topiramate	-9.81	84.06	-0.446	For the control of epilepsy attack and in the prophylaxis and treatment of migraines.
	Ganciclovir sodium	-9.68	82.4	-0.538	Antiviral
	Tivozanib AV-951	-9.68	81.45	-0.303	VEGFR inhibitor
	Istradefylline	-9.63	79.23	-0.344	Drug for Parkinson disease.
	Diazolidinyl urea	-9.61	85.76	-0.506	Antimicrobial preservative
	Paliperidone	-9.59	81.38	-0.309	Antipsychotic drug.
	Ruxolitinib Phosphate	-9.56	86.59	-0.416	JAK inhibitor, used for the treatment of myelofibrosis[57].
	Fludarabine	-9.5	84.99	-0.475	Chemotherapy drug for chronic lymphocytic leukemia treatment[58].
	Riociguat BAY63-2521	-9.5	87.39	-0.306	Used for pulmonary hypertension treatment[59].
Chain B	Entecavir	-11.18	77.46	-0.559	Antiviral drug against hepatitis B.
	Emedastine	-9.6	86.67	-0.436	Antihistamine for allergic conjunctivitis treatment.
	Voglibose	-9.44	83.93	-0.524	Alpha-glucosidase inhibitor used for the diabetes mellitus treatment[60].
	Succinylcholine Chloride Dihydrate	-9	84.29	-0.471	Neuromuscular blocking agent.
	Xanthinol Nicotinate	-8.89	86.93	-0.404	Vasodilator drug.
	Capecitabine	-8.85	83.83	-0.354	Chemotherapy agent for breast cancer[50], gastric cancer[51] and colorectal cancer[52].
	Saxagliptin hydrate	-8.77	88.77	-0.381	Selective and reversible DPP4 inhibitor.
	Pyrithioxin	-8.66	81.67	-0.361	Neurotropic agent.
	Dicyclomine HCl	-8.52	86.77	-0.387	Used to treat irritable bowel syndrome.
	Pravastatin	-8.46	85.37	-0.282	Statin drug to reduce the level of blood lipids.
	Mirabegron	-8.45	81.27	-0.302	Drug to suppress symptoms of overactive bladder[61].
	Alprostadil	-8.42	87.03	-0.337	Vasodilator agent.
	Nizatidine	-8.41	77.35	-0.400	Histamine H2 receptor antagonist for peptic ulcer treatment[55].
	Marimastat BB-2516	-8.31	85.28	-0.361	Anticancer drug.
	Landiolol hydrochloride	-8.18	83.6	-0.227	β-blocker to treat cardiac arrhythmias[25].
	Penbutolol Sulfate	-8.08	87.16	-0.385	β-blocker used to treat hypertension.
	Glycopyrrolate	-7.92	88.61	-0.345	Drug for peptic ulcer treatment.
	Vilanterol Trifenate	-7.89	82.76	-0.247	Long-acting β-2 adrenoceptor agonist against chronic obstructive pulmonary disease[45].
	Loganin	-7.88	87.33	-0.292	Iridoid glycoside used as neuroprotective and anti-inflammatory agent[34].
	Spectinomycin 2HCl	-7.85	85.7	-0.341	An antibiotic against gram-negative bacteria.

Table S10: ANOVA and Multiple Comparisons of 12G5 and CXCL12 Binding Data

ANOVA summary CXCL12		Dunnett's Multiple Comparison test	Mean Diff.	95.00% CI of diff.	Below threshold?	Summary	Adjusted P Value
F	100.3	pos vs. neg	36344	32947 to 39741	Yes	****	<0.0001
P value	<0.0001	pos vs. 1	2841	-556.0 to 6239	No	ns	0.1541
P value summary	****	pos vs. 2	2807	-590.6 to 6204	No	ns	0.1643
Significant diff. among means (P < 0.05)?	Yes	pos vs. 3	2854	-543.0 to 6252	No	ns	0.1504
R squared	0.9795	pos vs. 4	3084	-313.0 to 6482	No	ns	0.0964
		pos vs. 5	3510	112.7 to 6907	Yes	*	0.039
Brown-Forsythe test		pos vs. 6	2504	-893.0 to 5902	No	ns	0.2768
F (DFn, DFd)	1.408 (21, 44)	pos vs. 7	4174	776.7 to 7571	Yes	**	0.0079
P value	0.1669	pos vs. 8	4531	1133 to 7928	Yes	**	0.0031
P value summary	ns	pos vs. 9	221.3	-3176 to 3619	No	ns	>0.9999
Are SDs significantly different (P < 0.05)?	No	pos vs. 10	2649	-748.3 to 6046	No	ns	0.2174
		pos vs. 11	4615	1217 to 8012	Yes	**	0.0025
		pos vs. 12	2282	-1115 to 5680	No	ns	0.3889
		pos vs. 13	4950	1553 to 8347	Yes	**	0.001
		pos vs. 14	3206	-191.0 to 6604	No	ns	0.0751
		pos vs. 15	3056	-341.0 to 6454	No	ns	0.1019
		pos vs. 16	5421	2024 to 8819	Yes	***	0.0003
		pos vs. 17	7742	4344 to 11139	Yes	****	<0.0001
		pos vs. 18	7469	4072 to 10867	Yes	****	<0.0001
		pos vs. 19	1517	-1880 to 4915	No	ns	0.8683
		pos vs. 20	-9386	-12784 to -5989	Yes	****	<0.0001

ANOVA summary for 12G5 Binding		Dunnett's Multiple Comparison test	Mean Diff.	95.00% CI of diff.	Below threshold?	Summary	Adjusted P Value
F	29.36	pos vs. 1	2915	658.9 to 5170	Yes	**	0.0047
P value	<0.0001	pos vs. 2	2179	-76.79 to 4435	No	ns	0.064
P value summary	****	pos vs. 3	2131	-125.1 to 4386	No	ns	0.0744
Significant diff. among means (P < 0.05)?	Yes	pos vs. 4	3233	977.2 to 5489	Yes	**	0.0013
R squared	0.9333	pos vs. 5	2412	155.9 to 4667	Yes	*	0.0297
		pos vs. 6	3644	1389 to 5900	Yes	***	0.0002
Brown-Forsythe test		pos vs. 7	4860	2604 to 7115	Yes	****	<0.0001
F (DFn, DFd)	0.5835 (20, 42)	pos vs. 8	5555	3299 to 7810	Yes	****	<0.0001
P value	0.9024	pos vs. 9	5519	3263 to 7774	Yes	****	<0.0001
P value summary	ns	pos vs. 10	7229	4973 to 9485	Yes	****	<0.0001
Are SDs significantly different (P < 0.05)?	No	pos vs. 11	7505	5249 to 9760	Yes	****	<0.0001
		pos vs. 12	6428	4172 to 8684	Yes	****	<0.0001
		pos vs. 13	5325	3070 to 7581	Yes	****	<0.0001
		pos vs. 14	5178	2922 to 7433	Yes	****	<0.0001
		pos vs. 15	6535	4280 to 8791	Yes	****	<0.0001
		pos vs. 16	6002	3747 to 8258	Yes	****	<0.0001
		pos vs. 17	10032	7776 to 12288	Yes	****	<0.0001
		pos vs. 18	8639	6383 to 10895	Yes	****	<0.0001
		pos vs. 19	9988	7732 to 12244	Yes	****	<0.0001
		pos vs. 20	10312	8057 to 12568	Yes	****	<0.0001

Table S11: SILCS Docking LGFE scores (kcal/mol) of hit compounds to 12G5 and CXCL12 binding sites

	**Monomer**		**Dimer**			
**Drug name**	12G5	CXCL12	12G5 (chain A)	12G5 (chain B)	CXCL12 (chain A)	CXCL12 (chain B)
**Darunavir**	-4.53	-11.02	-8.61	-3.64	-16.11	-13.77
Sofalcone	-4.36	-10.94	-10.57	-8.43	-11.87	-10.06
Delamanid	-6.92	-9.79	-11.85	-9.06	-12.67	-9.89
Terconazole	-7.23	-9.94	-10.70	-8.20	-12.02	-9.68
Loganin	-5.42	-11.69	-7.74	-6.52	-15.32	-14.05
Retapamulin	-3.64	-9.09	-8.74	-6.19	-11.88	-9.42
Testosterone Enanthate	-3.75	-10.15	-6.03	-3.72	-10.51	-11.81
Travoprost	-4.54	-12.83	-9.82	-8.92	-13.25	-13.51
Empagliflozin	-5.41	-10.83	-10.63	-5.52	-14.25	-12.10
Dehydroandrographolide Succinate	-4.05	-10.77	-6.81	-3.27	-12.46	-13.96
TribenzaganHydrochloride	-7.00	-10.66	-10.80	-7.85	-10.74	-8.49
Landiololhydrochloride	-10.19	-17.07	-16.29	-12.98	-15.77	-13.49
Maraviroc	-5.42	-9.93	-11.35	-4.50	-12.72	-13.78
Rosuvastatin	-4.13	-7.76	-8.51	-5.12	-12.75	-14.05
Pranlukast	-6.77	-8.70	-11.82	-7.85	-13.75	-9.06
Dipyridamole	-3.46	-7.24	-5.82	-3.45	-13.31	-10.86
Topiramate	-6.18	-12.14	-9.81	-5.56	-11.99	-11.73
Mycophenolate Mofetil	-8.30	-9.76	-13.10	-7.38	-13.96	-12.24
Capecitabine	-7.61	-10.64	-10.78	-9.05	-14.35	-13.99
Cilostazol	-7.29	-10.61	-9.76	-8.75	-13.60	-11.29

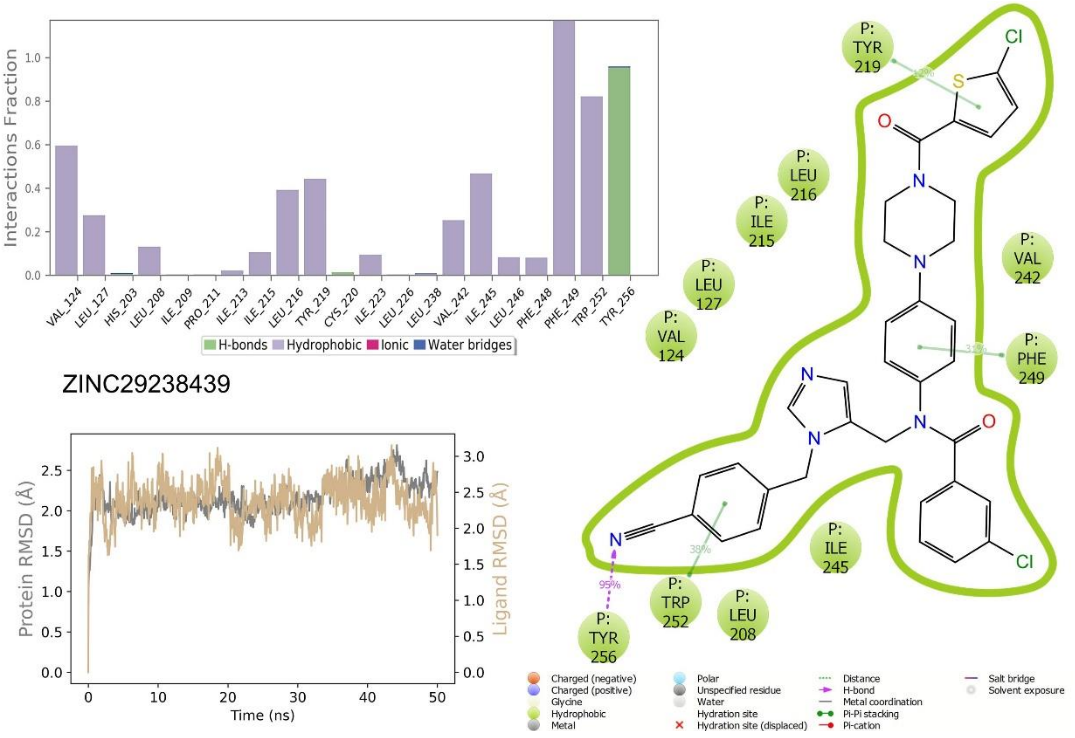

Fig. S1. RMSD, ligand interaction diagrams and interaction fractions of ZINC29238439 docked in MA1.

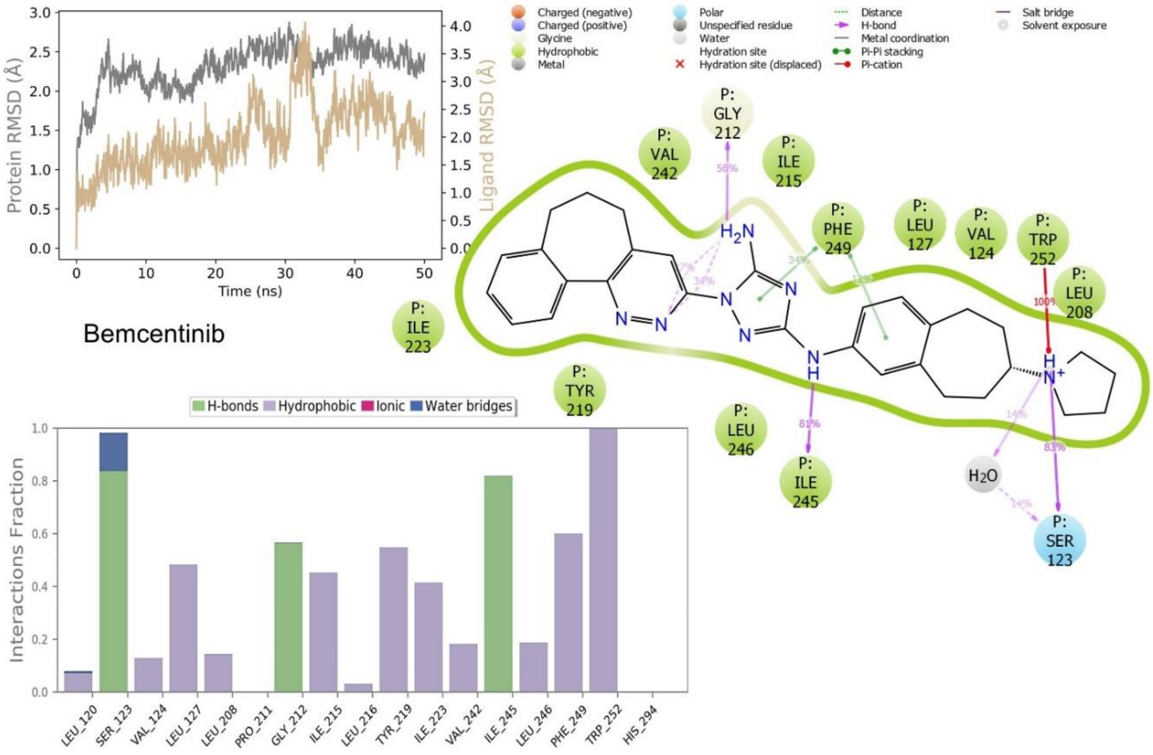

Figure S2. RMSD, ligand interaction diagrams and interaction fractions of Bemcentinib docked in MA1.

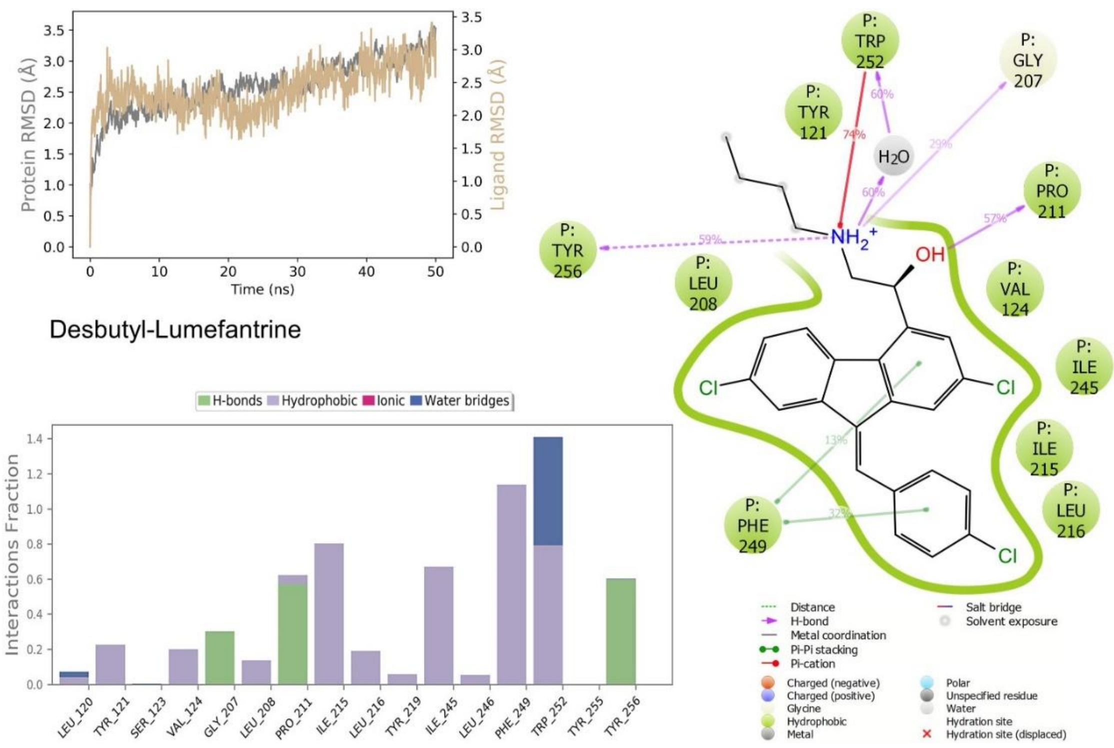

Figure S3. RMSD, ligand interaction diagrams and interaction fractions of Desbutyl-Lumefantrine docked in MA1.

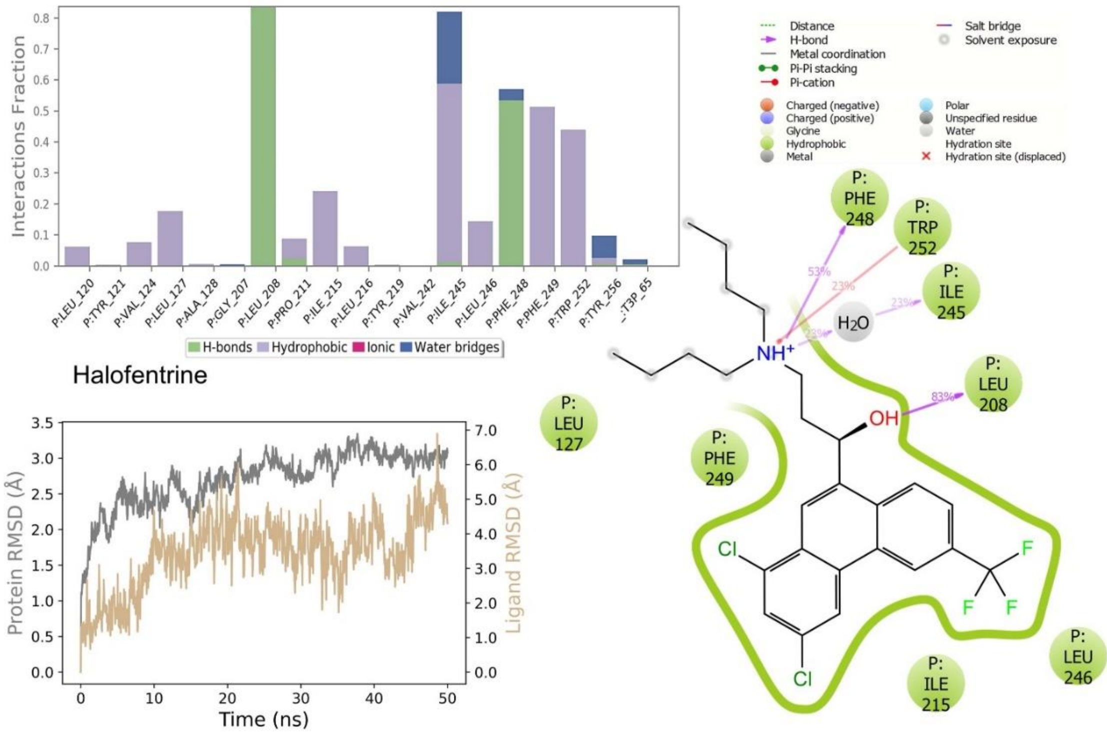

Figure S4. RMSD, ligand interaction diagrams and interaction fractions of Halofentrine docked in MA1.

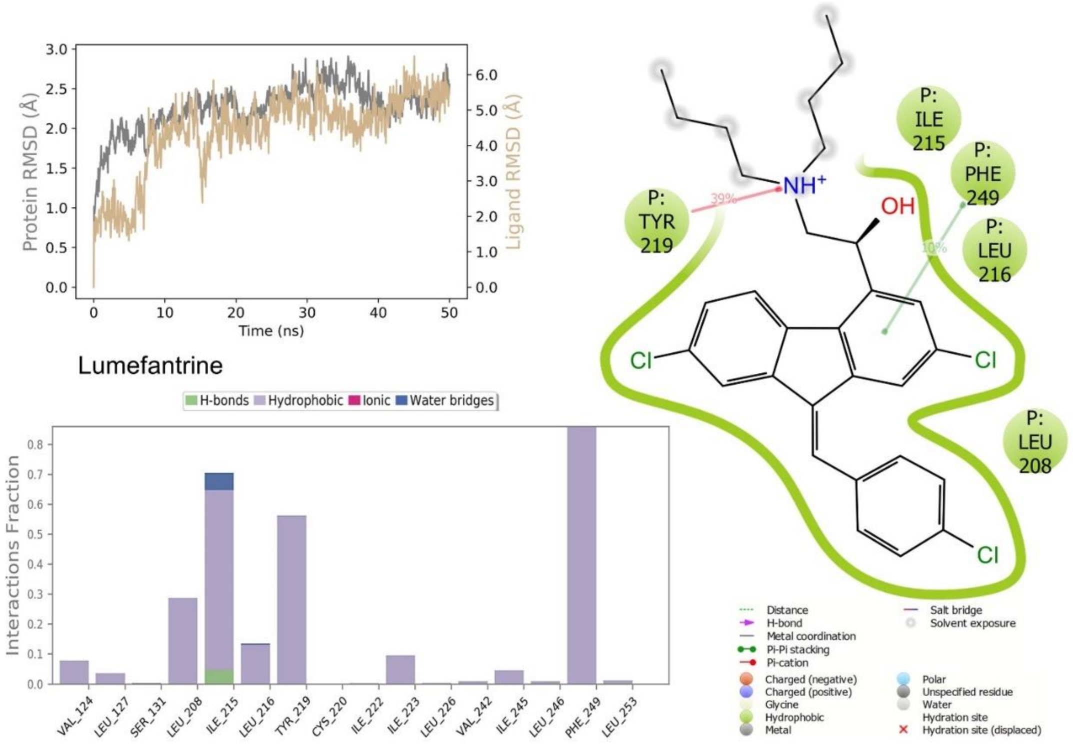

Figure S5. RMSD, ligand interaction diagrams and interaction fractions of Lumefantrine docked in MA1.

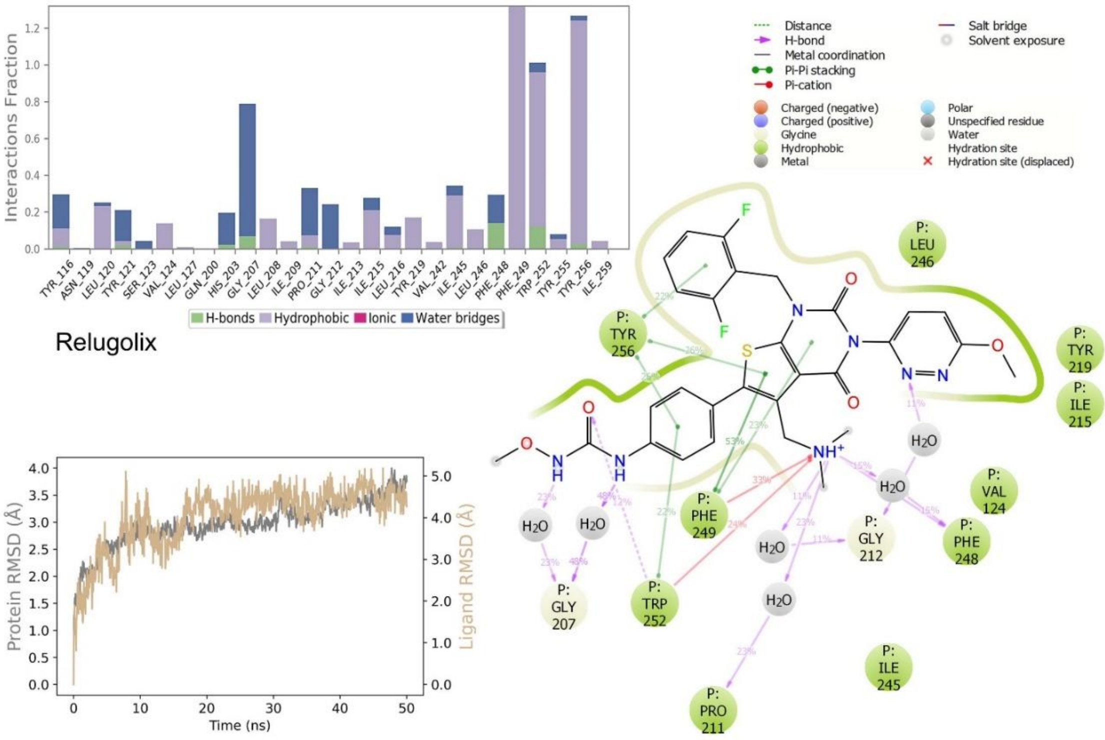

Figure S6. RMSD, ligand interaction diagrams and interaction fractions of Relugolix docked in MA1.

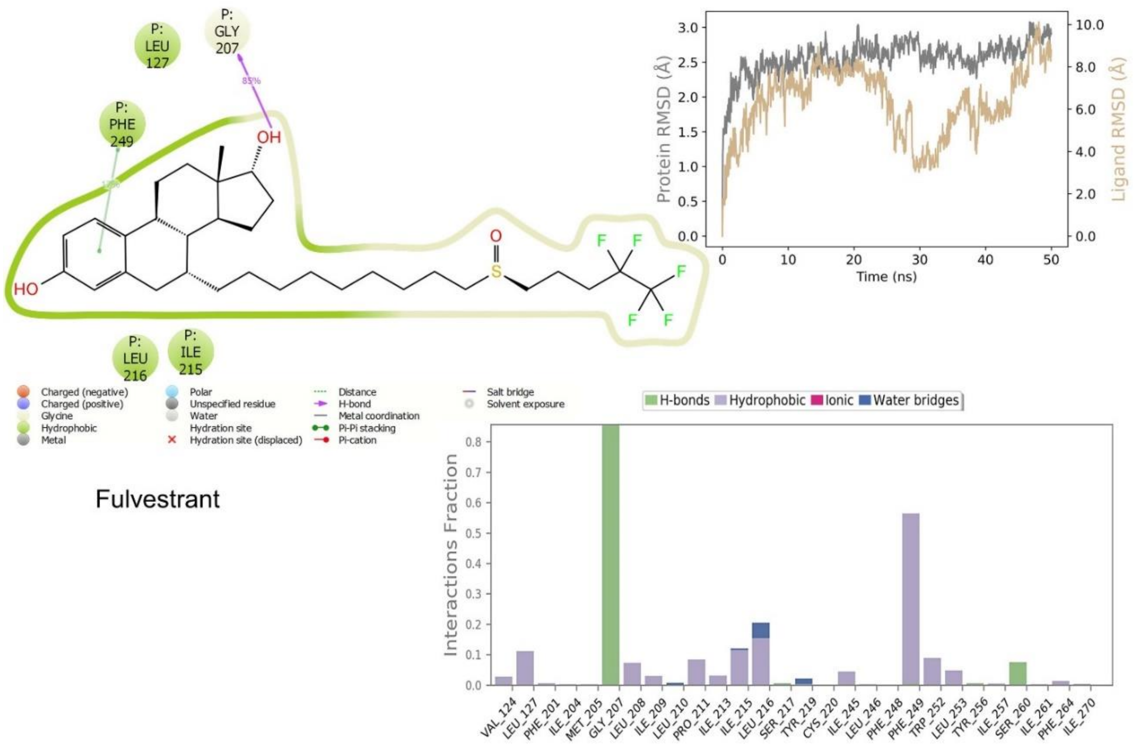

Figure S7. RMSD, ligand interaction diagrams and interaction fractions of Fulvestrant docked in MA1.

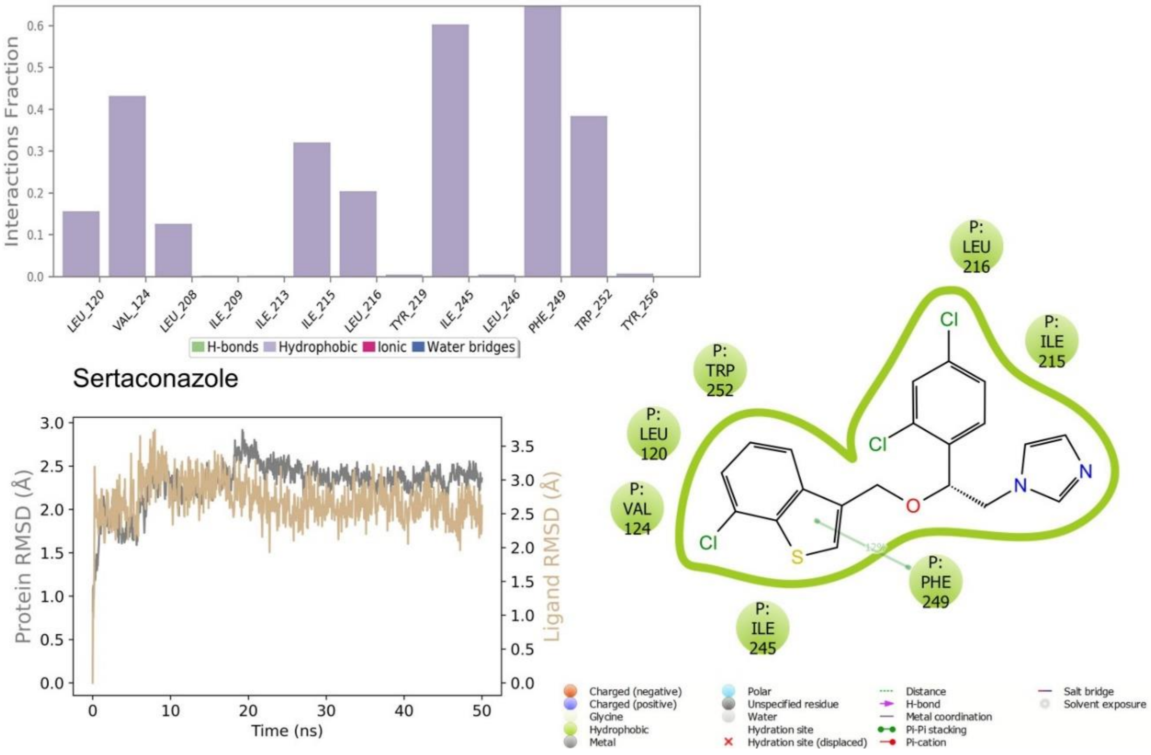

Figure S8. RMSD, ligand interaction diagrams and interaction fractions of Sertaconazole docked in MA1.

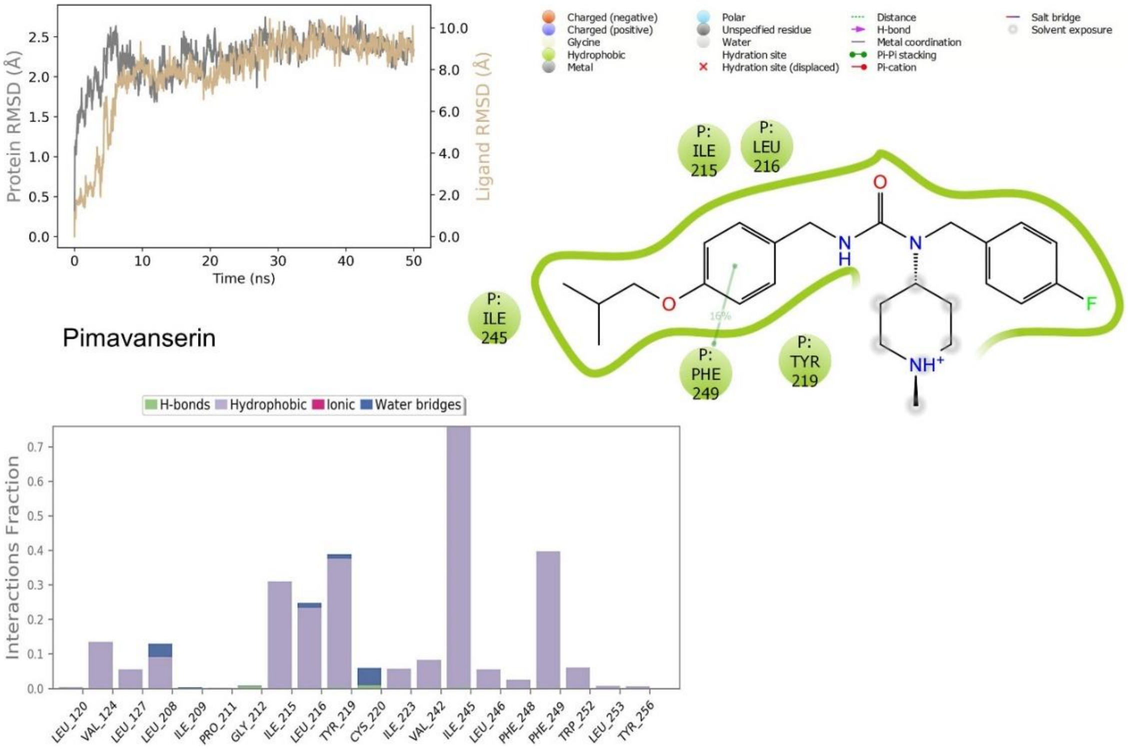

Figure S9. RMSD, ligand interaction diagrams and interaction fractions of Pimavanserin docked in MA1.

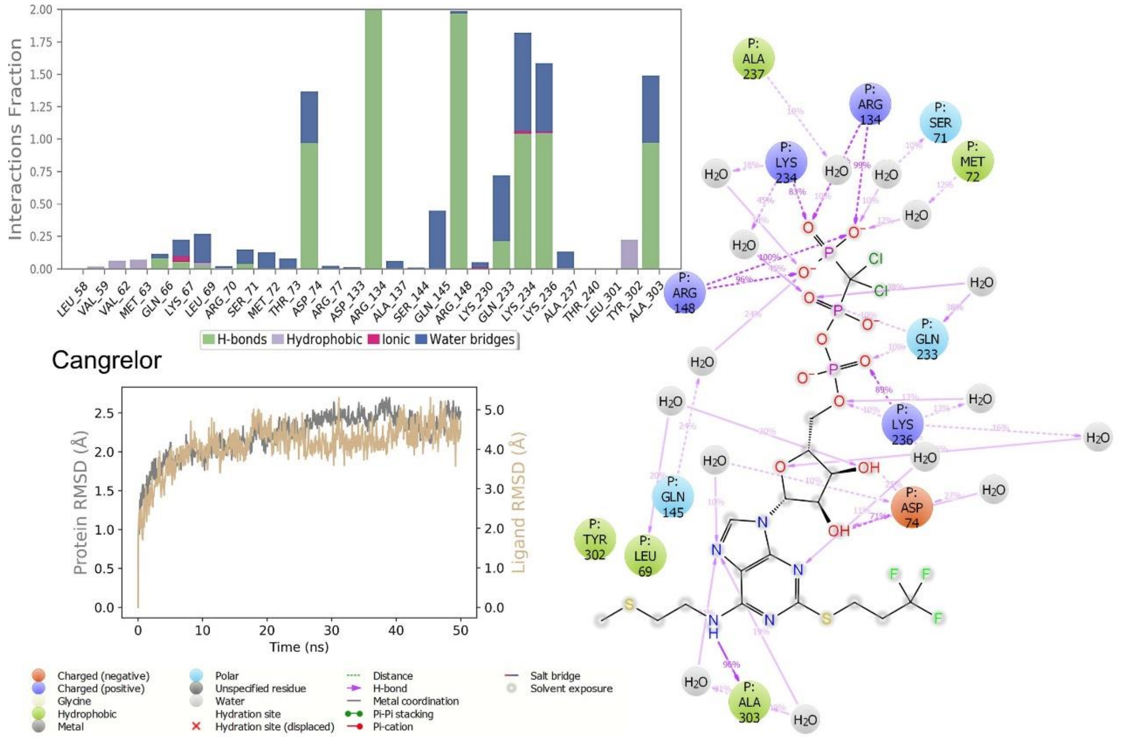

Figure S10. RMSD, ligand interaction diagrams and interaction fractions of Cangrelor docked in MA2.

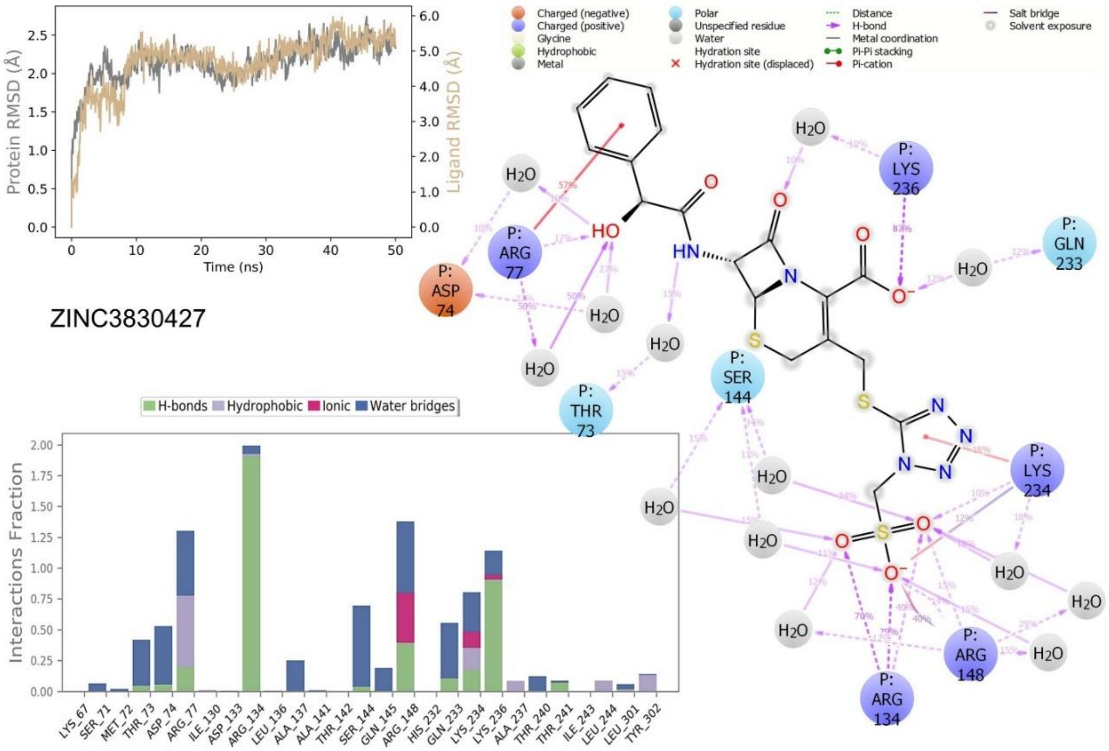

Figure S11. RMSD, ligand interaction diagrams and interaction fractions of ZINC3830427 docked in MA2.

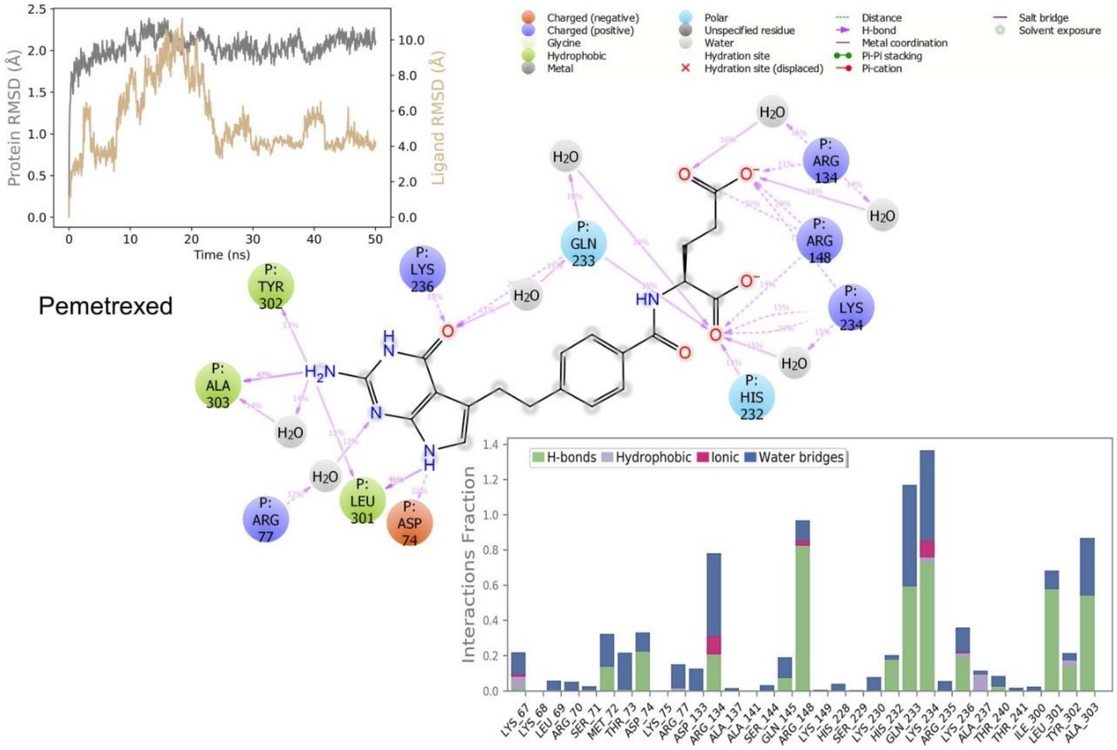

Figure S12. RMSD, ligand interaction diagrams and interaction fractions of Pemetrexed docked in MA2.

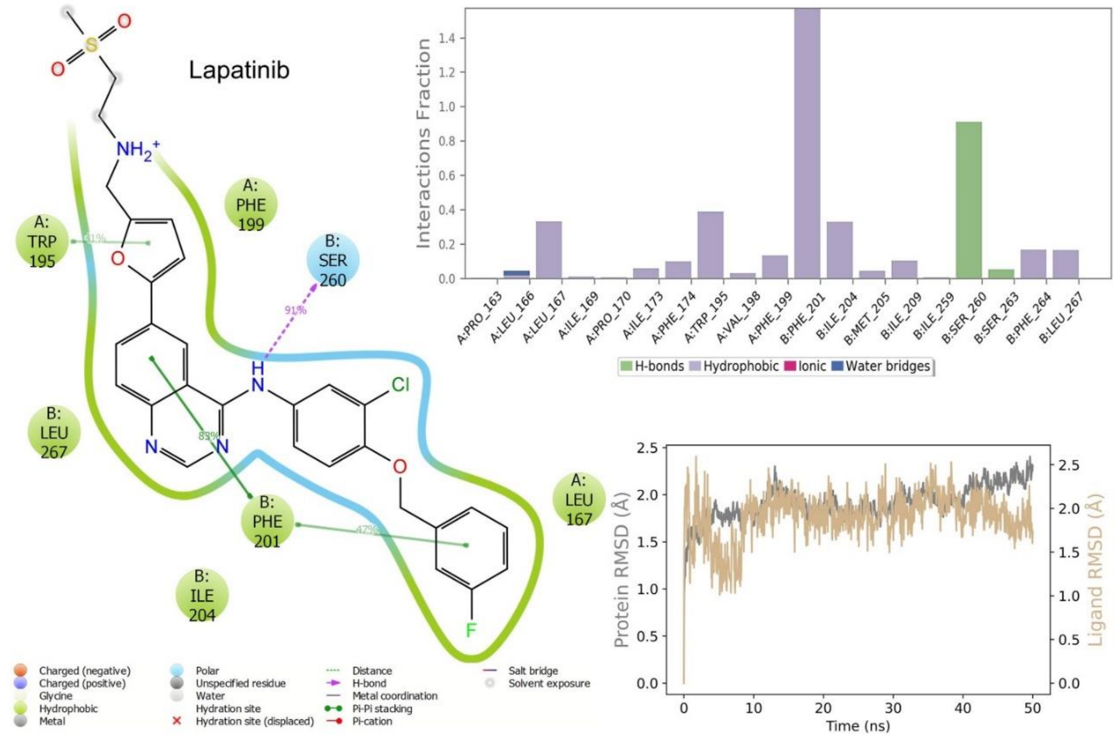

Figure S13. RMSD, ligand interaction diagrams and interaction fractions of Lapatinib docked in DA1.

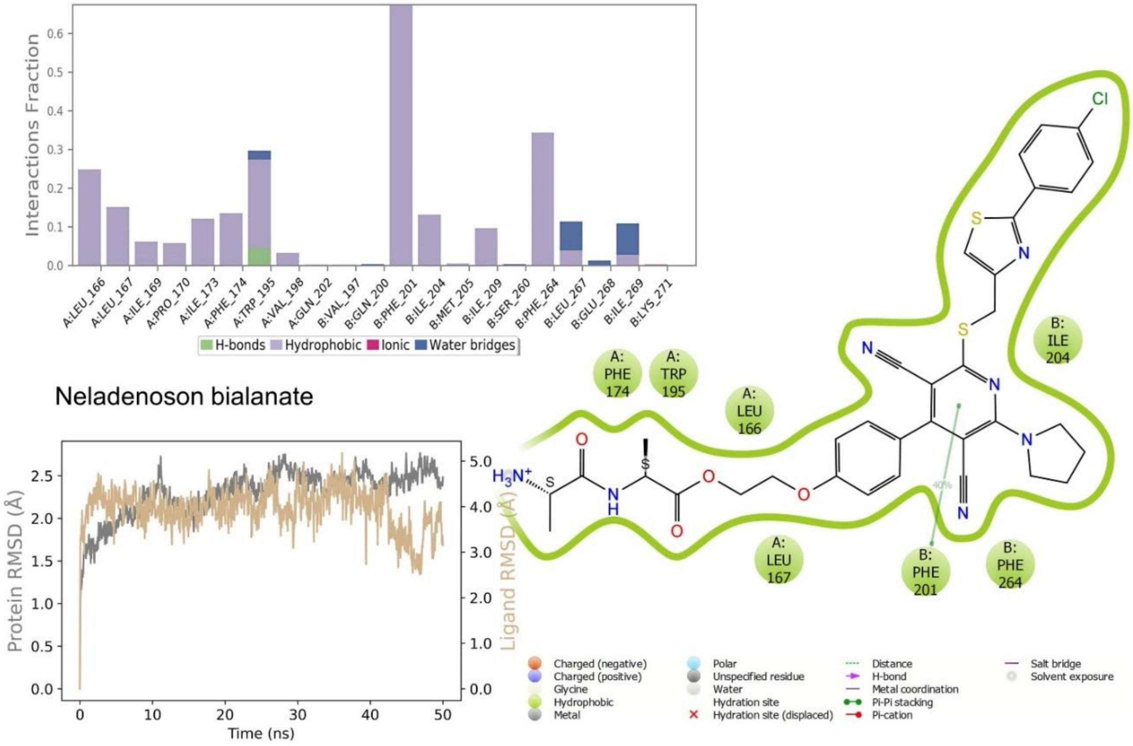

Figure S14. RMSD, ligand interaction diagrams and interaction fractions of Neladenoson bialanate docked in DA1.

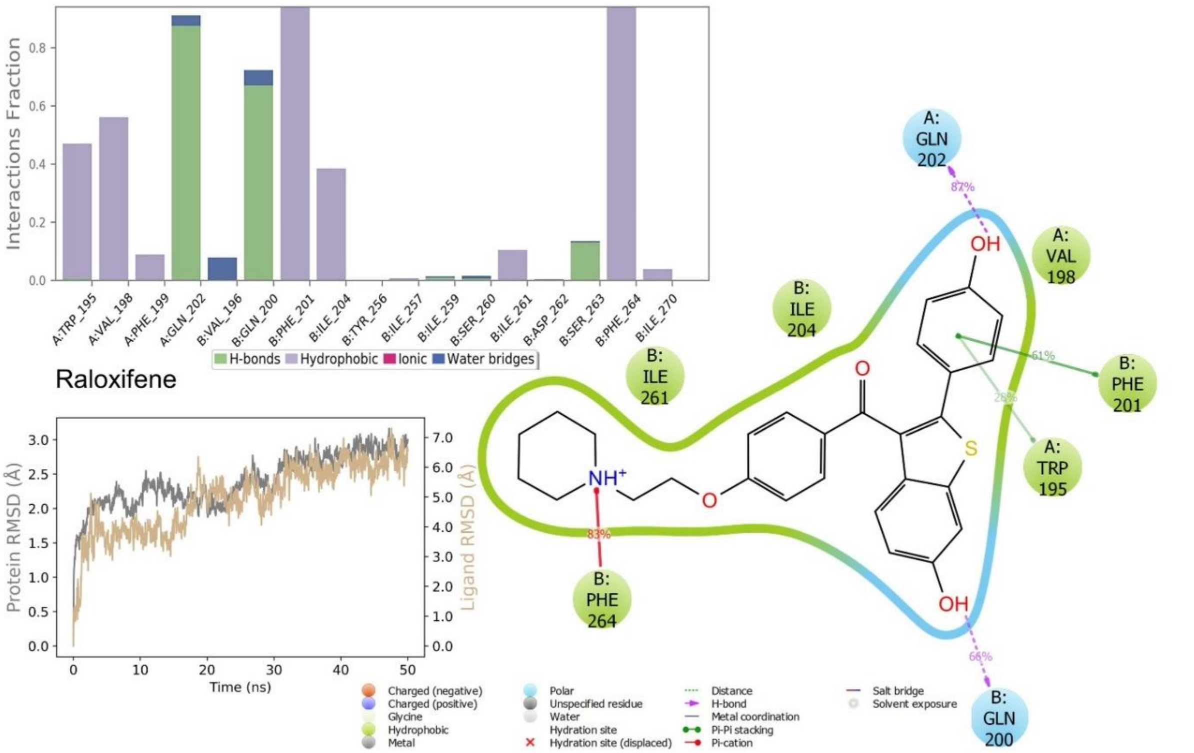

Figure S15. RMSD, ligand interaction diagrams and interaction fractions of Raloxifene docked in DA1.

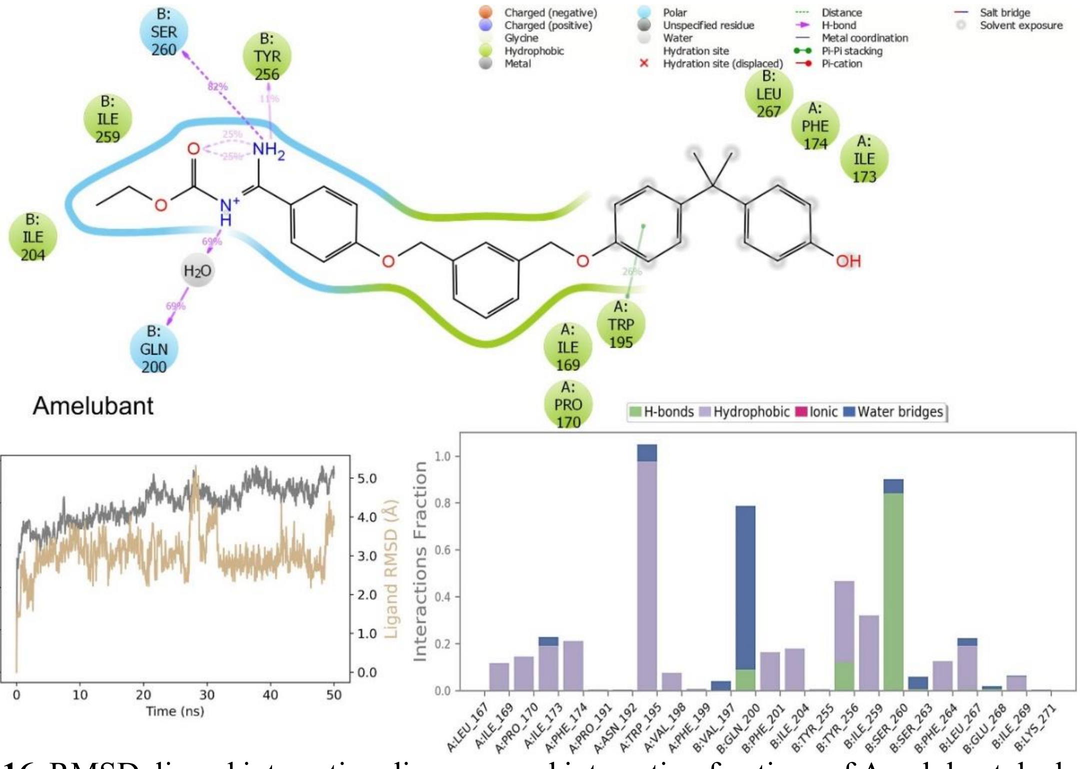

Figure S16. RMSD, ligand interaction diagrams and interaction fractions of Amelubant docked in DA1.

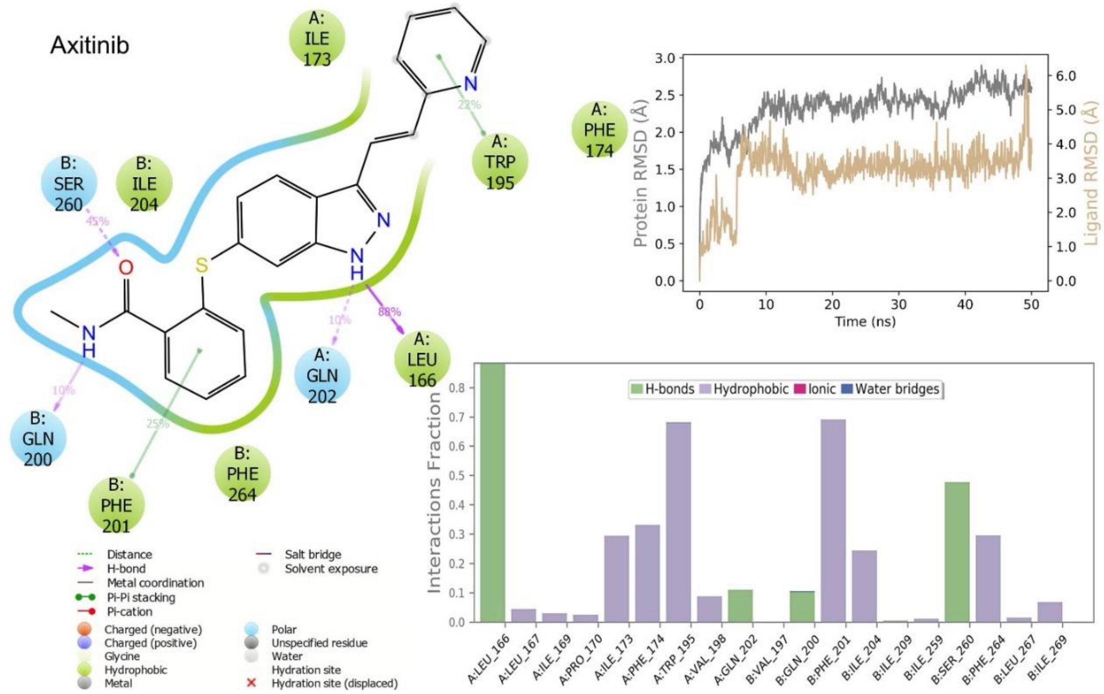

Figure S17. RMSD, ligand interaction diagrams and interaction fractions of Axitinib docked in DA1.

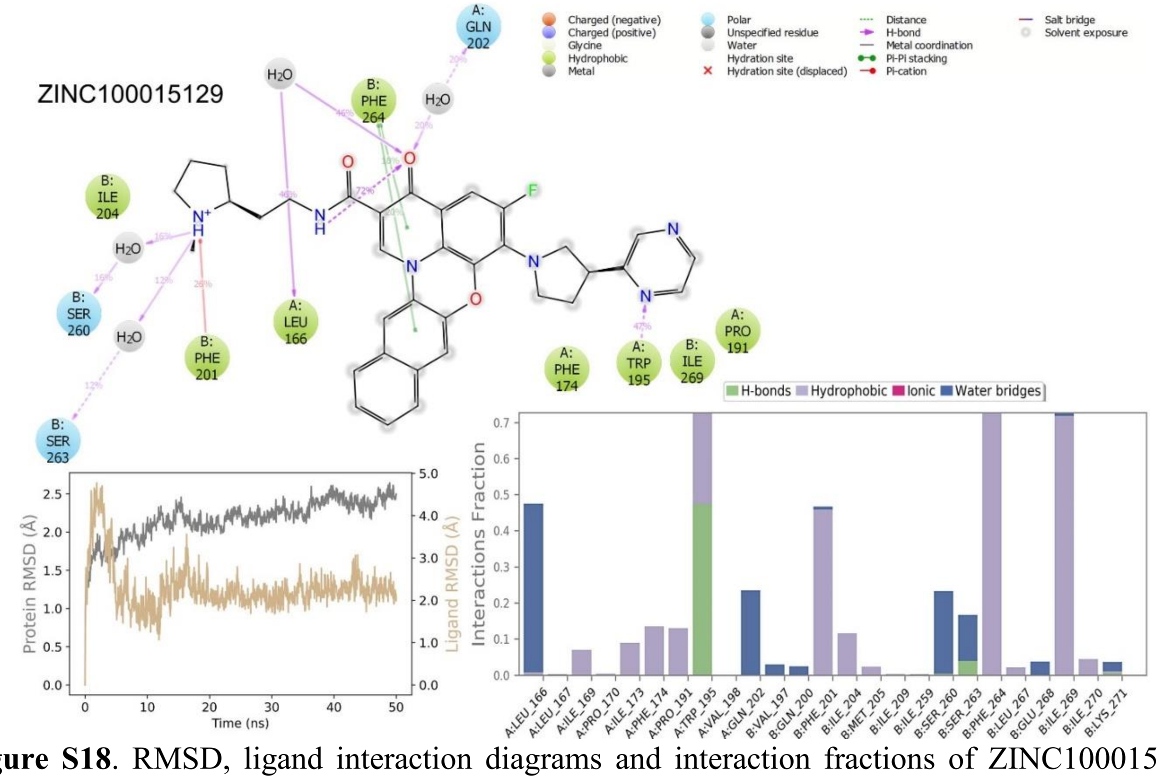

Figure S18. RMSD, ligand interaction diagrams and interaction fractions of ZINC100015129 docked in DA1.

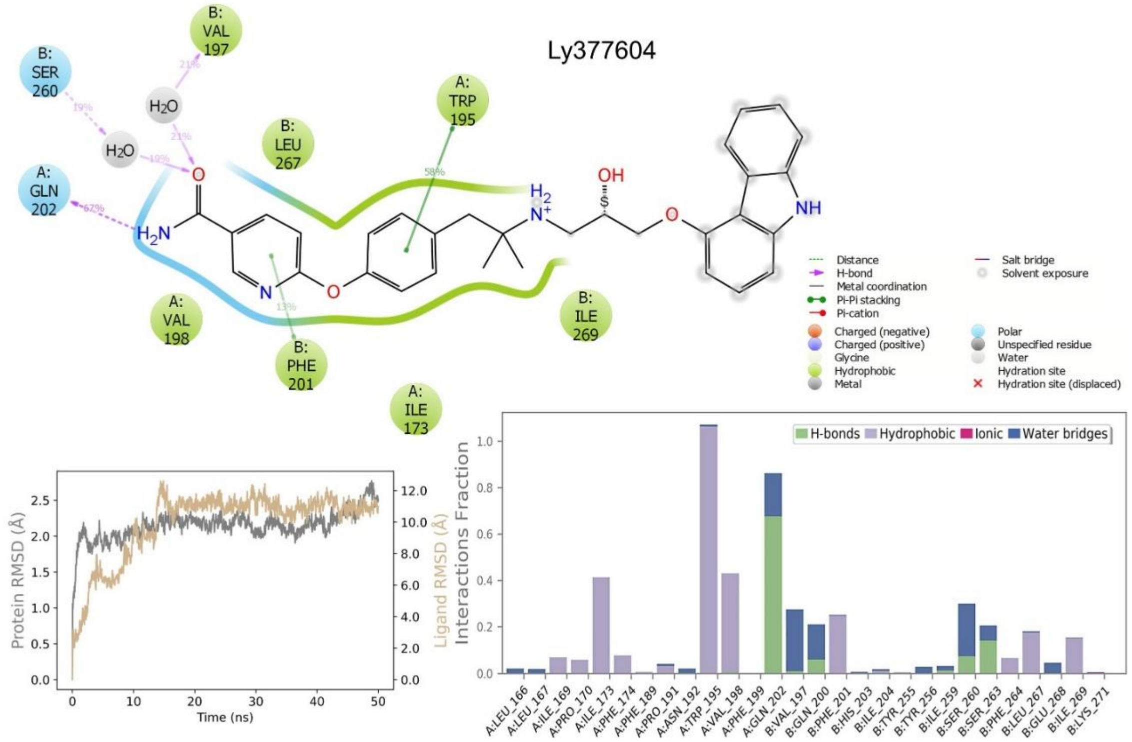

Figure S19. RMSD, ligand interaction diagrams and interaction fractions of Ly377604 docked in DA1.

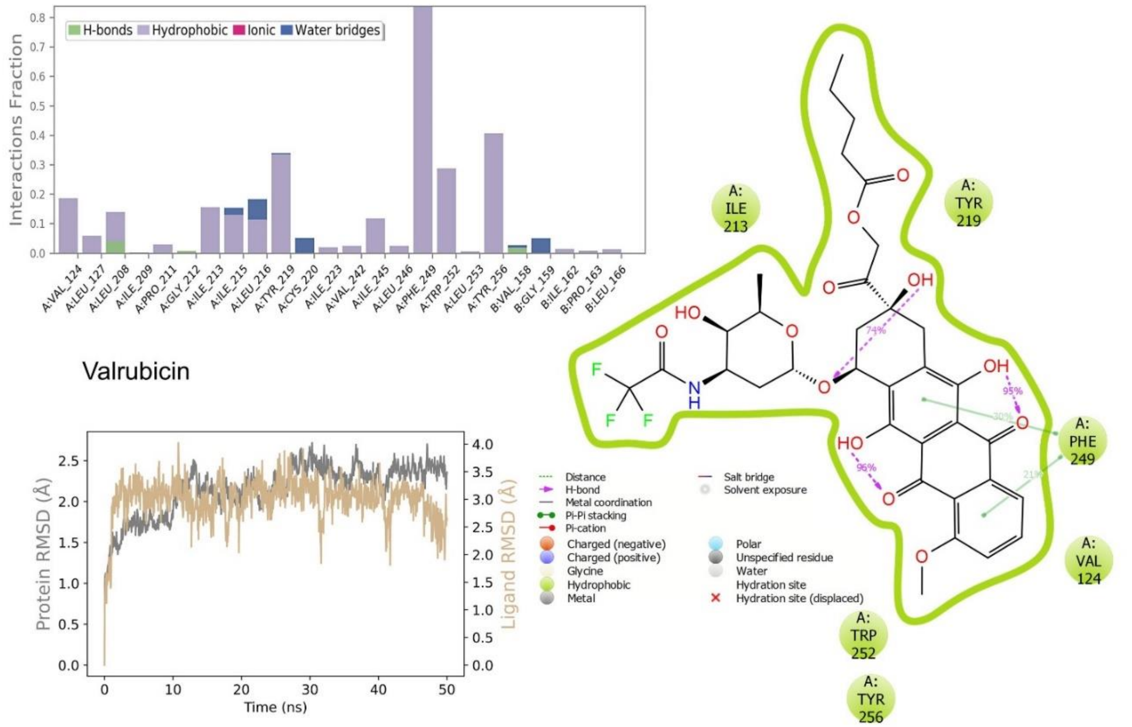

Figure S20. RMSD, ligand interaction diagrams and interaction fractions of Valrubicin docked in DA2.

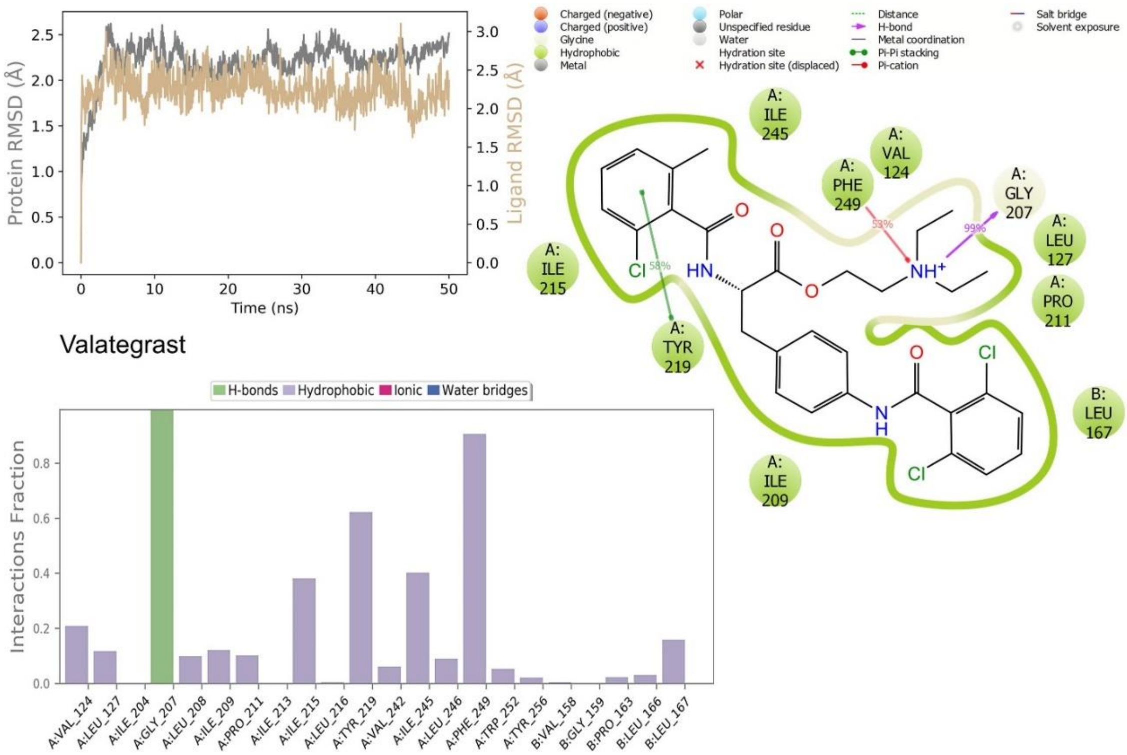

Figure S21. RMSD, ligand interaction diagrams and interaction fractions of Valategrast docked in DA2.

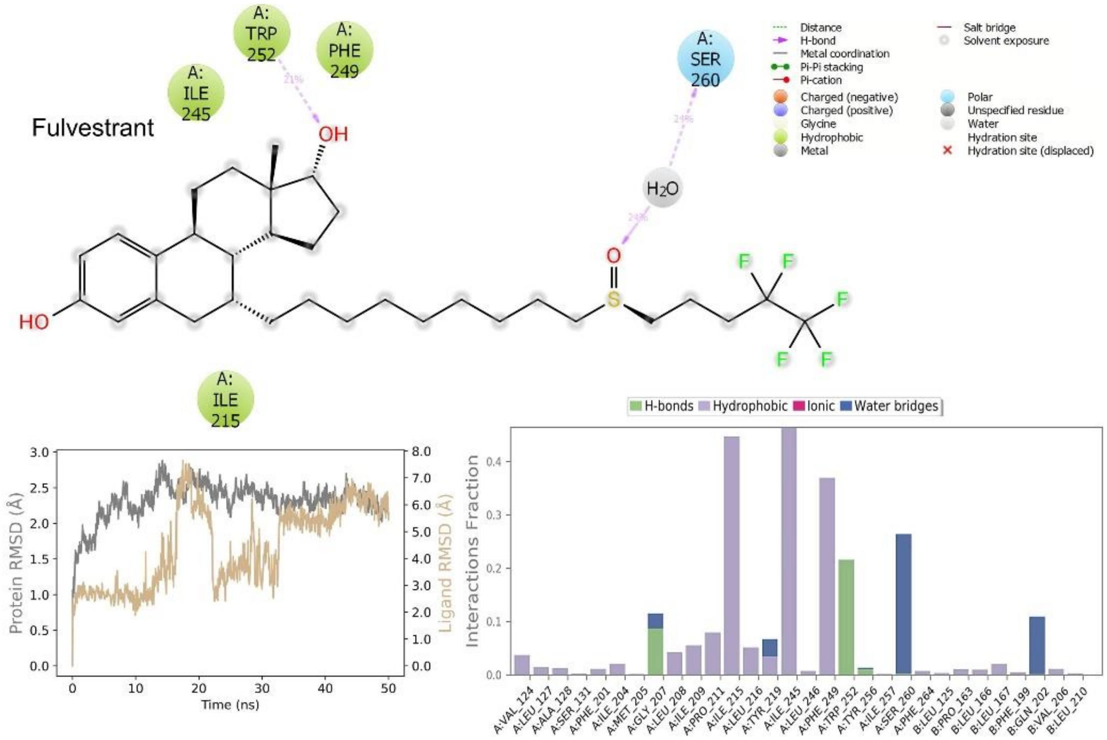

Figure S22. RMSD, ligand interaction diagrams and interaction fractions of Fulvestrant docked in DA2.

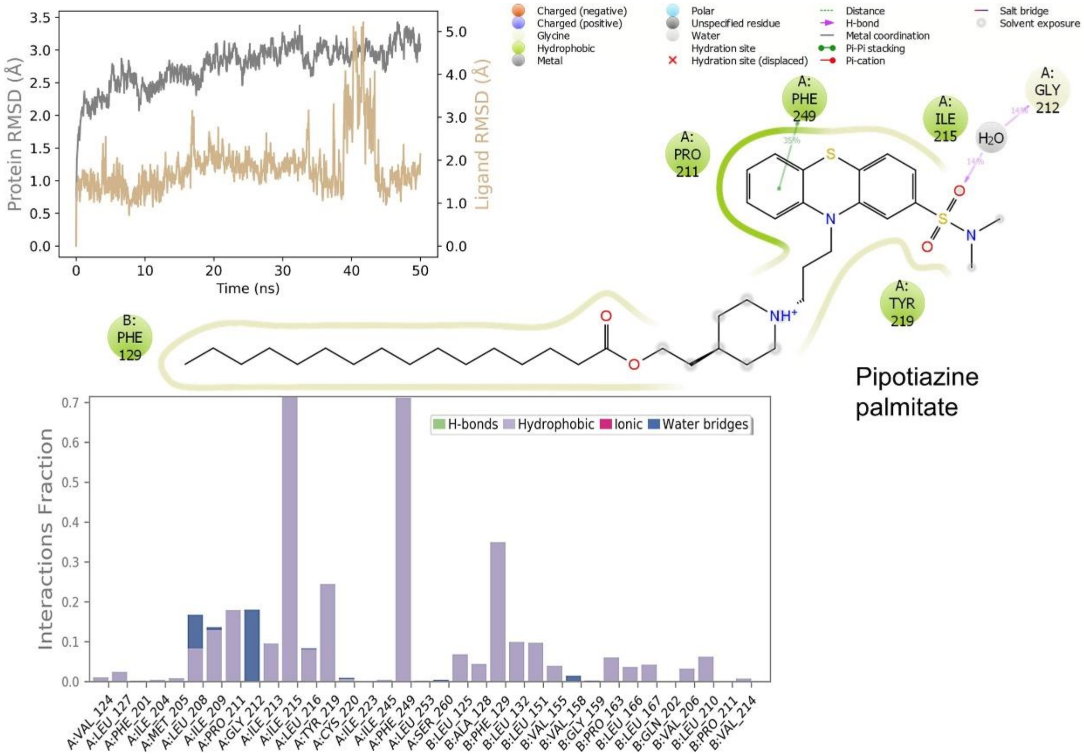

Figure S23. RMSD, ligand interaction diagrams and interaction fractions of Pipotiazine Palmitate docked in DA2.

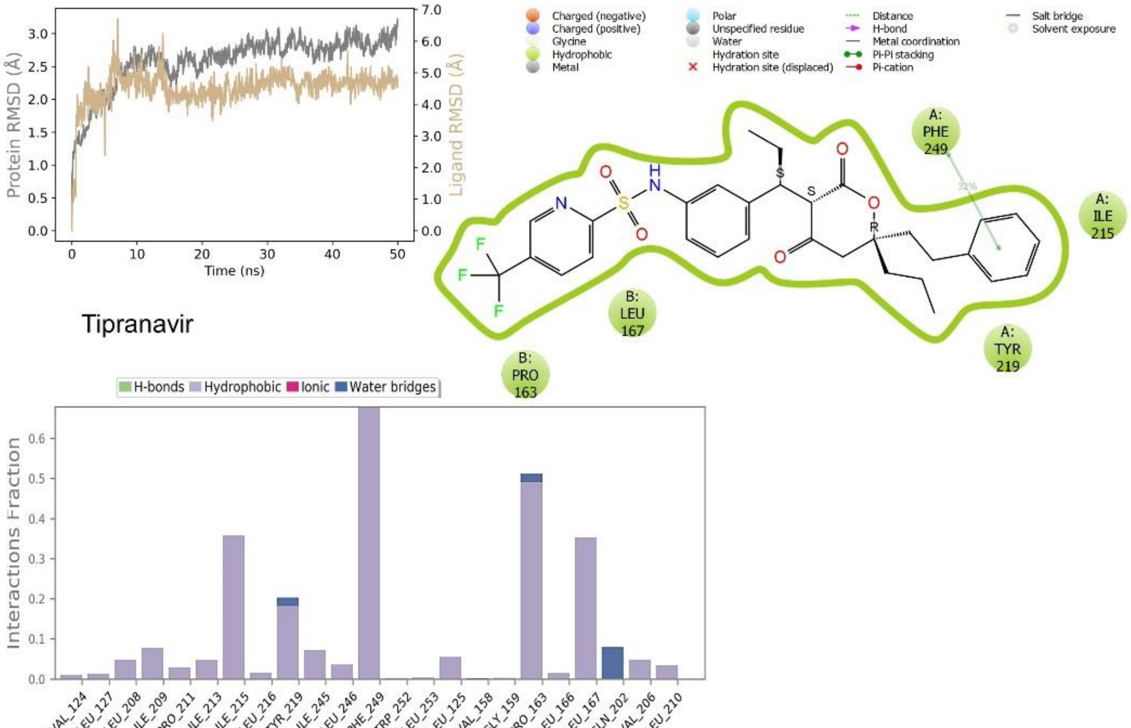

Figure S24. RMSD, ligand interaction diagrams and interaction fractions of Tipranavir docked in DA2.

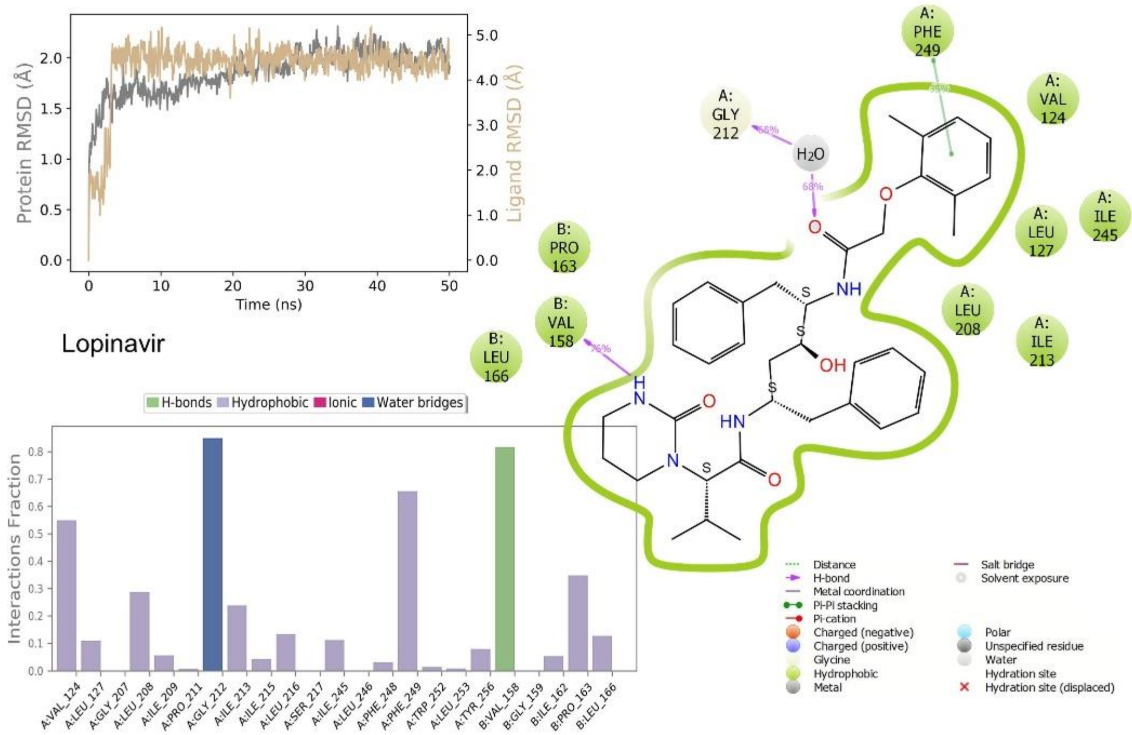

Figure S25. RMSD, ligand interaction diagrams and interaction fractions of Lopinavir docked in DA2.

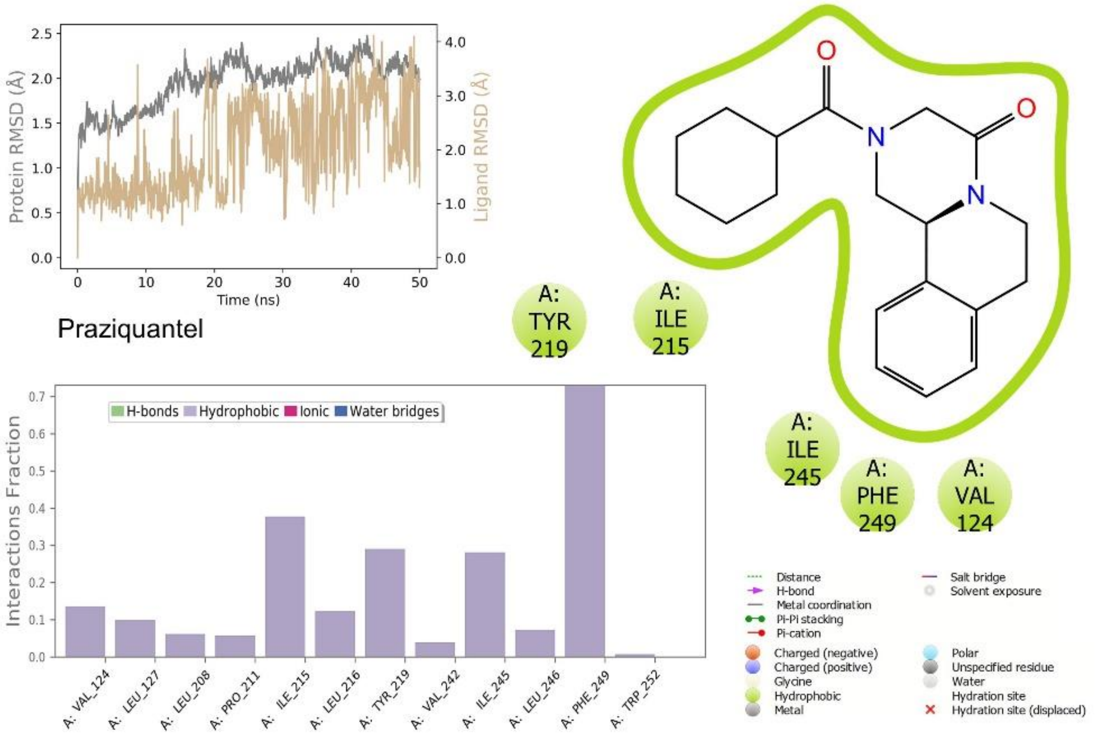

Figure S26. RMSD, ligand interaction diagrams and interaction fractions of Praziquantel docked in DA2.

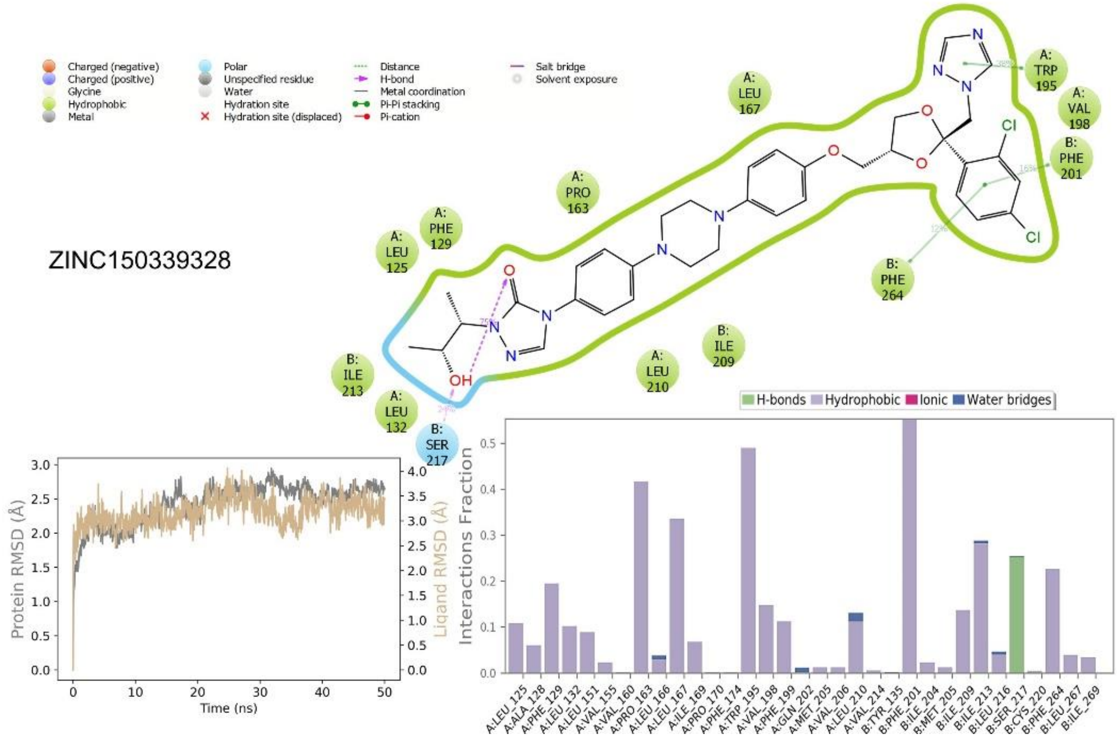

Figure S27. RMSD, ligand interaction diagrams and interaction fractions of ZINC150339328 docked in DA3.

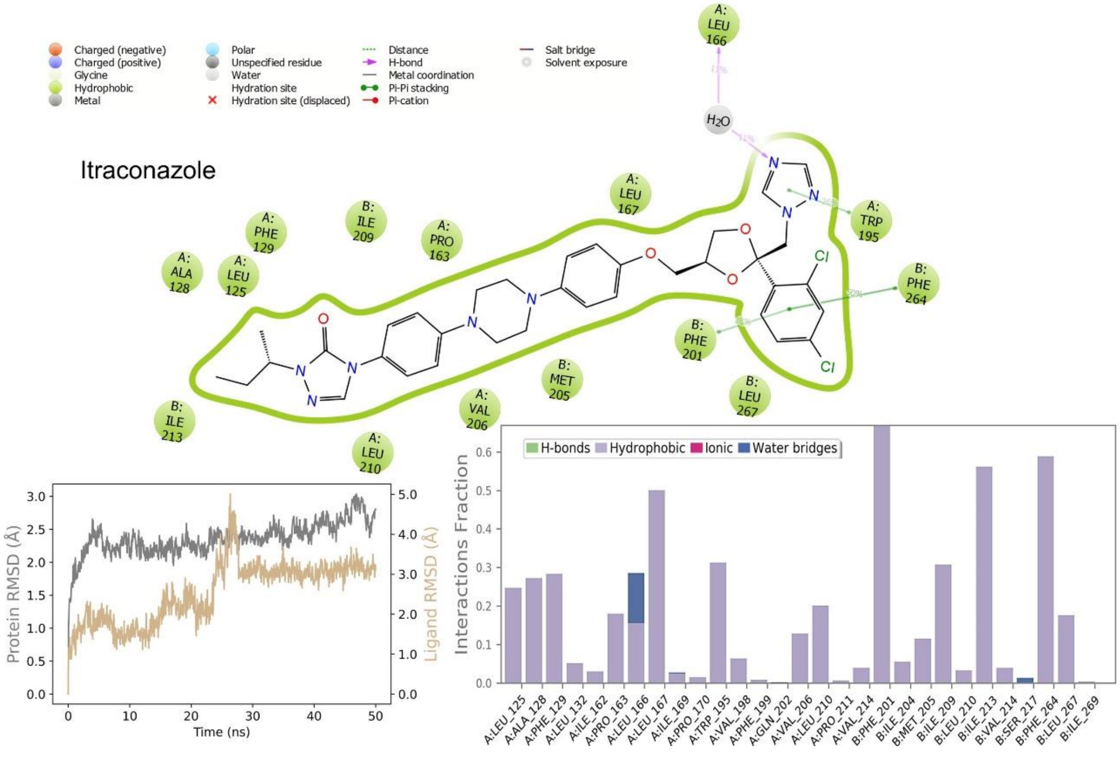

Figure S28. RMSD, ligand interaction diagrams and interaction fractions of Itraconazole docked in DA3.

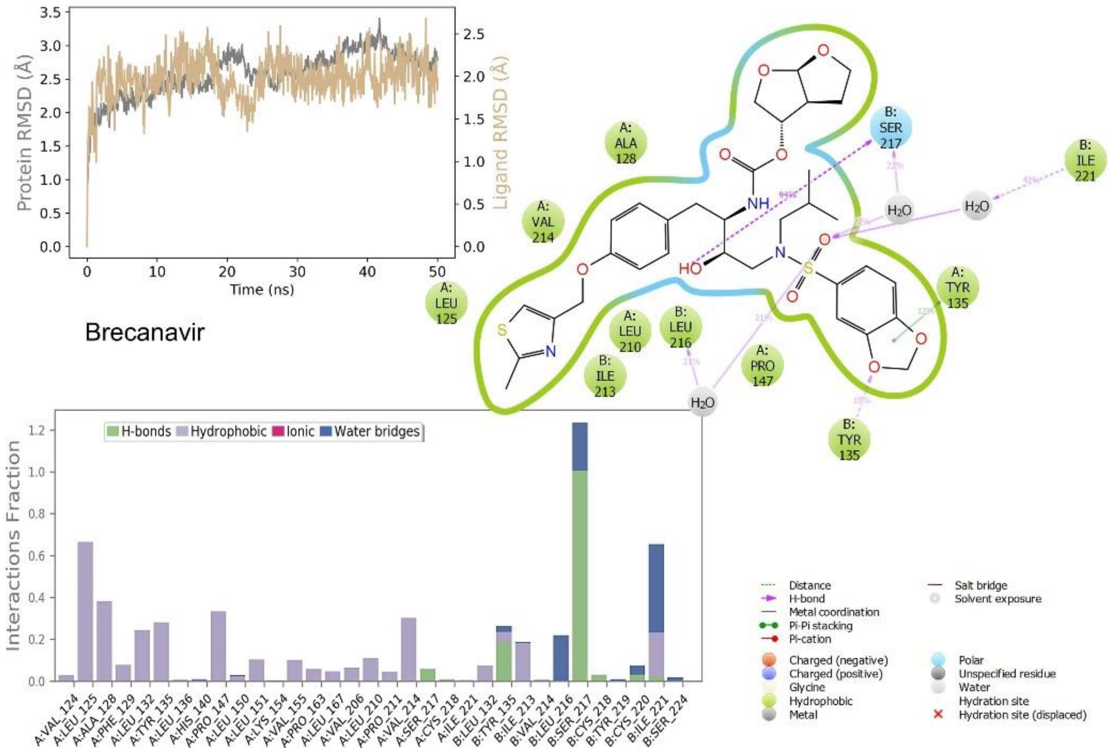

Figure S29. RMSD, ligand interaction diagrams and interaction fractions of Brecanavir docked in DA3.

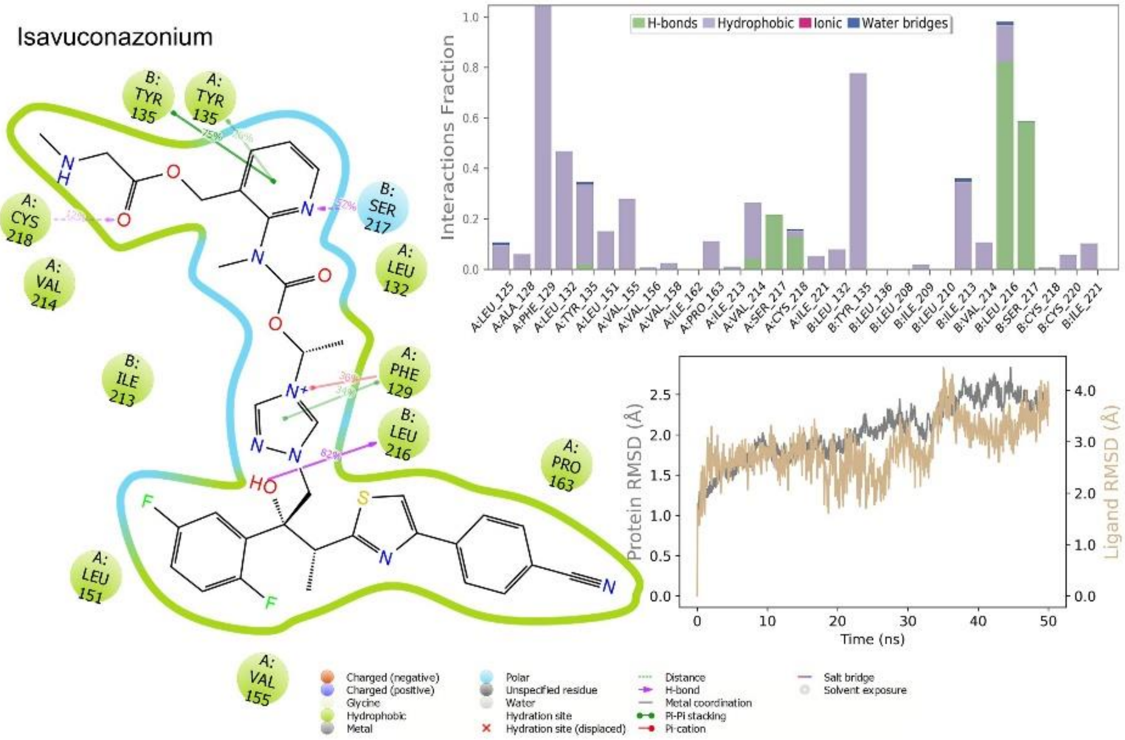

Figure S30. RMSD, ligand interaction diagrams and interaction fractions of Isavuconazonium docked in DA3.

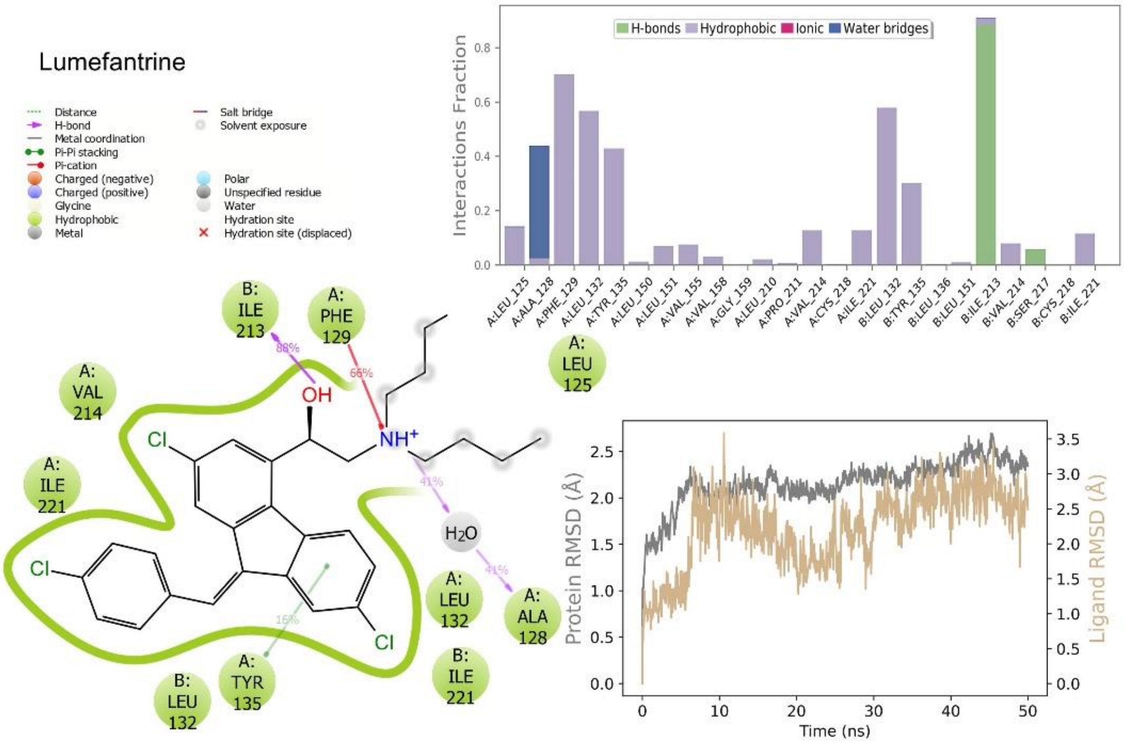

Figure S31. RMSD, ligand interaction diagrams and interaction fractions of Lumefantrine docked in DA3.

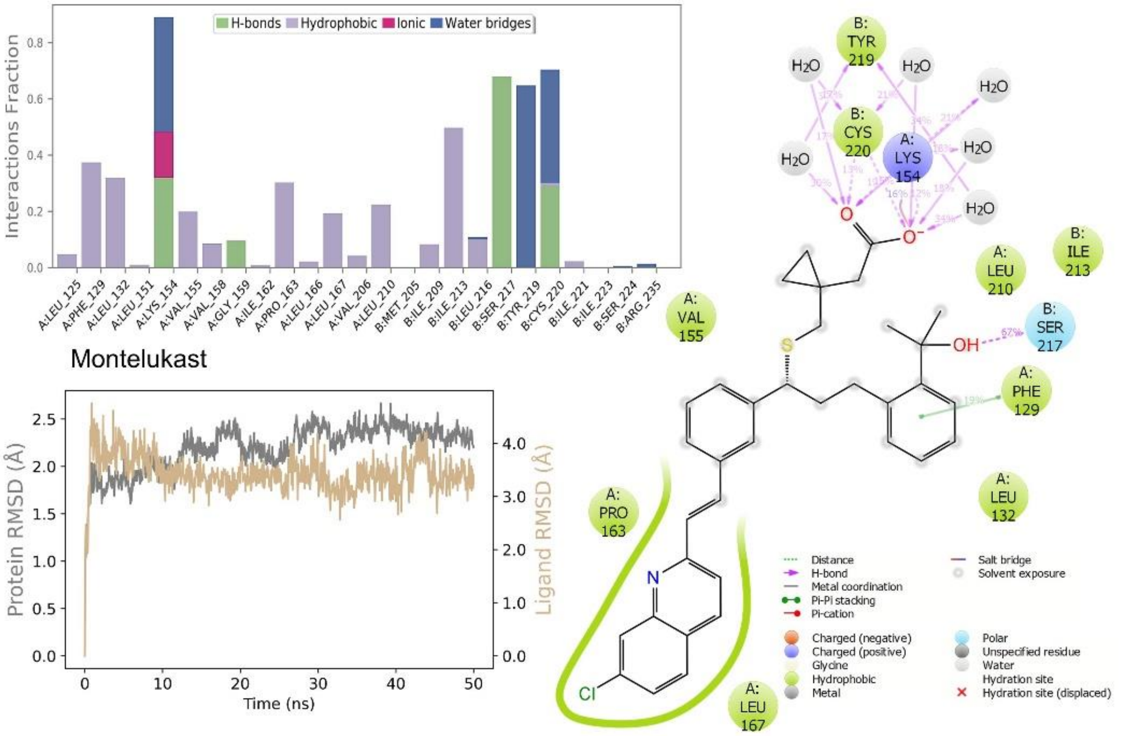

Figure S32. RMSD, ligand interaction diagrams and interaction fractions of Montelukast docked in DA3.

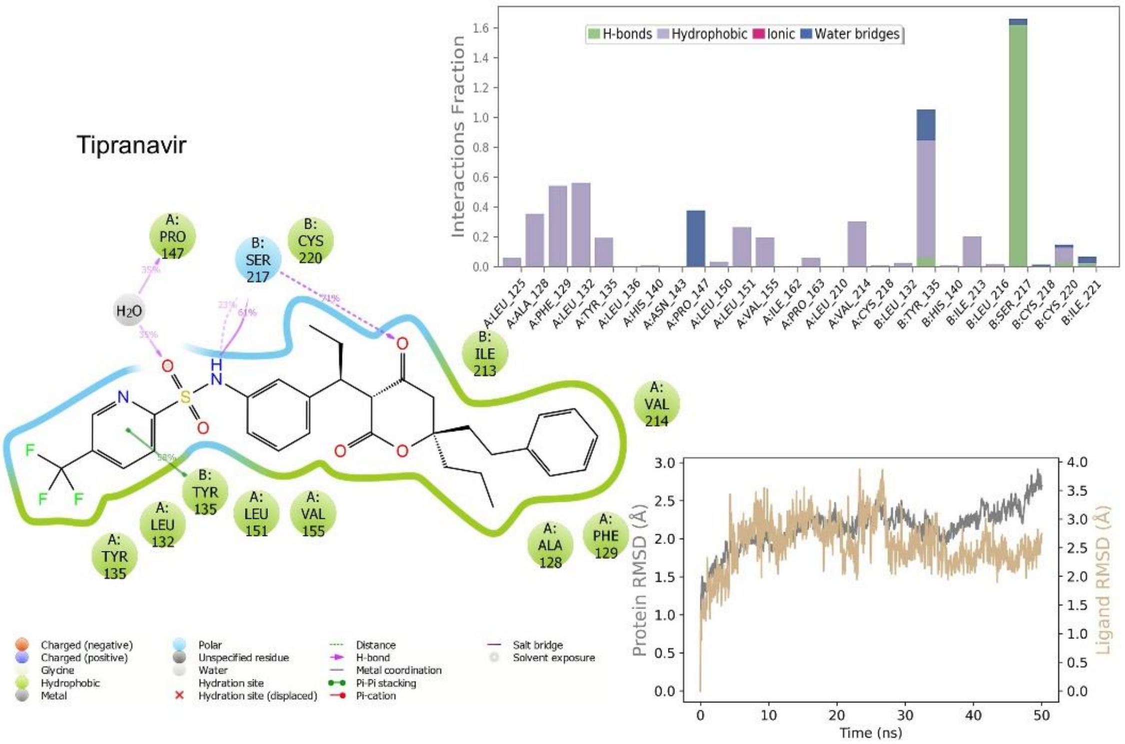

Figure S33. RMSD, ligand interaction diagrams and interaction fractions of Tipranavir docked in DA3.

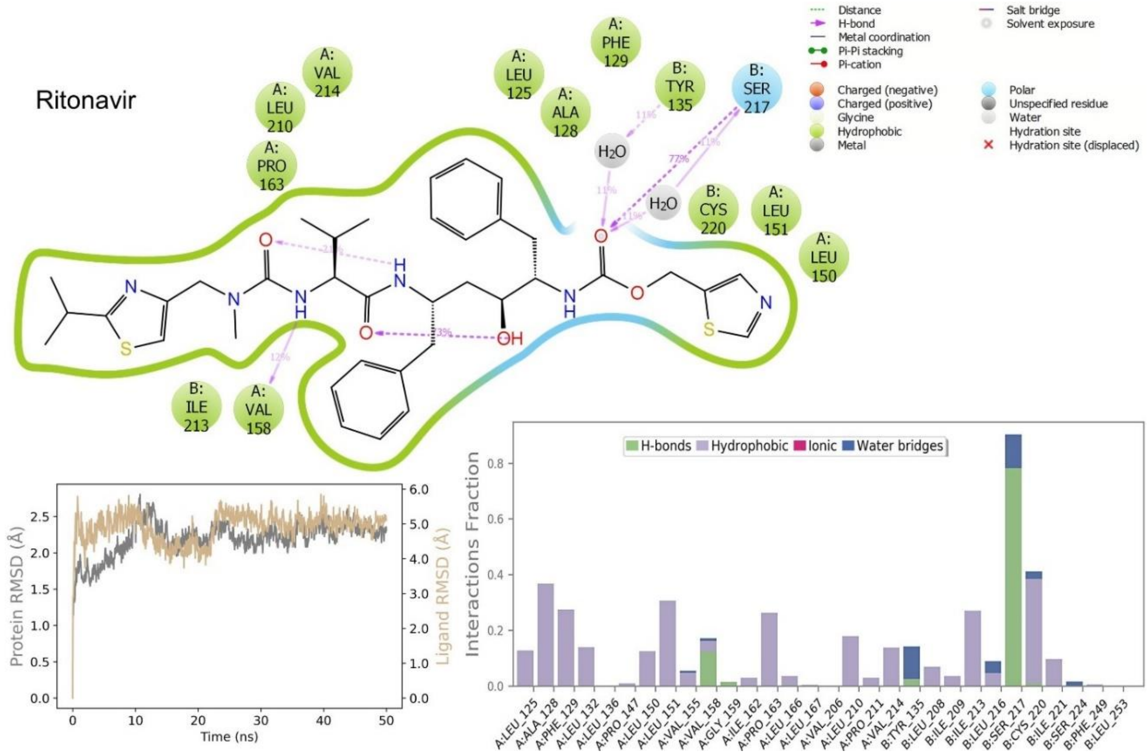

Figure S34. RMSD, ligand interaction diagrams and interaction fractions of Ritonavir docked in DA3.

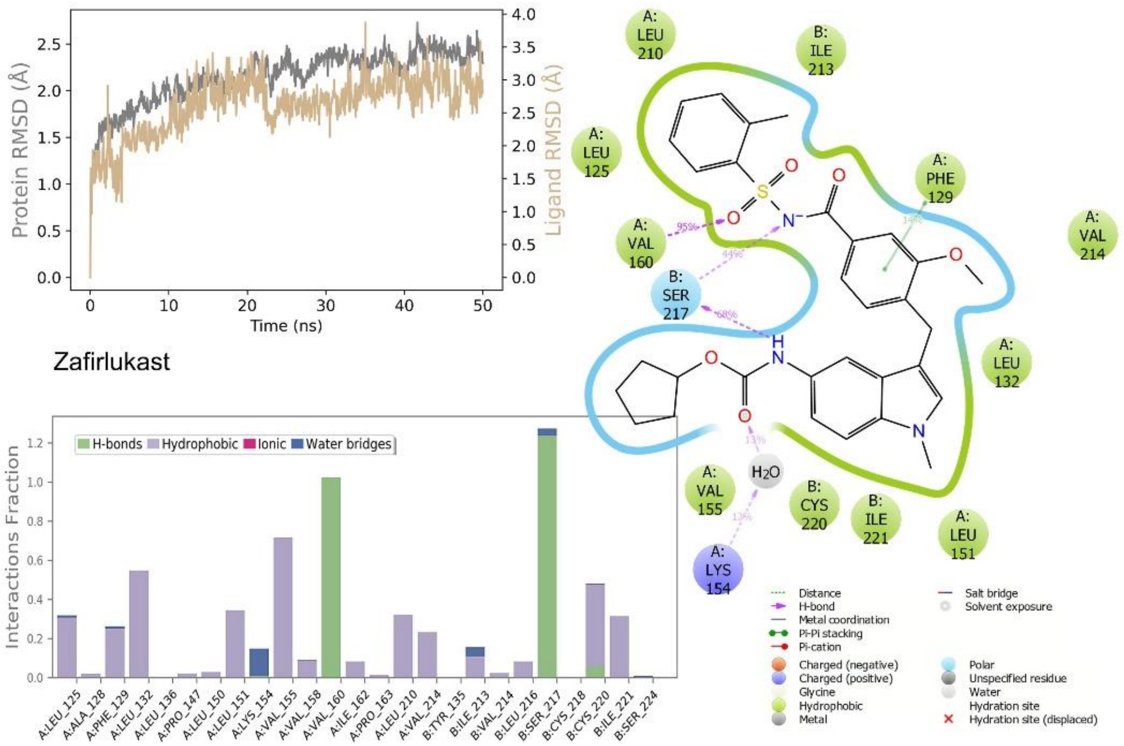

Figure S35. RMSD, ligand interaction diagrams and interaction fractions of Zafirlukast docked in DA3.

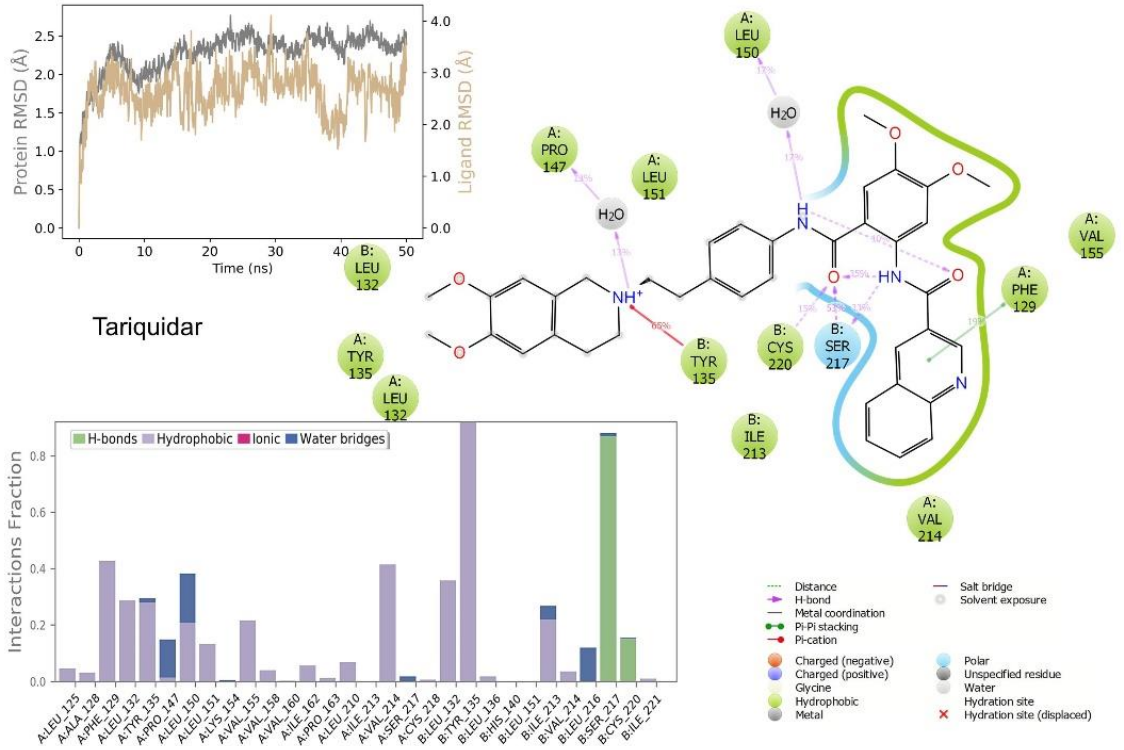

Figure S36. RMSD, ligand interaction diagrams and interaction fractions of Tariquidar docked in DA3.

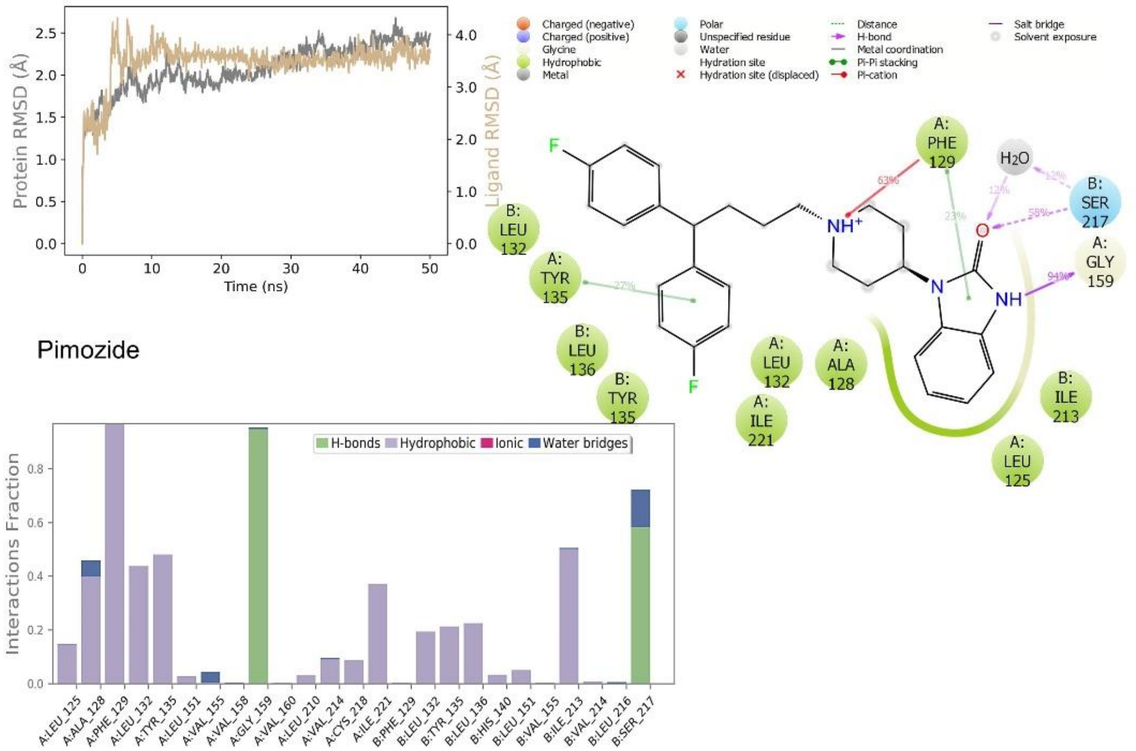

Figure S37. RMSD, ligand interaction diagrams and interaction fractions of Pimozide docked in DA3.

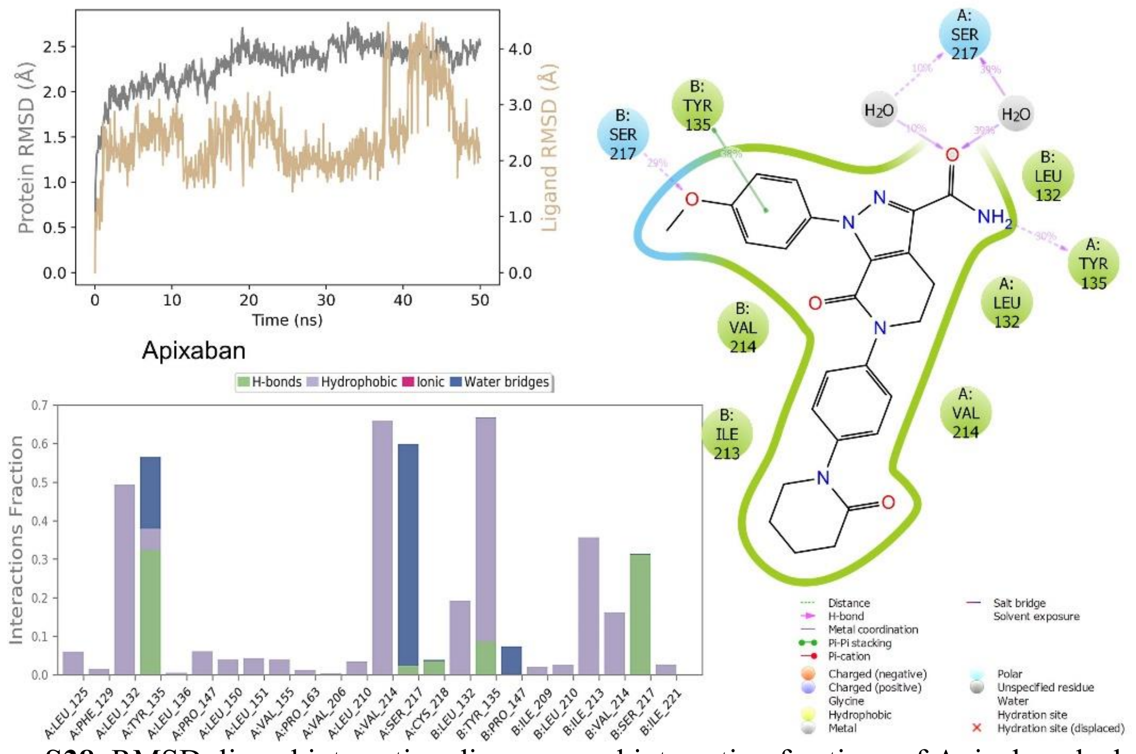

Figure S38. RMSD, ligand interaction diagrams and interaction fractions of Apixaban docked in DA3.

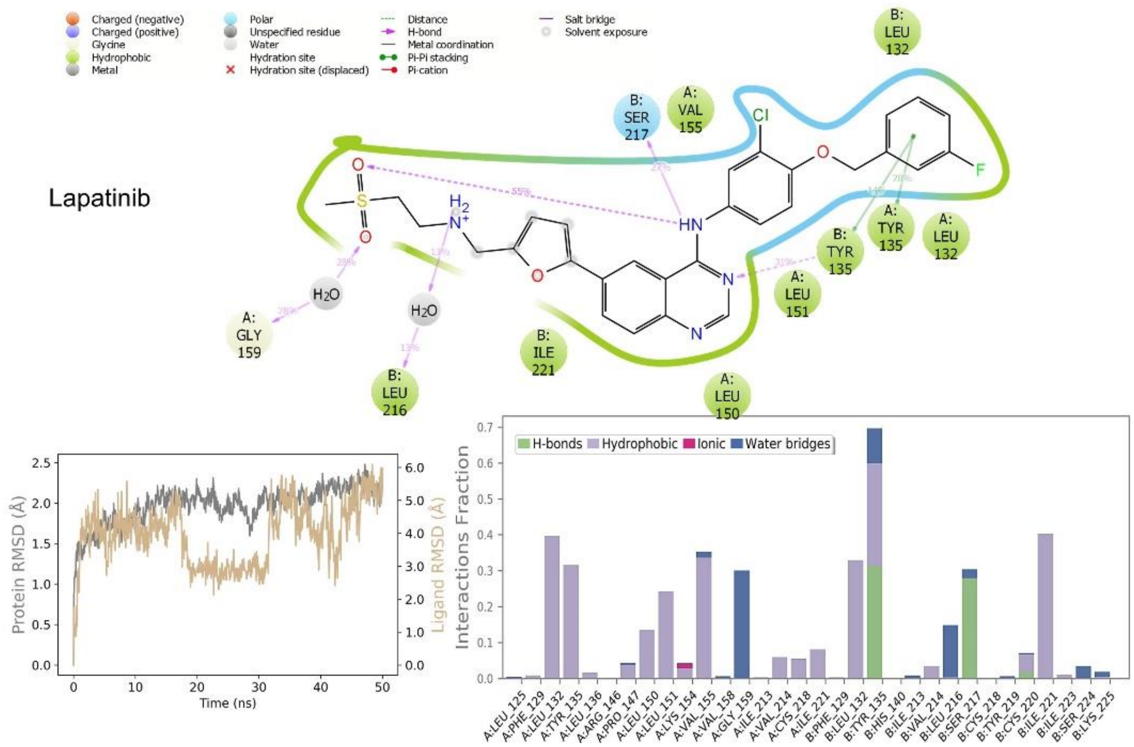

Figure S39. RMSD, ligand interaction diagrams and interaction fractions of Lapatinib docked in DA3.

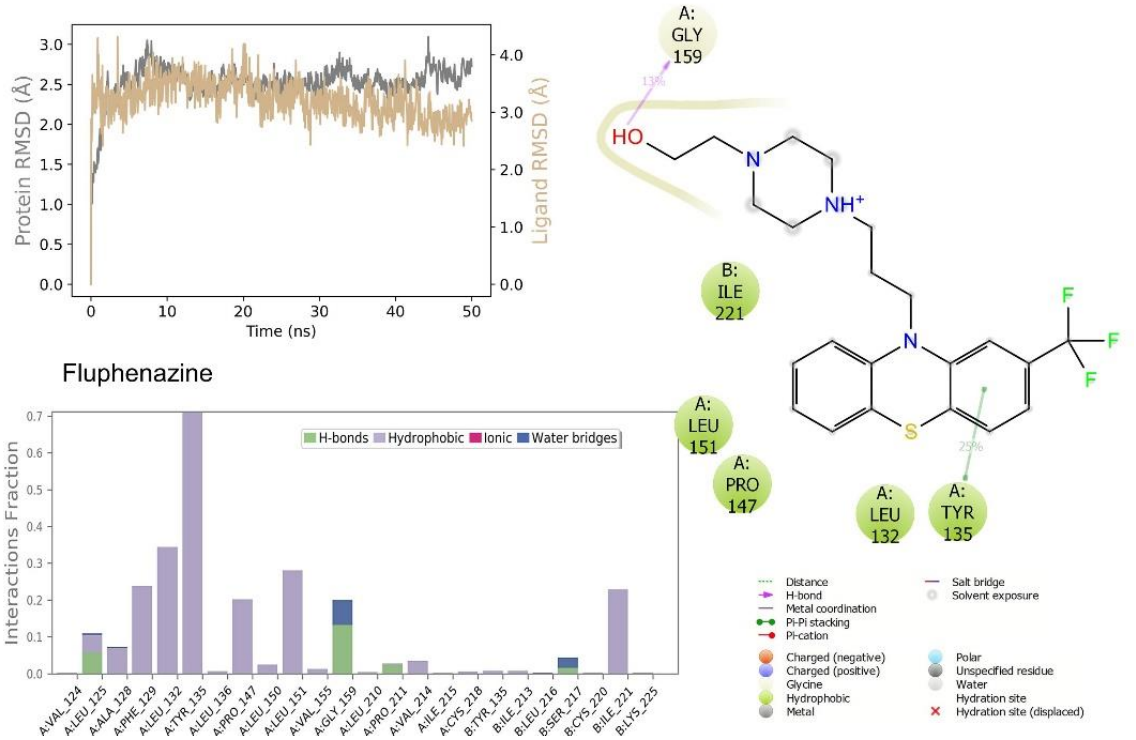

Figure S40. RMSD, ligand interaction diagrams and interaction fractions of Fluphenazine docked in DA3.

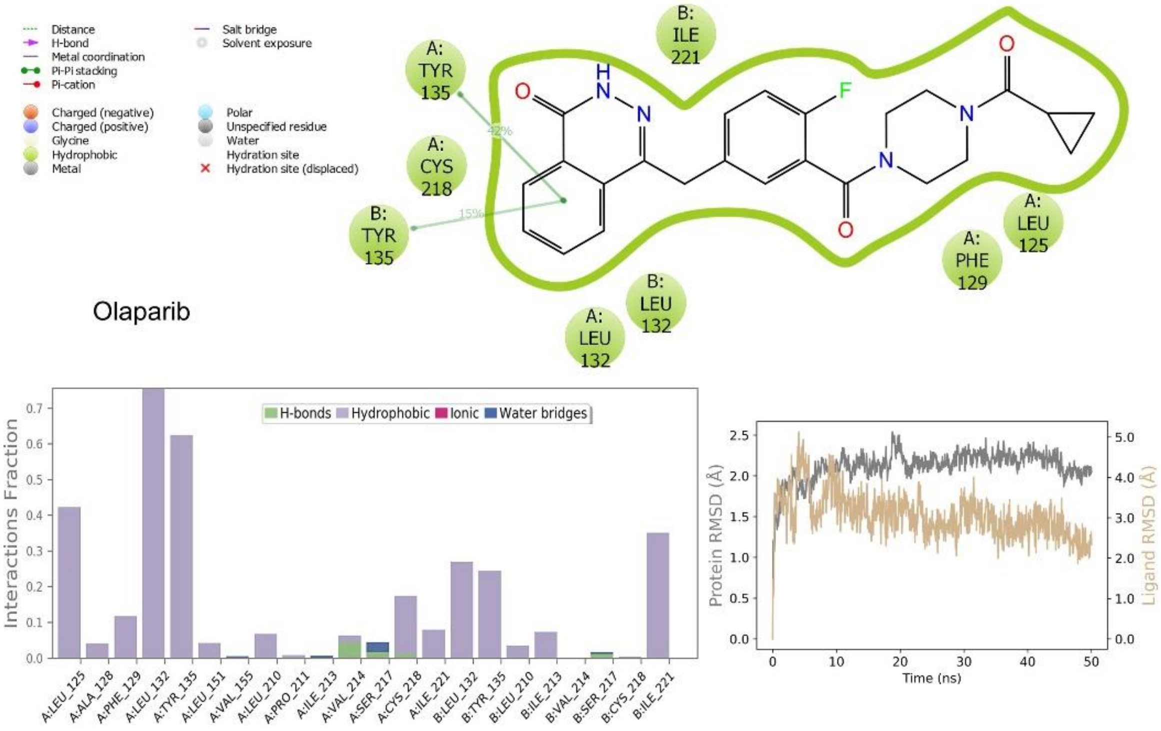

Figure S41. RMSD, ligand interaction diagrams and interaction fractions of Olaparib docked in DA3.

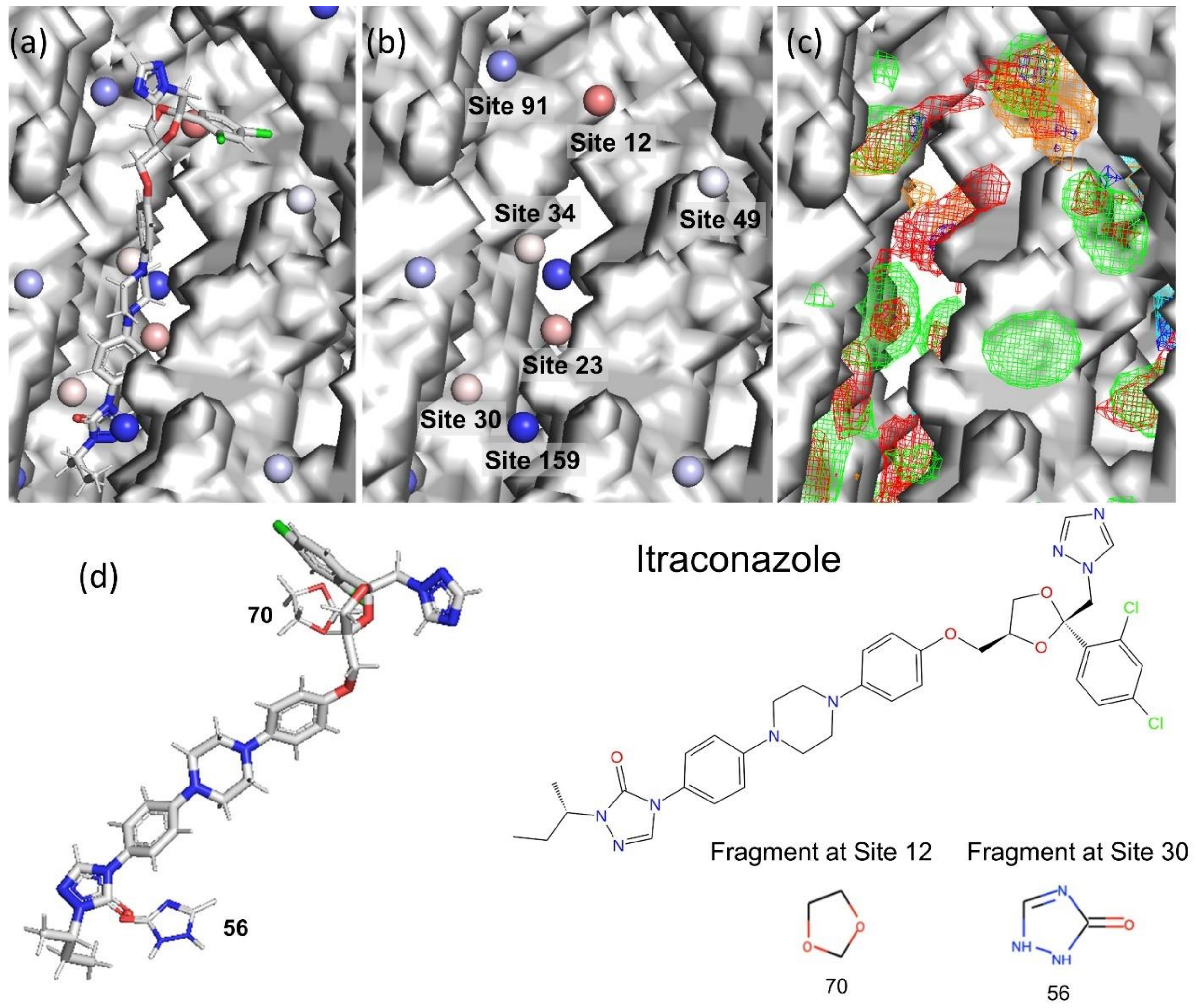

Figure S42. (a) Potential allosteric binding site DA3 of monomer CXCR4 with the hit compound Itraconazole. Hotspots (b) occupied pockets of interest with selected fragments, and FragMaps (c) are shown with the protein. (d) Selected fragments are shown with the docked ligand.

REFERENCES

(1) Saad, F.; Shore, N. D. Relugolix: A Novel Androgen Deprivation Therapy for Management of Patients with Advanced Prostate Cancer. *Therapeutic Advances in Medical Oncology* **2021,** *13*, 1758835921998586**.** DOI:10.1177/1758835921998586.

(2) McDaniel, N. K.; Fischbach, S. R.; Ondracek, O. J.; Welke, N. B.; Iida, M.; Wheeler, D. L. TAM Family Proteins and Therapy Resistance. *Improving the Therapeutic Ratio in Head and Neck Cancer* **2020**, 159–192. DOI:10.1016/B978-0-12-817868-3.00008-1.

(3) Blancas, I.; Olier, C.; Conde, V.; Bayo, J. L.; Herrero, C.; Zarcos-Pedrinaci, I.; Carabantes, F.; Baena-Cañada, J. M.; Cruz, J.; Ruiz-Borrego, M. Real-World Data of Fulvestrant as First-Line Treatment of Postmenopausal Women with Estrogen Receptor-Positive Metastatic Breast Cancer. *Sci Rep* **2021**, *11* (1), 4274. DOI:10.1038/s41598-021-83622-1.

(4) FDA Briefing Document, Psychopharmacologic Drugs Advisory Committee. *Pimavanserin For The Treatment Of Hallucinations And Delusions Associated With Alzheimer's Disease Psychosis.* Psychopharmacologic Drugs Advisory Committee. June 17*,* 2022.

(5) Kelly, M. A. Neurological Complications of Cardiovascular Drugs. *Handb Clin Neurol* **2021**, *177*, 319–344. DOI:10.1016/B978-0-12-819814-8.00020-2.

(6) Angiolillo, D. J.; Rollini, F.; Storey, R. F.; Bhatt, D. L.; James, S.; Schneider, D. J.; Sibbing, D.; So, D. Y. F.; Trenk, D.; Alexopoulos, D.; Gurbel, P. A.; Hochholzer, W.; de Luca, L.; Bonello, L.; Aradi, D.; Cuisset, T.; Tantry, U. S.; Wang, T. Y.; Valgimigli, M.; Waksman, R.; Mehran, R.; Montalescot, G.; Franchi, F.; Price, M. J. International Expert Consensus on Switching Platelet P2Y12 Receptor-Inhibiting Therapies. *Circulation* **2017**, *136* (20), 1955–1975. DOI:10.1161/CIRCULATIONAHA.117.031164.

(7) Goudar, R. K. Review of Pemetrexed in Combination with Cisplatin for the Treatment of Malignant Pleural Mesothelioma. *Therapeutics and clinical risk management* **2008,** *4* (1), 205–211. DOI:10.2147/tcrm.s1603

(8) Voors, A. A.; Bax, J. J.; Hernandez, A. F.; Wirtz, A. B.; Pap, A. F.; Ferreira, A. C.; Senni, M.; van der Laan, M.; Butler, J. Safety and Efficacy of the Partial Adenosine A1 Receptor Agonist Neladenoson Bialanate in Patients with Chronic Heart Failure with Reduced Ejection Fraction: A Phase IIb, Randomized, Double-Blind, Placebo-Controlled Trial. *European Journal of Heart Failure* **2019**, *21*, 1426–1433. DOI:10.1002/ejhf.1591.

(9) Hurvitz, S. A.; Kakkar, R. Role of Lapatinib Alone or in Combination in the Treatment of HER2-Positive Breast Cancer. *Breast cancer (Dove Medical Press)* **2012**, *4*, 35–51. DOI:10.2147/BCTT.S29996.

(10) de Luca, A.; D'alessio, A.; Gallo, M.; Maiello, M.; Bode, A.; Normanno, N. Cell Cycle Src and CXCR4 Are Involved in the Invasiveness of Breast Cancer Cells with Acquired Resistance to Lapatinib. *Cell Cycle* **2014**, *13* (1), 148–156. DOI:10.4161/cc.26899.

(11) Jesudason, C. D.; Baker, J. E.; Bryant, R. D.; Fisher, J. W.; O'farrell, L. S.; Gaich, G. A.; He, M. M.; Kahl, S. D.; Kriauciunas, A. v; Heiman, M. L.; Peters, M. A.; Rito, C. J.; Satterwhite, J. H.; Tinsley, F. C.; Trankle, W. G.; Shuker, A. J. Combination of a Beta Adrenoceptor Modulator and a Norepinephrine-Serotonin Uptake Inhibitor for the Treatment of Obesity. *ACS Med. Chem. Lett.* **2011**, *2*, 583–586. DOI:10.1021/ml200071k.

(12) Bhatt, L.; Roinestad, K.; Van, T.; Springman, E. B. Recent Advances in Clinical Development of Leukotriene B4 Pathway Drugs. *Seminars in Immunology* **2017**, *33*, 65–73. DOI:10.1016/J.SMIM.2017.08.007.

(13) Wishart, D. S., Feunang, Y. D., Guo, A. C., Lo, E. J., Marcu, A., Grant, J. R., Sajed, T., Johnson, D., Li, C., Sayeeda, Z., Assempour, N., Iynkkaran, I., Liu, Y., Maciejewski, A., Gale, N., Wilson, A., Chin, L., Cummings, R., Le, D., Pon, A., … Wilson, M. DrugBank 5.0: a major update to the DrugBank database for 2018. *Nucleic acids research*, **2018**, 46 (D1), D1074–D1082. DOI:10.1093/nar/gkx1037.

(14) Hu-Lowe, D. D.; Zou, H. Y.; Grazzini, M. L.; Hallin, M. E.; Wickman, G. R.; Amundson, K.; Chen, J. H.; Rewolinski, D. A.; Yamazaki, S.; Wu, E. Y.; McTigue, M. A.; Murray, B. W.; Kania, R. S.; O'Connor, P.; Shalinsky, D. R.; Bender, S. L. Nonclinical Antiangiogenesis and Antitumor Activities of Axitinib (AG-013736), an Oral, Potent, and Selective Inhibitor of Vascular Endothelial Growth Factor Receptor Tyrosine Kinases 1, 2, 3. *Clinical Cancer Research* **2008**, *14* (22), 7272–7283. DOI:10.1158/1078-0432.CCR-08-0652.

(15) Bellesoeur, A., Carton, E., Alexandre, J., Goldwasser, F., & Huillard, O. Axitinib in the treatment of renal cell carcinoma: design, development, and place in therapy. *Drug design, development and therapy* **2017**, *11*, 2801–2811 DOI:10.2147/DDDT.S109640.

(16) Vogel, V. G.; Costantino, J. P.; Wickerham, D. L.; Cronin, W. M.; Cecchini, R. S.; Atkins, J. N.; Bevers, T. B.; Fehrenbacher, L.; Pajon, E. R.; Wade, J. L.; Robidoux, A.; Margolese, R. G.; James, J.; Runowicz, C. D.; Ganz, P. A.; Reis, S. E.; McCaskill-Stevens, W.; Ford, L. G.; Jordan, V. C.; Wolmark, N. Update of the National Surgical Adjuvant Breast and Bowel Project Study of Tamoxifen and Raloxifene (STAR) P-2 Trial: Preventing Breast Cancer. *Cancer Prevention Research* **2010**, *3* (6), 696–706. DOI:10.1158/1940-6207.CAPR-10-0076.

(17) Nathan, M. R.; Schmid, P. A Review of Fulvestrant in Breast Cancer. *Oncology and Therapy* **2017**, *5* (1), 17–29. DOI:10.1007/s40487-017-0046-2.

(18) Dinesh, M.; David, A.; Quraishi, S. N. Depot pipotiazine palmitate and undecylenate for schizophrenia. *Cochrane Database of Systematic Reviews* **2004**, *3*, CD001720. DOI:10.1002/14651858.CD001720.pub2.

(19) Reddy, Y. S.; Ford, S. L.; Anderson, M. T.; Murray, S. C.; Ng-Cashin, J.; Johnson, M. A. Safety and Pharmacokinetics of Brecanavir, a Novel Human Immunodeficiency Virus Type 1 Protease Inhibitor, Following Repeat Administration with and without Ritonavir in Healthy Adult Subjects. *Antimicrobial Agents and Chemotherapy* **2007**, *51* (4), 1202–1208. DOI:10.1128/AAC.01005-06.

(20) Kelly, R. J.; Draper, D.; Chen, C. C.; Robey, R. W.; Figg, W. D.; Piekarz, R. L.; Chen, X.; Gardner, E. R.; Balis, F. M.; Venkatesan, A. M.; Steinberg, S. M.; Fojo, T.; Bates, S. E. A Pharmacodynamic Study of Docetaxel in Combination with the P-Glycoprotein Antagonist Tariquidar (XR9576) in Patients with Lung, Ovarian, and Cervical Cancer. *Clinical Cancer Research* **2011**, *17* (3), 569–580. DOI:10.1158/1078-0432.CCR-10-1725.

(21) Church, M. K.; Casale, T. B. Principles of Pharmacotherapy. *Allergy* **2012**, 147–169. DOI:10.1016/B978-0-7234-3658-4.00011-1.

(22) Shoham, S.; Groll, A. H.; Petraitis, V.; Walsh, T. J. Systemic Antifungal Agents. *Infectious Diseases* **2017**, 1333-1344.e4. DOI:10.1016/B978-0-7020-6285-8.00156-8.

(23) Eddy, C. M.; Rickards, H. E.; Cavanna, A. E. Treatment Strategies for Tics in Tourette Syndrome, *Therapeutic advances in neurological disorders* **2011**, *4* (1), 25–45. . DOI:10.1177/1756285610390261.

(24) Lam, S. Apixaban: A New Factor Xa Inhibitor for Stroke Prevention in Patients with Nonvalvular Atrial Fibrillation. *Cardiology in Review* **2013**, *21* (4), 207–212. DOI:10.1097/CRD.0b013e318293d6e6.

(25) Atarashi, H.; Kuruma, A.; Yashima, M. A.; Saitoh, H.; Ino, T.; Endoh, Y.; Hayakawa, H. Pharmacokinetics of Landiolol Hydrochloride, a New Ultra-Short-Acting β-Blocker, in Patients with Cardiac Arrhythmias. *Clin. Pharmacol. Ther.* **2000**, *68* (2), 143–150. DOI:10.1067/mcp.2000.108733.

(26) Whitson, J.T. Travoprost - A new prostaglandin analogue for the treatment of glaucoma. *Expert Opinion on Pharmacotherapy* **2002,** *3* (7), 965-977. DOI:10.1517/14656566.3.7.965

(27) Allison, A. C.; Eugui, E. M. Mycophenolate mofetil and its mechanisms of action. *Immunopharmacology* **2000**, *47* (2-3), 85–118.DOI:10.1016/s0162-3109(00)00188-0

(28) Xavier, A. S.; Lakshmanan, M. Delamanid: A New Armor in Combating Drug-Resistant Tuberculosis. *J Pharmacol Pharmacother* **2014**, *5* (3), 222–224. DOI:10.4103/0976-500X.136121.

(29) Seman, L.; Macha, S.; Nehmiz, G.; Simons, G.; Ren, B.; Pinnetti, S.; Woerle, H. J.; Dugi, K. Empagliflozin (BI 10773), a Potent and Selective SGLT2 Inhibitor, Induces Dose-Dependent Glucosuria in Healthy Subjects. *Clin. Pharmacol. Drug Dev.* **2013**, *2* (2), 152–161. DOI:10.1002/cpdd.16.

(30) Hauser, R. A.; Isaacson, S.; Clinch, T.; Ellenbogen, A.; Silver, D.; Struck, L.; Gil, R.; Pappert, E.; Jankovic, J.; Goldstein, S.; LeWitt, P.; Hull, K.; Thomas, M.; Danisi, F.; Factor, S.; Malaty, I.; Plotkin, G.; Thomas, K.; Vasquez, A.; Wierzbicki, T.; Dalvi, A.; Hutchman, R.; Shorr, R.; Fernandez, H.; Kreitzman, D. Randomized, Placebo-Controlled Trial of Trimethobenzamide to Control Nausea and Vomiting during Initiation and Continued Treatment with Subcutaneous Apomorphine Injection. *Parkinsonism Relat. Disord.* **2014**, *20* (11), 1171–1176. DOI:10.1016/j.parkreldis.2014.08.010.

(31) Brandes, J. L.; Saper, J. R.; Diamond, M.; Couch, J. R.; Lewis, D. W.; Schmitt, J.; Neto, W.; Schwabe, S.; Jacobs, D. Topiramate for Migraine Prevention A Randomized Controlled Tria. *JAMA,* **2004**, *291* (8), 965-973. DOI:10.1001/jama.291.8.965

(32) Yang, L. P. H.; Keam, S. J. Retapamulin A Review of Its Use in the Management of Impetigo AndOther Uncomplicated Superficial Skin Infections. *Drugs* **2008,** *68* (6), 855–873. DOI:10.2165/00003495-200868060-00008

(33) Pan, S.; Gray, N. S.; Gao, W.; Mi, Y.; Fan, Y.; Wang, X.; Tuntland, T.; Che, J.; Lefebvre, S.; Chen, Y.; Chu, A.; Hinterding, K.; Gardin, A.; End, P.; Heining, P.; Bruns, C.; Cooke, N. G.; Nuesslein-Hildesheim, B. Discovery of BAF312 (Siponimod), a Potent and Selective S1P Receptor Modulator. *ACS Med. Chem. Lett.* **2013**, *4* (3), 333–337. DOI:10.1021/ml300396r.

(34) Tseng, Y. T.; Chen, C. S.; Jong, Y. J.; Chang, F. R.; Lo, Y. C. Loganin Possesses Neuroprotective Properties, Restores SMN Protein and Activates Protein Synthesis Positive Regulator Akt/MTOR in Experimental Models of Spinal Muscular Atrophy. *Pharmacol. Res.* **2016**, *111,* 58–75 DOI:10.1016/j.phrs.2016.05.023.

(35) Tan, Q.; Zhu, Y.; Li, J.; Chen, Z.; Han, G. W.; Kufareva, I.; Li, T.; Ma, L.; Fenalti, G.; Li, J.; Zhang, W.; Xie, X.; Yang, H.; Jiang, H.; Cherezov, V.; Liu, H.; Stevens, R. C.; Zhao, Q.; Wu, B. Structure of the CCR5 Chemokine Receptor-HIV Entry Inhibitor Maraviroc Complex. *Science* **2013**, *341* (6152), 1387–1390. DOI:10.1126/science.1241475.

(36) Kim, W.; Lee, H.; Kim, S.; Joo, S.; Jeong, S.; Yoo, J. W.; Jung, Y. Sofalcone, a Gastroprotective Drug, Covalently Binds to KEAP1 to Activate Nrf2 Resulting in Anti-Colitic Activity. *Eur. J. Pharmacol.* **2019**, *865*. DOI:10.1016/j.ejphar.2019.172722.

(37) Van Custsem, E.; Cunningham, D.; Maroun, J.; Cervantes, A.; Glimelius, B. Raltitrexed: Current Clinical Status and Future Directions. *Ann. Oncol.* **2002**, *13* (4), 513–522. DOI:10.1093/annonc/mdf054.

(38) Asai, J.; Takenaka, H.; Katoh, N.; Kishimoto, S. Dibutyryl CAMP Influences Endothelial Progenitor Cell Recruitment during Wound Neovascularization. *Journal of Investigative Dermatology* **2006**, *126* (5), 1159–1167. DOI:10.1038/sj.jid.5700188.

(39) Linet, O. I.; Ogrinc, F. G. Efficacy and Safety of Intracavernosal Alprostadil in Men with Erectile Dysfunction. *New England Journal of Medicine* **1996**, *334* (14), 873–877. DOI:10.1056/nejm199604043341401.

(40) Ueda, T.; Takeno, S.; Hirakawa, K.; Furukido, K.; Yajin, K. Leukotriene Receptor Antagonist Pranlukast Suppresses Eosinophil Infiltration and Cytokine Production in Human Nasal Mucosa of Perennial Allergic Rhinitis. *Annals of Otology, Rhinology and Laryngology* **2003**, *112* (11), 955–961. DOI:10.1177/000348940311201107.

(41) Keam, S. J.; Lyseng-Williamson, K. A.; Goa, K. L.; Korenblat, P. E.; Lockey, R. F.; Obase, Y.; Rovati, G. E.; Sampson, A. P.; Smith, L. J.; Tamura, G. Pranlukast: A Review of Its Use in the Management of Asthma. *Drugs* **2003,** *63* (10), 991–1019. DOI:10.2165/00003495-200363100-00005.

(42) Whitson, J. T. Travoprost - A New Prostaglandin Analogue for the Treatment of Glaucoma. *Expert Opin Pharmacother* **2002**, *3* (7), 965–977. DOI:10.1517/14656566.3.7.965.

(43) Ren, H. Y.; Grove, D. E.; de La Rosa, O.; Houck, S. A.; Sopha, P.; van Goor, F.; Hoffman, B. J.; Cyr, D. M. VX-809 Corrects Folding Defects in Cystic Fibrosis Transmembrane Conductance Regulator Protein through Action on Membrane-Spanning Domain 1. *Mol. Biol. Cell* **2013**, *24* (19), 3016–3024. DOI:10.1091/mbc.E13-05-0240.

(44) Di-Wen, S.; Pan, G. Z.; Hao, L.; Zhang, J.; Xue, Q. Z.; Wang, P.; Yuan, Q. Z. Improved Antitumor Activity of Epirubicin-Loaded CXCR4-Targeted Polymeric Nanoparticles in Liver Cancers. *Int J Pharm* **2016**, *500* (1–2), 54-61. DOI:10.1016/j.ijpharm.2015.12.066.

(45) Malerba, M.; Radaeli, A.; Montuschi, P.; Morjaria, J. B. Vilanterol Trifenatate for the Treatment of COPD. *Expert Rev. Respir. Med.* **2016**, *10* (7), 719–731. DOI:10.1080/17476348.2016.1184976.

(46) Dobesh, P. P.; Oestreich, J. H. Ticagrelor: Pharmacokinetics, Pharmacodynamics, Clinical Efficacy, and Safety. *Pharmacotherapy* **2014**, *34* (10), 1077–1090. DOI:10.1002/phar.1477.

(47) Takehara, K.; Igarashi, A.; Ishibashi, Y. Dipyridamole Specifically Decreases Platelet-Derived Growth Factor Release from Platelets. *Pharmacology* **1990**, *40* (3), 150-156. DOI:10.1159/000138653.

(48) Burke, R. M.; Evans, J. D. Avanafil for Treatment of Erectile Dysfunction: Review of Its Potential. *Vascular Health and Risk Management* **2012,** *8*, 517-523. DOI:10.2147/VHRM.S26712.

(49) Lü, S., Wang, J. Homoharringtonine and omacetaxine for myeloid hematological malignancies. *J. Hematol. Oncol.* **2014**, *7* (2). DOI:10.1186/1756-8722-7-2

(50) Masuda, N.; Lee, S.-J.; Ohtani, S.; Im, Y.-H.; Lee, E.-S.; Yokota, I.; Kuroi, K.; Im, S.-A.; Park, B.-W.; Kim, S.-B.; Yanagita, Y.; Ohno, S.; Takao, S.; Aogi, K.; Iwata, H.; Jeong, J.; Kim, A.; Park, K.-H.; Sasano, H.; Ohashi, Y.; Toi, M. Adjuvant Capecitabine for Breast Cancer after Preoperative Chemotherapy. *New England Journal of Medicine* **2017**, *376* (22), 2147–2159. DOI:10.1056/nejmoa1612645.

(51) Kim, T. W.; Kang, Y. K.; Ahn, J. H.; Chang, H. M.; Yook, J. H.; Oh, S. T.; Kim, B. S.; Lee, J. S. Phase II Study of Capecitabine plus Cisplatin as First-Line Chemotherapy in Advanced Gastric Cancer. *Annals of Oncology* **2002**, *13* (12), 1893–1898. DOI:10.1093/annonc/mdf323.

(52) Craven, I.; Crellin, A.; Cooper, R.; Melcher, A.; Byrne, P.; Sebag-Montefiore, D. Preoperative Radiotherapy Combined with 5 Days per Week Capecitabine Chemotherapy in Locally Advanced Rectal Cancer. *Br. J. Cancer* **2007**, *97* (10), 1333–1337. DOI:10.1038/sj.bjc.6604042.

(53) Cotroneo, N.; Rubio, A.; Critchley, I. A.; Pillar, C.; Pucci, M. J. In Vitro and In Vivo Characterization of Tebipenem, an Oral Carbapenem. *Antimicrob. Agents Chemother.* **2020,** *64*(8), e02240-19. DOI:10.1128/AAC.02240-19.

(54) Rossi, M.; Roumeguère, T. Silodosin in the Treatment of Benign Prostatic Hyperplasia. *Drug Design, Development and Therapy* **2010**, *4*, 291–297. DOI:10.2147/DDDT.S10428.

(55) Sanad, M. H.; Saleh, G. M.; Marzook, F. A. Radioiodination and Biological Evaluation of Nizatidine as a New Highly Selective Radiotracer for Peptic Ulcer Disorder Detection. *J Labelled Comp. Radiopharm.* **2017**, *60* (13), 600-607. DOI:10.1002/jlcr.3541.

(56) Schwartz, T. L.; Siddiqui, U. A.; Stahl, S. M. Vilazodone: A Brief Pharmacological and Clinical Review of the Novel Serotonin Partial Agonist and Reuptake Inhibitor. *Ther. Adv. Psychopharmacol.* **2011**, *1* (3), 81–87. DOI:10.1177/2045125311409486.

(57) Ostojic, A.; Vrhovac, R.; Verstovsek, S. Ruxolitinib for the Treatment of Myelofibrosis: Its Clinical Potential. *Therapeutics and Clinical Risk Management* **2012**, *8*, 95–103.. DOI:10.2147/TCRM.S23277.

(58) Keating, M. J.; Kantarjian, H.; Talpaz, M.; Redman, J.; Koller, C.; Barlogie, B.; Velasquez, W.; Plunkett, W.; Freireich, E. J.; McCredie, K. B. Fludarabine: A New Agent with Major Activity against Chronic Lymphocytic Leukemia. *Blood* **1989**, *74* (1), 19-25. DOI:10.1182/blood.v74.1.19.19.

(59) Mittendorf, J.; Weigand, S.; Alonso-Alija, C.; Bischoff, E.; Feurer, A.; Gerisch, M.; Kern, A.; Knorr, A.; Lang, D.; Muenter, K.; Radtke, M.; Schirok, H.; Schlemmer, K. H.; Stahl, E.; Straub, A.; Wunder, F.; Stasch, J. P. Discovery of Riociguat (BAY 63-2521): A Potent, Oral Stimulator of Soluble Guanylate Cyclase for the Treatment of Pulmonary Hypertension. *ChemMedChem.* **2009**, *4* (5), 853-865. DOI:10.1002/cmdc.200900014.

(60) Kawamori, R.; Tajima, N.; Iwamoto, Y.; Kashiwagi, A.; Shimamoto, K.; Kaku, K. Voglibose for Prevention of Type 2 Diabetes Mellitus: A Randomised, Double-Blind Trial in Japanese Individuals with Impaired Glucose Tolerance. *The Lancet* **2009**, *373* (9675), 1607–1614. DOI:10.1016/S0140-6736(09)60222-1.

(61) Chapple, C. R.; Cardozo, L.; Nitti, V. W.; Siddiqui, E.; Michel, M. C. Mirabegron in Overactive Bladder: A Review of Efficacy, Safety, and Tolerability. *Neurourol. Urodyn.* **2014**, *33* (1), 17-30. DOI:10.1002/nau.22505.
